# A Scoping Review about the Characteristics and Success-Failure Rates of Temporary Anchorage Devices in Orthodontics

**DOI:** 10.3390/dj10050078

**Published:** 2022-05-06

**Authors:** Daniel Jaramillo-Bedoya, Gustavo Villegas-Giraldo, Andrés A. Agudelo-Suárez, Diana Milena Ramírez-Ossa

**Affiliations:** Faculty of Dentistry, University of Antioquia, Medellín 050010, Colombia; daniel.jaramillob@udea.edu.co (D.J.-B.); gustavo.villegasg@udea.edu.co (G.V.-G.)

**Keywords:** orthodontic anchorage procedures, orthodontic appliances, systematic review

## Abstract

This study synthesized the scientific evidence concerning the main characteristics of the Temporary Anchorage Devices (TADs) used in orthodontics and reported the success-failure rates during treatment. For that means, this scoping review collected articles from previous research. A complementary search was carried out in the databases PubMed-MEDLINE, Scopus, LILACS, and EMBASE, focusing on original studies published from 2010 to 2020. We analyzed the main characteristics of the publications. As a result, 103 articles were included. Most of the research was conducted among different groups, who needed TADs principally in the maxilla and an interradicular location between the second premolar and first molar. AbsoAnchor, Dentos Inc., Daegu, Korea, was the most used brand of TADs. The most common characteristics of the devices and biomechanics were a diameter and length of 1.6 mm and 8 mm, a self-drilled system, a closed technique for placement, immediate loading, and forces that ranged between 40 and 800 g. Of the studies, 47.6% showed success rates ≥90%. In conclusion, high success rates were found for TADs, and differences were found according to sociodemographic and clinical variables. The studies showed variability in methodological design, and scientific publications were concentrated in certain countries. We recommend further scientific research on TADs using more standardized designs.

## 1. Introduction

Controlling the reaction force during orthodontic treatment is necessary to avoid undesirable movements of the teeth. Even though many biomechanical alternatives have been developed to moderate anchorage, the use of Temporary Anchorage Devices (TADs) is currently one of the most popular among orthodontists [1]. TADs may be loaded immediately after placement, allow the operator to develop biomechanics for all types of movements, offer minimum risk of damage to the bone, roots, soft tissues, and other nearby structures, and do not depend on the collaboration of the patient [2,3].

TADs have developed in such a way that they can be used in an alveolar or extra-alveolar manner, reducing invasive surgical acts and achieving absolute anchorage with high success rates [4,5]. Nevertheless, several factors may limit their success [6,7] and there are many possibilities that may confuse clinicians seeking to select the best TAD depending on the patient, the procedure, and the biomechanics.

Despite the extensive studies carried out on TADs, the large amount of literature may create misunderstandings, and a comprehensive review of the information is necessary to make clinical decisions based on scientific evidence. Scoping reviews are studies that aim to describe the characteristics of the existing literature on a specific topic, without evaluating its quality, in order to generate hypotheses and questions for future research [8,9]. To the best of our knowledge, the information provided by the scientific literature is limited in the context of analyzing the specific characteristics of TADs. The purpose of this study was to synthesize the scientific evidence about the use of TADs, the main characteristics of the TADs that are used, and their success-failure rates during the orthodontic treatment.

## 2. Materials and Methods

For the purposes of this scoping review, the Preferred Reporting Items for Systematic Reviews and Meta-Analyses (PRISMA) method was followed, which was adapted for scoping reviews [10]. This manuscript also adopted the methodology proposed by the Joanna Briggs Institute [9] and the methodological framework for scoping studies [8]. According to the inclusion criteria proposed for systematic reviews, the protocol for scoping reviews should not be registered in the International prospective register of systematic reviews-PROSPERO. 

### 2.1. Research Question

This review posed the following population, concept, and context (PCC) question: What are the characteristics and success-failure rates of the use of TADs in orthodontic treatment according to the available scientific evidence?

- Population: People receiving orthodontic treatment.
- Concept: Temporary Anchorage Devices (TADs).
- Context: Characteristics and success-failure rates.

### 2.2. Search Process for Identifying Relevant Studies

The identification of original studies was part of a previous umbrella review [7]. Subsequently, a complementary comprehensive search was conducted for peer-reviewed literature to locate publications relevant to the research topic. Four electronic databases were included: PubMed-MEDLINE, Scopus, LILACS (Latin-American Scientific Literature in the Health Sciences), and EMBASE (the Excerpta Medica Database). The search syntaxis used in PubMed-MEDLINE was as follows: (((((((“Orthodontic Anchorage Procedures”[Mesh]) OR orthodontic mini implants[Title/Abstract]) OR mini-implant[Title/Abstract]) OR miniplate[Title/Abstract]) OR Temporary anchorage devices[Title/Abstract]) OR mini implant[Title/Abstract]) OR miniscrews[Title/Abstract]) OR orthodontic miniscrews[Title/Abstract]. For EMBASE, the syntaxis search were: ((‘orthodontic miniscrews’:ab,ti OR ‘miniscrews’:ab,ti OR ‘mini implant’:ab,ti OR ‘temporary anchorage devices’:ab,ti OR ‘miniplate’:ab,ti OR ‘mini-implant’:ab,ti OR ‘orthodontic mini implants’:ab,ti OR ‘orthodontic anchorage procedures’:ab,ti). These search syntaxes were adapted for the other electronic databases. The search was focused on original studies published in Spanish, English, and Portuguese, and published from 2010 to 2020. Letters to the editor, editorials, systematic and theoretical reviews, summaries of conferences, historical papers, and book summaries were excluded.

### 2.3. Study Screening and Selection

Two reviewers (D.J.-B. and G.V.-G.) searched independently to identify titles and abstracts of potentially eligible studies. Articles whose abstracts contained information that fit the eligibility criteria were selected for a full reading. To identify other sources of information, the research team searched the reference sections of those studies that were included, and all papers selected for inclusion in the review were processed for data extraction. All of these processes were supervised by the other members of the research team (A.A.A.-S. and D.M.R.-O.).

### 2.4. Collating, Summarizing, and Reporting Findings

The following variables were described for each study: title, journal, publication year, main author, country (first author), study type, device success or failure rate, sample characteristics (sex, age, and origin), intervention site, device characteristics (brand, device type, diameter, length, and system type), surgical technique, placement site, loading protocol, force applied, orthodontic movements type, treatment time, and others variables as objective(s), primary results, bias sources, limitations, and other relevant information.

The variables were categorized into four principal groups: (a) publication characteristics (author, journal, year, country, and study type); (b) sample characteristics (sex, age, origin, and intervention site); (c) TADs and biomechanical characteristics (brand, device type, diameter, length, system type, surgical technique, placement site, loading protocol, force applied, orthodontic movements type, and treatment time); and (d) device success or failure rate. Additional variables, including study objective(s), primary results, limitations, and other relevant information of the studies included in the present scoping review are reported in supplementary tables.

To group the results regarding the percent success rates of each type of TADs in different studies, the average of different samples was obtained using the following formula:

$$\bar{X}=\frac{C1P1+C2P2+C3P3\ldots }{C1+C2+C3}$$

where CxPx is the mean %, Px is the value of success rate, and Cx is the samples size. All values of the success rate are presented as the mean and standard deviation (±SD).

## 3. Results

### 3.1. Study Selection

**Figure 1:**
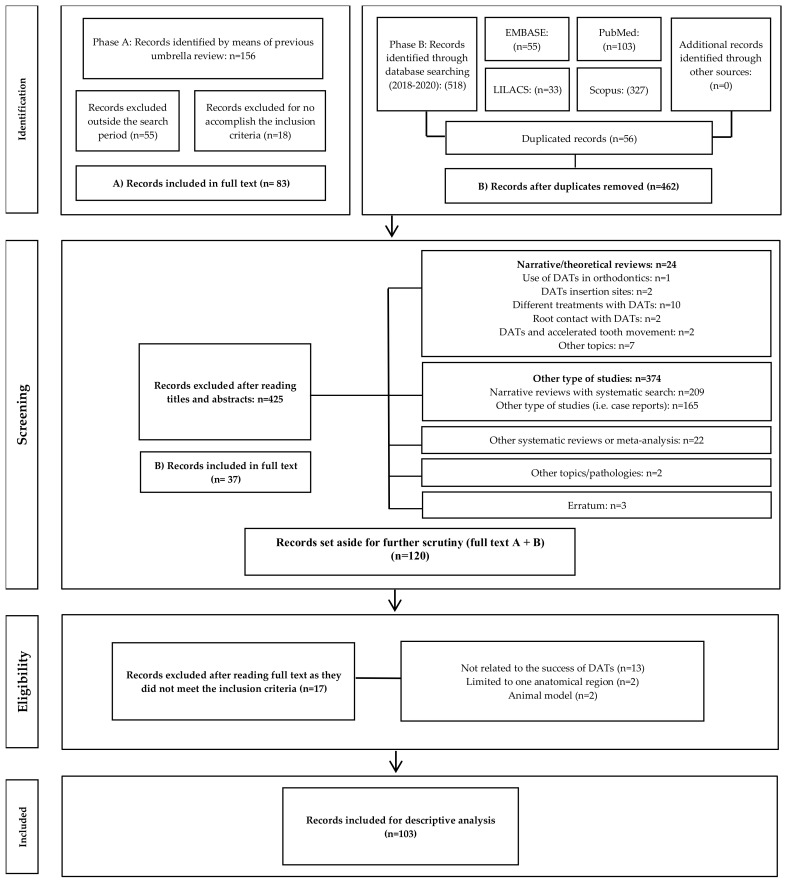
Selection process of studies for the scoping review.

During phase A of the study, records identified from previous umbrella reviews yielded 156 articles, of which 73 were excluded, and the remaining 83 were extracted for their full text to be read. During phase B, the initial search yielded 518 records; after eliminating duplicates, 462 records were selected for the revision of titles and abstracts. Of these, 425 records were excluded, and the remaining 37 were extracted for their full text to be read. After full-text reading, 17 articles were discarded. Finally, 103 articles were included [3,5,11,12,13,14,15,16,17,18,19,20,21,22,23,24,25,26,27,28,29,30,31,32,33,34,35,36,37,38,39,40,41,42,43,44,45,46,47,48,49,50,51,52,53,54,55,56,57,58,59,60,61,62,63,64,65,66,67,68,69,70,71,72,73,74,75,76,77,78,79,80,81,82,83,84,85,86,87,88,89,90,91,92,93,94,95,96,97,98,99,100,101,102,103,104,105,106,107,108,109,110,111]. The reasons for exclusion are summarized in Figure 1.

### 3.2. Publication Characteristics

Table 1 presents the main characteristics of the included studies. The first author who has published the most on this subject is Motoyoshi M. (3.9%, n = 4) [19,57,67,76], followed by Azeem M. [96,105], Elkordy S.A. [73,101], Ganzer N. [83,84], Manni A. [31,40], and Nienkemper M. [51,110] (1.9%, n = 2); the remaining studies (86.4%, n = 89) have been published by different authors [3,5,11,12,13,14,15,16,17,18,20,21,22,23,24,25,26,27,28,29,30,32,33,34,35,36,37,38,39,41,42,43,44,45,46,47,48,49,50,52,53,54,55,56,58,59,60,61,62,63,64,65,66,68,69,70,71,72,74,75,77,78,79,80,81,82,85,86,87,88,89,90,91,92,93,94,95,97,98,99,100,102,103,104,106,107,108,109,111].

Overall, the articles have been published in 47 journals, with the highest number of publications in *The American Journal of Orthodontics and Dentofacial Orthopedics* (n = 26) [14，^15^，^16^,^18^,^19^,^23^,^28^,^32^,^34^,^36^,^43^,^50^,^52^,^53^,^58^,^60^,^64^,^72^,^75^,^76^,^77^,^81^,^84^,^88^,^90^,^93^], *The Angle Orthodontist* (n = 13) [25,37,41,45,49,68,71,73,79,95,101,102,103], *The European Journal of Orthodontics* (n = 10) [17,30,31,35,47,61,63,70,80,83], *The Journal of Orofacial Orthopedics* (n = 6) [13,29,55,94,106,109], and *Progress in Orthodontics* (n = 6) [5,51,98,99,104,110], accounting for nearly 59.2% of all articles included in the present review. The remaining 40.8% articles have been published in 42 other journals, with 1–2 articles in each journal (Table S1, Supplementary Material). When grouped according to year, more studies have been published between 2010 and 2012 (36.9%, n = 38). In 2019 and 2020), however, few studies on this topic have been published (15.5%, n = 16) (Table 1, Table S1, Supplementary Material).

**Figure 2:**
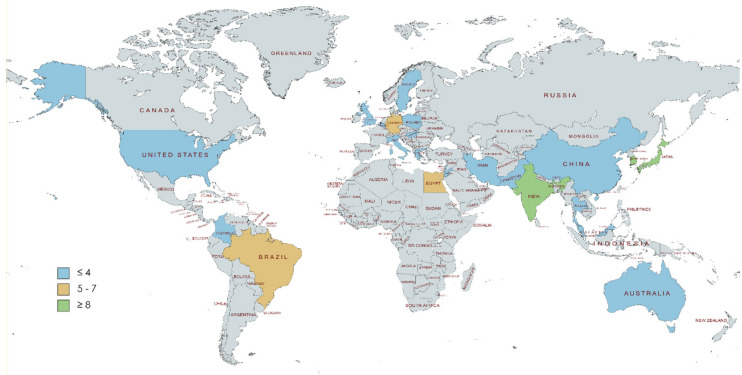
Publications by country. The grey color indicates that no studies are provided for the scoping review. Created with mapchart.net.

In general, majority of the scientific evidence has been accumulated in Asian countries (except for Turkey) (58%, n = 54) [3,12,14,15,16,17,18,19,21,22,27,28,30,32,33,34,36,37,38,41,42,49,50,52,53,54,56,57,59,60,61,62,64,65,66,67,69,74,75,76,78,81,82,85,90,93,96,97,102,103,104,105,107,111]. Additionally, Turkey [20,25,35,43,70,71,72,77,80,99,106], Germany [13,29,39,46,51,63,110], Egypt [23,55,73,79,86,89,101], and Brazil [11,24,48,98,100] have made notable contributions on the topic, together representing 29.1% of the studies included in this review (n = 30) (Figure 2).

Of all articles (n = 103) that met the selection criteria, 64.1% were observational studies (n = 66) [3,5,11,12,13,14,15,16,17,18,19,20,21,22,23,24,26,27,28,29,30,31,32,33,34,35,39,40,41,42,44,46,48,50,51,52,53,54,55,56,57,59,61,63,65,67,68,69,70,75,76,77,81,90,91,92,93,94,95,96,104,105,106,107,110,111] (Table S1, Supplementary Material). When the data were grouped according to the type of studies by their year of publication, observational studies were predominant during 2010–2015 (46.7%, n = 48; *p* < 0.01), whereas interventional studies became predominant during 2016–2020 (23.3%, n = 24; *p* < 0.01) (Table 1). 

### 3.3. Sample Characteristics

The sample characteristics of the included studies are summarized in Table 2. In all studies, the proportion of females was greater in the study population (84.4%, 4115 of 4873 patients). Moreover, 82 studies (79.6%) included patients under 25 years of age [3,5,12,15,17,18,20,21,23,26,27,28,29,30,31,32,33,35,36,37,38,39,40,41,42,45,46,47,48,49,50,51,52,53,54,55,56,57,58,59,60,62,63,64,65,66,67,69,70,71,72,73,74,75,76,77,78,79,80,82,83,84,86,87,88,89,91,93,94,95,96,98,99,101,102,103,104,105,106,107,109,110], 4 studies (3.9%) did not report patient age [25,43,92,100]. Regarding the origin of the sample, 84.4% recruited patients from university hospitals (n = 87) [3,5,12,14,15,17,18,19,20,21,22,23,24,25,26,27,28,30,32,33,34,35,36,37,38,41,43,45,46,47,48,49,50,51,52,53,54,55,56,57,58,59,60,61,62,63,64,65,66,67,68,69,71,72,73,74,75,76,77,78,79,80,84,85,86,87,88,89,90,91,92,93,94,96,97,99,100,101,102,103,105,106,107,108,109,110,111]. Regarding the site of TADs placement, 51.5% articles reported placement in the maxilla (n = 53) [3,5,11,12,14,15,16,20,21,23,24,27,28,29,35,36,37,38,39,41,43,45,46,47,48,50,51,55,57,60,62,64,66,67,71,72,74,75,76,77,81,83,84,86,87,88,90,93,94,96,97,107,108,109,110], and 2.1% articles did not report the placement site (n = 2) [80,99] (Table S2, Supplementary Material).

### 3.4. TADs and Biomechanical Characteristics

Regarding the main characteristics of TADs (Table 3), there was great variability in terms of brands, with AbsoAnchor (Dentos Inc., Daegu, Korea) being the most preferred one (19.4%, n = 20), followed by ISA Orthodontic Implants (Biodent, Tokyo, Japan) (6.8%, n = 7); other individual brands were used in <5% (together 73.8%, n = 76) (Table S3, Supplementary Material).

The terminology used to refer to TADs is chosen non-specifically [7], and there was noted little conformity on nomenclature during this revision. Nevertheless, we can focus the terms based on the classification proposed by Melsen [112] and Papadopoulos and Tarawneh [113]: Mini-screws (devices that are self-tapping and do not need osseointegration), mini-implants (developed smaller than dental implants), micro-implants and micro-screws (used to described implants or screws of the same dimension without any differentiation), and mini-plates (derived from surgical procedures classified according to their shape). From this perspective, four articles did not report the type of TADs used, and the remaining 99 articles reported the use of 13,385 TADs: 49% mini-screws (n = 6565), 30.9% mini-implants (n = 4135), 5.3% micro-implants (n = 713), 0.8% micro-screws (n = 95), and 14% mini-plates (n = 1877). Regarding device diameter and length, the most used dimensions were, respectively, 1.6 mm (25.2%, n = 26) and 8 mm (58.3%, n = 60). Regarding the type of system, the self-drilled system was the most used (57.2%, n = 59) (Table 3, Table S3, Supplementary Material). Furthermore, a closed surgical technique was used in 77.7% of the studies (n = 80) and 13.6% did not report the technique used (n = 14) (Table 3, Table S3, Supplementary Material).

The most common anatomical site of TADs placement was the interradicular space between the maxillary first molar and second premolar (48.6%, n = 50), and 35.9% articles (n = 37) reported placement at other anatomical sites, including the retromolar area, mandibular ramus and buccal shelf (Table 3, Table S3, Supplementary Material). The loading protocol was reported in 72.8% of the studies (n = 75). The force used in biomechanics was reported in 66% of the studies (n = 68). As such, diverse forces ranging from 40 to 800 g, were used depending on the movement and dispositive employed.

The most common therapeutic goal for the use of TADs was the en-masse retraction of anterior teeth (37.8%, n = 39), followed by molar distalization (17.5%, n = 18); the remaining studies reported various other goals, such as molar uprighting, gap closure, intrusion, and canine retraction, amongst others (44.7%, n = 46) (Table 3, Table S3, Supplementary Material).

Despite our attempts to specify the time of treatment, we were unable to evaluate this aspect, because some articles did not report these data, or because the reported information was ambiguous.

Importantly, inflammation around TADs, often associated with poor oral hygiene, pain, and device fracture, was the most common complication reported (14.5%, n = 15).

The primary limitations of the included studies were small sample size, retrospective nature of some studies (which limited information), poor patient compliance, and exclusion of patients due to TADs loosening or dislodging.

### 3.5. Success and Failure Rates

The success rate of TADs was reported in 70.9% of the studies (n = 73), considering this as an index for the survival of the device. In 47.6% of the studies, the overall success rate was ≥90%. The failure rate was reported in 19.4% of the studies (n = 20). In six studies, the failure rate was 10% to 26%, and in the remaining 14 studies, this rate was <10% (Table S3, Supplementary Material).

**Figure 3:**
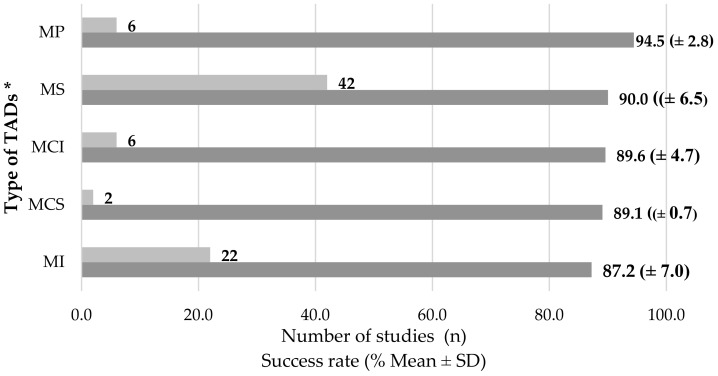
Summary of success rates according to type of TADs and considering the studies providing this topic. * MS: Mini-screw; MI: Mini-implant; MCI: Micro-implant; MCS: Micro-screw; MP: Mini-plate SD; Standard Deviation.

Regarding success rate by the type of TADs, a higher rate was reported for MPs (95 ± 3%) and a lower rate was reported for MIs (87 ± 7%) (Figure 3). Multiple chi-square tests were performed to establish the significance of differences amongst the different types of TADs, and *p* values of <0.001 were noted in all cases.

## 4. Discussion

Scoping reviews are a type of study derived from systematic reviews and designed to examine the existing literature that describes in detail the characteristics of a topic and maps the available evidence on it [114]. Therefore, we selected the approach of scoping review for our study to synthesize the available literature on the key characteristics of TADs used for orthodontic treatments and the success and failure rates of these devices. Given the abundance of data on this topic, we summarized the gathered information into groups according to publication characteristics, sample characteristics, TADs type, and treatment biomechanics.

Evidently, TADs have garnered much research interest. As such, of all studies included in the present scoping review, only four have been led by Motoyoshi M. [19,57,67,76], and the rest have been led by different authors. Similarly, a majority of the included articles have been published in journals with high impact, including the *American Journal of Orthodontics and Dentofacial Orthopedics, The Angle Orthodontist, the European Journal of Orthodontics, the Journal of Orofacial Orthopedics, and Progress in Orthodontics*, proving the accuracy of scientific methods and the reliability of results reported there.

Furthermore, skeletal anchorage started receiving great attention during the early 2010s, because a majority of the studies related to this topic were published until 2012; however, at the end of the decade, only a few studies on this topic were published. This trend may be associated with their high success rates and the accumulating knowledge on TADs. Alternatively, with increasing requirements for publication, it is now very challenging to conduct clinical trials.

Amongst the included types of studies, there were fewer clinical trials, which serve as the basis for further studies of greater analytical power. Specifically, of the 103 articles included in the present review, only 33.9% were clinical trials. However, according to the type of study by the year of publication, the number of clinical trials has evidently increased in recent years. This is an important finding, reflecting the growing interest of the academic community in conducting studies that are at the top levels of the pyramid of scientific evidence. Notably, in most studies, the participating patients were recruited from hospitals and academic institutions, perhaps because research is concentrated in universities, with occasional support from private practice organizations.

Regarding countries from where the studies were published, over half of the articles included in the present review were from Asia. Whilst global decisions can be made based on findings in specific populations, results obtained in certain groups cannot be generalized [7].

Of note, regarding sample characteristics, the majority of the patients (>84%) included in the reviewed studies were female. For instance, in studies by Takaki T [22], Moon CH [18], Tseng YC [111], respectively, 78%, 64%, and 78% of the sample participants were female. This sampling skew questions the conclusion drawn in previous studies that the success and failure rate of TADs is not sex-specific [7]. Therefore, given the evident bias in samples analyzed thus far, additional comparative studies between males and females may be required.

No clear consensus has yet been reached regarding the minimum age for the placement of TADs. According to the analysis, studies have been conducted principally in adolescents or in an adult population (15 years of age and older) with a percentage of 81.6%. However, some studies reported their use in patients as young as eight years for maxillary protraction, by virtue of the fact that patients under 10 years of age respond better to these types of procedures [98,115].

The relationship between the success rate and age also remains controversial, although the placement of TADs in the palatal area in patients up to eight years of age has resulted in a high success rate (≥90%) [15]. Nonetheless, most studies have reported that younger patients, especially those under the age of 15, had a higher failure rate than older age groups [7,15,116,117]. This could be attributed to a difference in bone density, which is lower in adolescents, poor oral hygiene, or in the placement location [104,117].

In terms of the placement site, it was found that the vast majority were placed in the maxilla. This placement can be associated with the fact that the biomechanics most used are the closure of spaces by retraction of protruding anterior teeth [42,78]. A reasonable explanation, after Class I malocclusion, the Class II malocclusion is the most prevalent [118], and one of the most common treatments with satisfactory results in such patients is the extraction of the upper premolars to camouflage this clinical condition [119,120].

In the present study, a great variety of brands was found, but the predominance of Asian manufacturers was evident, a finding that was consistent with the large production of related scientific literature in this part of the world [92,93,104,105]. The most common TADs were 1.6 mm in diameter and 8 mm in length, corresponding to the type of device normally selected for interradicular use between the second premolar and the first upper molar [84,97,102], which is a common placement site in orthodontic treatments with premolar extractions in patients with Class II malocclusion [74,81,82,104]. Nevertheless, it is also common to find higher diameters and lengths for the selection of extra-alveolar TADs, such as those placed on the infrazygomatic ridge, and used for reducing the risk of root damage [5,111].

Relative to the system type and surgical technique used to place the dispositive, the current and most frequent techniques found were self-drilled TADs and closed techniques because manufacturers have improved the device design to avoid additional surgical procedures and allow the orthodontist to safely place them during a clinical appointment [7].

We found five different types of TADs reported in the studies: mini-screws, mini-implants, micro-screws, micro-implants, and mini-plates. According to the authors of a previous study [7], these terms may have been chosen non-specifically, making the nomenclature potentially confusing for readers [6].

Our study verified the great versatility of TADs: they are used in all types of orthodontic movements, such as distalization, mesialization, retraction, intrusion, and extrusion, and with various biomechanics and highly variable loading protocols [92,105]. However, the fact cannot be ignored that, despite their great versatility and high success rates, TADs can present complications. These complications include inflammation, pain, fracture of the device, perforation of the maxillary sinus floor and root damage, among others [79,121,122].

Most of the studies reported success rates ≥ 90%. The mini-plate was the most successful type of TADs (94.5%), and the least successful was the mini-implant (87.2%). Although these success rates are high, the devices can fail, with failure rates falling between 5% and 26%. This rate is attributable to the large number of factors that influence the performance of these devices in orthodontic treatments, which depends not only on the characteristics of the dispositive but also on factors related to the patient, such as age, bone quality, and habits such as smoking. It also depends on factors related to the procedure, such as operator expertise, as well as factors associated with orthodontic treatment, such as the type of movement to be performed, the biomechanics used, and the activation force [5,7,123].

Finally, it should be noted that scoping reviews are considered a type of exploratory systematic review. Therefore, the objective of this study was to provide an overview of the literature and the variables analyzed. Although it might be expected to yield some type of statistical analysis, it is advisable to perform systematic reviews with meta-analyses in which the methodologies rely on homogeneous samples and are sectorized. However, we can safely assume that, based on the current review, it is possible to suggest the need for more clinical trials about skeletal anchorage and studies that focus on comparing the effectiveness of TADs according to sex as well as the identification of the most appropriate age for placement and the achievement of good results with the use of TADs. Also, it is important to conduct more studies on the mandible and to increase the analysis of extra-alveolar TADs, which are expanding their clinical use to camouflage Class II and Class III patients avoiding extraction of permanent teeth. There is a need to motivate the worldwide community to investigate and publish more about the use of TADs in different ethnic populations. It seems important to continue researching other variables and characteristics that were not deeply studied in the scientific evidence provided in the included articles. For instance, to assess the condition of the inserted bone and the amount of implantation in the cortical bone and cancellous bone through Computerized Tomography.

## 5. Conclusions

The main conclusions of this study were:

- There is a great deal of scientific evidence about temporary anchorage devices in orthodontics, as shown by the one hundred and three publications analyzed in this study from 95 authors, 47 journals, and 42 countries.
- These publications reported on the results of clinical trials, descriptive studies, and retrospective studies. Most of the research was conducted among females, adolescents, and the adult population, who needed TADs principally in the maxilla and in an interradicular location between the second premolar and first molar, and attended university hospitals for en-masse retraction of anterior teeth.
- AbsoAnchor, made by Dentos Inc., Daegu, Korea, was the most commonly used brand of TADs. The most common characteristics of the devices and biomechanics were a diameter and length of 1.6 mm and 8 mm, a self-drilled system, a closed technique for placement, immediate loading, and forces that ranged between 40 and 800 g.
- Although the success rates were high, reaching levels above 90%, complications can cause failures such as inflammation, pain, and fracture of the device. The most successful type of TADs was the mini-plate, while the least successful was the mini-implant.

## Figures and Tables

**Table 1:** Summary of publication characteristics (n = 103).

Characteristics	Total (n)		%	
***First author***				
Motoyoshi M	4		3.9	
Azeem M	2		1.9	
Elkordy SA	2		1.9	
Ganzer N	2		1.9	
Manni A	2		1.9	
Nienkemper M	2		1.9	
Other authors	89		86.4	
***Journal***				
American Journal of Orthodontics and Dentofacial Orthopedics	26		25.2	
The Angle Orthodontist	13		12.6	
European Journal of Orthodontics	10		9.8	
Journal of Orofacial Orthopedics	6		5.8	
Progress in Orthodontics	6		5.8	
Other Journals	42		40.8	
***Year***				
2010–2012	38		36.9	
2013–2015	23		22.3	
2016–2018	26		25.3	
2019–2020	16		15.5	
***Country***				
South Korea	15		14.6	
Japan	14		13.6	
Turkey	11		10.7	
India	10		9.7	
Other Asian countries	15		14.6	
Germany	7		6.8	
Egypt	6		5.8	
Brazil	5		4.9	
Other countries	20		19.3	
***Study type***				
**Interventional studies**	37		35.8	
Clinical trials	35		33.9	
Non-randomized trials	2		1.9	
**Observational studies**	66		64.1	
Descriptive	25		24.3	
Retrospective	23		22.3	
Prospective	11		10.7	
Cohort	4		3.9	
Cross sectional	3		2.9	
Study type per year*	***2010–2015***		***2016–2020***	
	***Total (n)***	***%***	***Total (n)***	***%***
Interventional studies	13	12.6	24	23.3
Observational studies	48	46.6	18	17.5

* *p*-value < 0.01.

**Table 2:** Summary of publication characteristics (n = 103).

Characteristics	Total (n)	%
*Sex* *		
Female	4115	84.4
Male	758	15.6
***Age***		
Under 15 years	19	18.4
15–20 years	26	25.3
20–25 years	35	34
Over 25 years	19	18.4
Not reported	4	3.9
***Origin***		
University hospital	87	84.4
Private practice	7	6.8
Not reported	9	8.8
***Intervention site***		
Maxilla	56	54.3
Mandible	10	9.7
Maxilla and mandible	35	33.9
Not reported	2	2.1

* It refers to the total of subjects included in the studies.

**Table 3:** Summary of TADs and biomechanical characteristics (n = 103).

Characteristic	Total (*n)*	%
***Brand***		
AbsoAnchor, Dentos Inc., Daegu, Korea	20	19.4
ISA Orthodontic Implants, Biodent, Tokyo, Japan	7	6.8
Other brands	76	73.8
Device type *		
Mini-screws	6565	49
Mini-implants	4135	30.9
Micro-implants	713	5.3
Micro-screws	95	0.8
Mini-plates	1877	14
***Diameter***		
1.6 mm	26	25.2
2 mm	17	16.5
Other diameters	60	58.3
***Length***		
8 mm	60	58.3
10 mm	18	17.4
Other lengths	25	24.3
***System type***		
Self-drilled	59	57.2
Self-tapped	44	42.8
***Surgical technique***		
Closed technique	80	77.7
Open technique	7	6.8
Both techniques	2	1.9
Not reported	14	13.6
***Placement site***		
Interradicular	50	48.6
Palatal	9	8.7
Infracygomatic crest	7	6.8
Other sites	37	35.9
***Loading protocol***		
Immediate	37	35.9
Postponed	31	30.1
Both protocols	7	6.8
Not reported	28	27.2
***Force***		
MS, MI, MCI, MCS * 40–250 g	68	66.0
MP * 300–800 g		
Not reported	35	34.0
***Orthodontic movements type***		
En-masse retraction of anterior teeth	39	37.8
Molar distalization	18	17.5
Other movements	46	44.7

* Refers to the total of subjects included in the studies.

## Data Availability

Not applicable.

## Supplementary Materials

The following supporting information can be downloaded at: https://www.mdpi.com/article/10.3390/dj10050078/s1; Table S1: List of cited studies and their publication characteristics (n = 103) [11,12,13,14,15,16,17,18,19,20,21,22,23,24,25,26,27,28,29,30,31,32,33,34,35,36,37,38,39,40,41,42,43,44,45,46,47,48,49,50,51,52,53,54,55,56,57,58,59,60,61,62,63,64,65,66,67,68,69,70,71,72,73,74,75,76,77,78,79,80,81,82,83,84,85,86,87,88,89,90,91,92,93,94,95,96,97,98,99,100,101,102,103,104,105,106,107,108,109,110,111]; Table S2: List of cited studies and their sample characteristics (n = 103); Table S3: List of cited studies and TADs characteristics (n = 103); Table S4: List of cited studies and biomechanical characteristics of treatments (n = 103).

## References

[1] Barthélemi S., Desoutter A., Souaré F., Cuisinier F. (2019). Effectiveness of anchorage with temporary anchorage devices during anterior maxillary tooth retraction: A randomized clinical trial. Korean J. Orthod..

[2] Garfinkle J.S., Cunningham L.L., Beeman C.S., Kluemper G.T., Hicks E.P., Kim M.O. (2008). Evaluation of orthodontic mini-implant anchorage in premolar extraction therapy in adolescents. Am. J. Orthod. Dentofac. Orthop..

[3] Chen G., Chen S., Zhang X.Y., Jiang R.P., Liu Y., Shi F.H., Xu T.M. (2015). A new method to evaluate the positional stability of a self-drilling miniscrew. Orthod. Craniofac. Res..

[4] Berens A., Wiechmann D., Dempf R. (2006). Mini- and micro-screws for temporary skeletal anchorage in orthodontic therapy. J. Orofac. Orthop..

[5] Uribe F., Mehr R., Mathur A., Janakiraman N., Allareddy V. (2015). Failure rates of mini-implants placed in the infrazygomatic region. Prog. Orthod..

[6] Cunha A.C., da Veiga A.M.A., Masterson D., Mattos C.T., Nojima L.I., Nojima M.C.G., Maia L.C. (2017). How do geometry-related parameters influence the clinical performance of orthodontic mini-implants? A systematic review and meta-analysis. Int. J. Oral. Maxillofac. Surg..

[7] Ramírez-Ossa D.M., Escobar-Correa N., Ramírez-Bustamante M.A., Agudelo-Suárez A.A. (2020). An Umbrella Review of the Effectiveness of Temporary Anchorage Devices and the Factors That Contribute to Their Success or Failure. J. Evid. Based Dent. Pract..

[8] Levac D., Colquhoun H., O’Brien K.K. (2010). Scoping studies: Advancing the methodology. Implement. Sci..

[9] Peters M., Godfrey C., McInerney P., Munn Z., Tricco A., Khalil H., Aromataris E.Z.M. (2020). Chapter 11: Scoping Reviews (2020 version). JBI Manual for Evidence Synthesis.

[10] Tricco A.C., Lillie E., Zarin W., O’Brien K.K., Colquhoun H., Levac D., Moher D., Peters M.D.J., Horsley T., Weeks L. (2018). PRISMA Extension for Scoping Reviews (PRISMA-ScR): Checklist and Explanation. Ann. Intern. Med..

[11] Blaya M.G., Blaya D.S., Guimarães M.B., Hirakata L.M., Marquezan M. (2010). Patient’s perception on mini-screws used for molar distalization. Rev. Odonto. Ciênc..

[12] Basha A.G., Shantaraj R., Mogegowda S.B. (2010). Comparative study between conventional en-masse retraction (sliding mechanics) and en-masse retraction using orthodontic micro implant. Implant. Dent..

[13] Bayat E., Bauss O. (2010). Effect of smoking on the failure rates of orthodontic miniscrews. J. Orofac. Orthop..

[14] Kim S.H., Kang S.M., Choi Y.S., Kook Y.A., Chung K.R., Huang J.C. (2010). Cone-beam computed tomography evaluation of mini-implants after placement: Is root proximity a major risk factor for failure?. Am. J. Orthod. Dentofac. Orthop..

[15] Kim Y.H., Yang S.M., Kim S., Lee J.Y., Kim K.E., Gianelly A.A., Kyung S.H. (2010). Midpalatal miniscrews for orthodontic anchorage: Factors affecting clinical success. Am. J. Orthod. Dentofac. Orthop..

[16] Lee S.J., Ahn S.J., Lee J.W., Kim S.H., Kim T.W. (2010). Survival analysis of orthodontic mini-implants. Am. J. Orthod Dentofac. Orthop.

[17] Miyazawa K., Kawaguchi M., Tabuchi M., Goto S. (2010). Accurate pre-surgical determination for self-drilling miniscrew implant placement using surgical guides and cone-beam computed tomography. Eur. J. Orthod..

[18] Moon C.H., Park H.K., Nam J.S., Im J.S., Baek S.H. (2010). Relationship between vertical skeletal pattern and success rate of orthodontic mini-implants. Am. J. Orthod. Dentofac. Orthop..

[19] Motoyoshi M., Uemura M., Ono A., Okazaki K., Shigeeda T., Shimizu N. (2010). Factors affecting the long-term stability of orthodontic mini-implants. Am. J. Orthod. Dentofac. Orthop..

[20] Nalçaci R., Biçakçi A.A., Ozan F. (2010). Noncompliance screw supported maxillary molar distalization in a parallel manner. Kjod.

[21] Saxena R., Kumar P.S., Upadhyay M., Naik V. (2010). A clinical evaluation of orthodontic mini-implants as intraoral anchorage for the intrusion of maxillary anterior teeth. World J. Orthod..

[22] Takaki T., Tamura N., Yamamoto M., Takano N., Shibahara T., Yasumura T., Nishii Y., Sueishi K. (2010). Clinical study of temporary anchorage devices for orthodontic treatment—Stability of micro/mini-screws and mini-plates: Experience with 455 cases. Bull. Tokyo Dent. Coll..

[23] Aboul-Ela S.M., El-Beialy A.R., El-Sayed K.M., Selim E.M., El-Mangoury N.H., Mostafa Y.A. (2011). Miniscrew implant-supported maxillary canine retraction with and without corticotomy-facilitated orthodontics. Am. J. Orthod. Dentofac. Orthop..

[24] Alves M., Baratieri C., Nojima L.I. (2011). Assessment of mini-implant displacement using cone beam computed tomography. Clin. Oral. Implant. Res..

[25] Aydoğdu E., Özsoy Ö.P. (2011). Effects of mandibular incisor intrusion obtained using a conventional utility arch vs. bone anchorage. Angle Orthod..

[26] Buschang P.H., Carrillo R., Rossouw P.E. (2011). Orthopedic correction of growing hyperdivergent, retrognathic patients with miniscrew implants. J. Oral Maxillofac. Surg..

[27] Lee A.Y., Kim Y.H. (2011). Comparison of Movement of the Upper Dentition According to Anchorage Method: Orthodontic Mini-Implant versus Conventional Anchorage Reinforcement in Class I Malocclusion. ISRN Dent..

[28] Lee K.J., Park Y.C., Hwang C.J., Kim Y.J., Choi T.H., Yoo H.M., Kyung S.H. (2011). Displacement pattern of the maxillary arch depending on miniscrew position in sliding mechanics. Am. J. Orthod. Dentofac. Orthop..

[29] Lehnen S., McDonald F., Bourauel C., Jäger A., Baxmann M. (2011). Expectations, acceptance and preferences of patients in treatment with orthodontic mini-implants: Part II: Implant removal. J. Orofac. Orthop..

[30] Lim H.J., Choi Y.J., Evans C.A., Hwang H.S. (2011). Predictors of initial stability of orthodontic miniscrew implants. Eur. J. Orthod..

[31] Manni A., Cozzani M., Tamborrino F., De Rinaldis S., Menini A. (2011). Factors influencing the stability of miniscrews. A retrospective study on 300 miniscrews. Eur. J. Orthod..

[32] Oh Y.H., Park H.S., Kwon T.G. (2011). Treatment effects of microimplant-aided sliding mechanics on distal retraction of posterior teeth. Am. J. Orthod. Dentofac. Orthop..

[33] Sharma P., Valiathan A., Sivakumar A. (2011). Success rate of microimplants in a university orthodontic clinic. ISRN Surg..

[34] Suzuki E.Y., Suzuki B. (2011). Placement and removal torque values of orthodontic miniscrew implants. Am. J. Orthod. Dentofac. Orthop..

[35] Türköz C., Ataç M.S., Tuncer C., Balos Tuncer B., Kaan E. (2011). The effect of drill-free and drilling methods on the stability of mini-implants under early orthodontic loading in adolescent patients. Eur. J. Orthod..

[36] Al Maaitah E.F., Safi A.A., Abdelhafez R.S. (2012). Alveolar bone density changes around miniscrews: A prospective clinical study. Am. J. Orthod. Dentofac. Orthop..

[37] Ge Y.S., Liu J., Chen L., Han J.L., Guo X. (2012). Dentofacial effects of two facemask therapies for maxillary protraction. Angle Orthod..

[38] Gupta N., Kotrashetti S.M., Naik V. (2012). A comparitive clinical study between self tapping and drill free screws as a source of rigid orthodontic anchorage. J. Maxillofac. Oral. Surg..

[39] Jung B.A., Kunkel M., Göllner P., Liechti T., Wagner W., Wehrbein H. (2012). Prognostic parameters contributing to palatal implant failures: A long-term survival analysis of 239 patients. Clin. Oral Implant. Res..

[40] Manni A., Pasini M., Mauro C. (2012). Comparison between Herbst appliances with or without miniscrew anchorage. Dent. Res. J..

[41] Min K.I., Kim S.C., Kang K.H., Cho J.H., Lee E.H., Chang N.Y., Chae J.M. (2012). Root proximity and cortical bone thickness effects on the success rate of orthodontic micro-implants using cone beam computed tomography. Angle Orthod..

[42] Samrit V., Kharbanda O.P., Duggal R., Seith A., Malhotra V. (2012). Bone density and miniscrew stability in orthodontic patients. Aust. Orthod. J..

[43] Senışık N.E., Türkkahraman H. (2012). Treatment effects of intrusion arches and mini-implant systems in deepbite patients. Am. J. Orthod. Dentofac. Orthop..

[44] Topouzelis N., Tsaousoglou P. (2012). Clinical factors correlated with the success rate of miniscrews in orthodontic treatment. Int. J. Oral. Sci..

[45] Upadhyay M., Yadav S., Nagaraj K., Uribe F., Nanda R. (2012). Mini-implants vs fixed functional appliances for treatment of young adult Class II female patients: A prospective clinical trial. Angle Orthod..

[46] Ziebura T., Flieger S., Wiechmann D. (2012). Mini-implants in the palatal slope—A retrospective analysis of implant survival and tissue reaction. Head Face Med..

[47] Davoody A.R., Posada L., Utreja A., Janakiraman N., Neace W.P., Uribe F., Nanda R. (2013). A prospective comparative study between differential moments and miniscrews in anchorage control. Eur. J. Orthod..

[48] Janson G., Gigliotti M.P., Estelita S., Chiqueto K. (2013). Influence of miniscrew dental root proximity on its degree of late stability. Int. J. Oral. Maxillofac. Surg..

[49] Bechtold T.E., Kim J.W., Choi T.H., Park Y.C., Lee K.J. (2013). Distalization pattern of the maxillary arch depending on the number of orthodontic miniscrews. Angle Orthod..

[50] Jung Y.R., Kim S.C., Kang K.H., Cho J.H., Lee E.H., Chang N.Y., Chae J.M. (2013). Placement angle effects on the success rate of orthodontic microimplants and other factors with cone-beam computed tomography. Am. J. Orthod. Dentofac. Orthop..

[51] Nienkemper M., Wilmes B., Pauls A., Drescher D. (2013). Maxillary protraction using a hybrid hyrax-facemask combination. Prog. Orthod..

[52] Shinohara A., Motoyoshi M., Uchida Y., Shimizu N. (2013). Root proximity and inclination of orthodontic mini-implants after placement: Cone-beam computed tomography evaluation. Am. J. Orthod. Dentofac. Orthop..

[53] Suzuki M., Deguchi T., Watanabe H., Seiryu M., Iikubo M., Sasano T., Fujiyama K., Takano-Yamamoto T. (2013). Evaluation of optimal length and insertion torque for miniscrews. Am. J. Orthod. Dentofac. Orthop..

[54] Watanabe H., Deguchi T., Hasegawa M., Ito M., Kim S., Takano-Yamamoto T. (2013). Orthodontic miniscrew failure rate and root proximity, insertion angle, bone contact length, and bone density. Orthod. Craniofac. Res..

[55] El-Dawlatly M.M., Abou-El-Ezz A.M., El-Sharaby F.A., Mostafa Y.A. (2014). Zygomatic mini-implant for Class II correction in growing patients. J. Orofac. Orthop..

[56] Lai T.-T., Chen M.-H. (2014). Factors affecting the cl.linical success of orthodontic anchorage: Experience with 266 temporary anchorage devices. J. Dent. Sci..

[57] Motoyoshi M., Uchida Y., Matsuoka M., Inaba M., Iwai H., Karasawa Y., Shimizu N. (2014). Assessment of damping capacity as an index of root proximity in self-drilling orthodontic mini-implants. Clin. Oral Investig..

[58] Sandler J., Murray A., Thiruvenkatachari B., Gutierrez R., Speight P., O’Brien K. (2014). Effectiveness of 3 methods of anchorage reinforcement for maximum anchorage in adolescents: A 3-arm multicenter randomized clinical trial. Am. J. Orthod. Dentofac. Orthop..

[59] Shigeeda T. (2014). Root proximity and stability of orthodontic anchor screws. J. Oral Sci..

[60] Son S., Motoyoshi M., Uchida Y., Shimizu N. (2014). Comparative study of the primary stability of self-drilling and self-tapping orthodontic miniscrews. Am. J. Orthod. Dentofac. Orthop..

[61] Yoo S.H., Park Y.C., Hwang C.J., Kim J.Y., Choi E.H., Cha J.Y. (2014). A comparison of tapered and cylindrical miniscrew stability. Eur. J. Orthod..

[62] Victor D., Prabhakar R., Karthikeyan M.K., Saravanan R., Vanathi P., Vikram N.R., Reddy P.A., Sudeepthi M. (2014). Effectiveness of mini implants in three-dimensional control during retraction—A clinical study. J. Clin. Diagn. Res..

[63] Bremen J., Ludwig B., Ruf S. (2015). Anchorage loss due to Herbst mechanics-preventable through miniscrews?. Eur. J. Orthod..

[64] Iwai H., Motoyoshi M., Uchida Y., Matsuoka M., Shimizu N. (2015). Effects of tooth root contact on the stability of orthodontic anchor screws in the maxilla: Comparison between self-drilling and self-tapping methods. Am. J. Orthod. Dentofac. Orthop..

[65] Jeong J.W., Kim J.W., Lee N.K., Kim Y.K., Lee J.H., Kim T.W. (2015). Analysis of time to failure of orthodontic mini-implants after insertion or loading. J. Korean Assoc. Oral Maxillofac. Surg..

[66] Miresmaeili A., Sajedi A., Moghimbeigi A., Farhadian N. (2015). Three-dimensional analysis of the distal movement of maxillary 1st molars in patients fitted with mini-implant-aided trans-palatal arches. Korean J. Orthod..

[67] Motoyoshi M., Sanuki-Suzuki R., Uchida Y., Saiki A., Shimizu N. (2015). Maxillary sinus perforation by orthodontic anchor screws. J. Oral Sci..

[68] Sarul M., Minch L., Park H.S., Antoszewska-Smith J. (2015). Effect of the length of orthodontic mini-screw implants on their long-term stability: A prospective study. Angle Orthod..

[69] Yi Lin S., Mimi Y., Ming Tak C., Kelvin Weng Chiong F., Hung Chew W. (2015). A study of success rate of miniscrew implants as temporary anchorage devices in singapore. Int. J. Dent..

[70] Ağlarcı C., Esenlik E., Fındık Y. (2016). Comparison of short-term effects between face mask and skeletal anchorage therapy with intermaxillary elastics in patients with maxillary retrognathia. Eur. J. Orthod..

[71] Aras I., Tuncer A.V. (2016). Comparison of anterior and posterior mini-implant-assisted maxillary incisor intrusion: Root resorption and treatment efficiency. Angle Orthod..

[72] Duran G.S., Görgülü S., Dindaroğlu F. (2016). Three-dimensional analysis of tooth movements after palatal miniscrew-supported molar distalization. Am. J. Orthod. Dentofac. Orthop..

[73] Elkordy S.A., Abouelezz A.M., Fayed M.M., Attia K.H., Ishaq R.A., Mostafa Y.A. (2016). Three-dimensional effects of the mini-implant-anchored Forsus Fatigue Resistant Device: A randomized controlled trial. Angle Orthod..

[74] Khan B.I., Singaraju G.S., Mandava P., Reddy G.V., Nettam V., Bhavikati V.N. (2016). Comparison of Anchorage Pattern under Two Types of Orthodontic Mini- Implant Loading During Retraction in Type A Anchorage Cases. J. Clin. Diagn. Res..

[75] Lee M.Y., Park J.H., Kim S.C., Kang K.H., Cho J.H., Cho J.W., Chang N.Y., Chae J.M. (2016). Bone density effects on the success rate of orthodontic microimplants evaluated with cone-beam computed tomography. Am. J. Orthod. Dentofac. Orthop..

[76] Motoyoshi M., Uchida Y., Inaba M., Ejima K., Honda K., Shimizu N. (2016). Are assessments of damping capacity and placement torque useful in estimating root proximity of orthodontic anchor screws?. Am. J. Orthod. Dentofac. Orthop..

[77] Canan S., Şenışık N.E. (2017). Comparison of the treatment effects of different rapid maxillary expansion devices on the maxilla and the mandible. Part 1: Evaluation of dentoalveolar changes. Am. J. Orthod. Dentofac. Orthop..

[78] Chopra S.S., Mukherjee M., Mitra R., Kochar G.D., Kadu A. (2017). Comparative evaluation of anchorage reinforcement between orthodontic implants and conventional anchorage in orthodontic management of bimaxillary dentoalveolar protrusion. Med. J. Armed Forces India.

[79] Eissa O., El-Shennawy M., Gaballah S., El-Meehy G., El Bialy T. (2017). Treatment outcomes of Class II malocclusion cases treated with miniscrew-anchored Forsus Fatigue Resistant Device: A randomized controlled trial. Angle Orthod..

[80] Tunçer N.I., Arman-Özçirpici A., Oduncuoglu B.F., Göçmen J.S., Kantarci A. (2017). Efficiency of piezosurgery technique in miniscrew supported en-masse retraction: A single-centre, randomized controlled trial. Eur. J. Orthod..

[81] Watanabe T., Miyazawa K., Fujiwara T., Kawaguchi M., Tabuchi M., Goto S. (2017). Insertion torque and Periotest values are important factors predicting outcome after orthodontic miniscrew placement. Am. J. Orthod. Dentofac. Orthop..

[82] Ashith M., Shetty B., Shekatkar Y., Mangal U., Mithun K. (2018). Assessment of immediate loading with mini-implant anchorage in critical anchorage cases between stainless steel versus titanium miniscrew implants: A controlled clinical trial. Biomed. Pharmacol. J..

[83] Ganzer N., Feldmann I., Petrén S., Bondemark L. (2019). A cost-effectiveness analysis of anchorage reinforcement with miniscrews and molar blocks in adolescents: A randomized controlled trial. Eur. J. Orthod..

[84] Ganzer N., Feldmann I., Bondemark L. (2018). Anchorage reinforcement with miniscrews and molar blocks in adolescents: A randomized controlled trial. Am. J. Orthod. Dentofac. Orthop..

[85] Martires S., Kamat N.V., Dessai S.R. (2018). A CBCT evaluation of molar uprighting by conventional versus microimplant-assisted methods: An in-vivo study. Dent. Press J. Orthod..

[86] Abohabib A.M., Fayed M.M., Labib A.H. (2018). ffects of low-intensity laser therapy on the stability of orthodontic mini-implants: A randomised controlled clinical trial. J. Orthod..

[87] Bollero P., Di Fazio V., Pavoni C., Cordaro M., Cozza P., Lione R. (2018). Titanium alloy vs. stainless steel miniscrews: An in vivo split-mouth study. Eur. Rev. Med. Pharmacol. Sci..

[88] Łyczek J., Kawala B., Antoszewska-Smith J. (2018). Influence of antibiotic prophylaxis on the stability of orthodontic microimplants: A pilot randomized controlled trial. Am. J. Orthod. Dentofac. Orthop..

[89] Aly S.A., Alyan D., Fayed M.S., Alhammadi M.S., Mostafa Y.A. (2018). Success rates and factors associated with failure of temporary anchorage devices: A prospective clinical trial. J. Investig. Clin. Dent..

[90] Uesugi S., Kokai S., Kanno Z., Ono T. (2018). Stability of secondarily inserted orthodontic miniscrews after failure of the primary insertion for maxillary anchorage: Maxillary buccal area vs midpalatal suture area. Am. J. Orthod. Dentofac. Orthop..

[91] Van Hevele J., Nout E., Claeys T., Meyns J., Scheerlinck J., Politis C. (2018). Bone-anchored maxillary protraction to correct a class III skeletal relationship: A multicenter retrospective analysis of 218 patients. J. Craniomaxillofac. Surg..

[92] Gurdan Z., Szalma J. (2018). Evaluation of the success and complication rates of self-drilling orthodontic mini-implants. Niger. J. Clin. Pract..

[93] Jia X., Chen X., Huang X. (2018). Influence of orthodo.ontic mini-implant penetration of the maxillary sinus in the infrazygomatic crest region. Am. J. Orthod. Dentofac. Orthop..

[94] Di Leonardo B., Ludwig B., Lisson J.A., Contardo L., Mura R., Hourfar J. (2018). Insertion torque values and success rates for paramedian insertion of orthodontic mini-implants: A retrospective study. J. Orofac. Orthop..

[95] Lam R., Goonewardene M.S., Allan B.P., Sugawara J. (2018). Success rates of a skeletal anchorage system in orthodontics: A retrospective analysis. Angle Orthod..

[96] Azeem M., Haq A.U., Awaisi Z.H., Saleem M.M., Tahir M.W., Liaquat A. (2019). Failure rates of miniscrews inserted in the maxillary tuberosity. Dent. Press J. Orthod..

[97] Sabzijati M., Rahbar M., Shanei F., Salehi-Vaziri A., Ghaffari H.A., Abtahi S.-A. (2019). Comparing the Clinical Success Rate of Self-Drilling and Self-Tapping Mini-screws in the Retraction of Maxillary Anterior Teeth. Pesqui Bras. Odontopediatria Clín. Integr..

[98] De Souza R.A., Rino Neto J., de Paiva J.B. (2019). Maxillary protraction with rapid maxillary expansion and facemask versus skeletal anchorage with mini-implants in class III patients: A non-randomized clinical trial. Prog. Orthod..

[99] Calik Koseler B., Yilanci H., Ramoglu S.I. (2019). Does audiovisual information affect anxiety and perceived pain levels in miniscrew application?—A within-person randomized controlled trial. Prog. Orthod..

[100] Marañón-Vásquez G.A., Lagravère M.O., Borsatto M.C., de Souza S.S., Watanabe P.C.A., Matsumoto M.A.N., Saraiva M., Romano F.L. (2019). Effect of photobiomodulation on the stability and displacement of orthodontic mini-implants submitted to immediate and delayed loading: A clinical study. Lasers Med. Sci..

[101] Elkordy S.A., Abouelezz A.M., Fayed M.M.S., Aboulfotouh M.H., Mostafa Y.A. (2019). Evaluation of the miniplate-anchored Forsus Fatigue Resistant Device in skeletal Class II growing subjects: A randomized controlled trial. Angle Orthod..

[102] Park H.J., Choi S.H., Choi Y.J., Park Y.B., Kim K.M., Yu H.S. (2019). A prospective, split-mouth, clinical study of orthodontic titanium miniscrews with machined and acid-etched surfaces. Angle Orthod..

[103] Sivarajan S., Doss J.G., Papageorgiou S.N., Cobourne M.T., Wey M.C. (2019). Mini-implant supported canine retraction with micro-osteoperforation: A split-mouth randomized clinical trial. Angle Orthod..

[104] Haddad R., Saadeh M. (2019). Distance to alveolar crestal bone: A critical factor in the success of orthodontic mini-implants. Prog. Orthod..

[105] Azeem M., Saleem M.M., Liaquat A., Ul Haq A., Ul Hamid W., Masood M. (2019). Failure rates of mini-implants inserted in the retromolar area. Int. Orthod..

[106] Çubuk S., Kaya B., Şahinoğlu Z., Ateş U., Özçırpıcı A.A., Uçkan S. (2019). Sagittal skeletal correction using symphyseal miniplate anchorage systems: Success rates and complications. J. Orofac. Orthop..

[107] Ichinohe M., Motoyoshi M., Inaba M., Uchida Y., Kaneko M., Matsuike R., Shimizu N. (2019). Risk factors for failure of orthodontic mini-screws placed in the median palate. J. Oral Sci..

[108] Flieger R., Gedrange T., Grzech-Leśniak K., Dominiak M., Matys J. (2019). Low-Level Laser Therapy with a 635 nm Diode Laser Affects Orthodontic Mini-Implants Stability: A Randomized Clinical Split-Mouth Trial. J. Clin. Med..

[109] Gulduren K., Tumer H., Oz U. (2020). Effects of micro-osteoperforations on intraoral miniscrew anchored maxillary molar distalization: A randomized clinical trial. J. Orofac. Orthop..

[110] Nienkemper M., Willmann J.H., Becker K., Drescher D. (2020). RFA measurements of survival midpalatal orthodontic mini-implants in comparison to initial healing period. Prog. Orthod..

[111] Tseng Y.C., Tsai C.C., Cheng J.H., Chou S.T., Pan C.Y., Chen P.H., Chen C.M. (2020). Recognizing the peak bone mass (age 30) as a cutoff point to achieve the success of orthodontic implants. Odontology.

[112] Melsen B. (2005). Mini-implants: Where are we?. J. Clin. Orthod..

[113] Papadopoulos M.A., Tarawneh F. (2007). The use of miniscrew implants for temporary skeletal anchorage in orthodontics: A comprehensive review. Oral Surg. Oral Med. Oral Pathol. Oral Radiol. Endod..

[114] Munn Z., Peters M.D.J., Stern C., Tufanaru C., McArthur A., Aromataris E. (2018). Systematic review or scoping review? Guidance for authors when choosing between a systematic or scoping review approach. BMC Med. Res. Methodol..

[115] De Clerck H.J., Cornelis M.A., Cevidanes L.L.H., Heymann G.C., Tulloch C.J. (2009). Orthopedic traction of the maxilla with miniplates: A new perspective for treatment of midface deficiency. J. Oral Maxillofac. Surg..

[116] Dalessandri D., Salgarello S., Dalessandri M., Lazzaroni E., Piancino M., Paganelli C., Maiorana C., Santoro F. (2014). Determinants for success rates of temporary anchorage devices in orthodontics: A meta-analysis (*n* > 50). Eur. J. Orthod..

[117] Hong S.B., Kusnoto B., Kim E.J., BeGole E.A., Hwang H.S., Lim H.J. (2016). Prognostic factors associated with the success rates of posterior orthodontic miniscrew implants: A subgroup meta-analysis. Korean J. Orthod..

[118] Alhammadi M.S., Halboub E., Fayed M.S., Labib A., El-Saaidi C. (2018). Global distribution of malocclusion traits: A systematic review. Dent. Press J. Orthod..

[119] Janson G., Barros S.E., de Freitas M.R., Henriques J.F., Pinzan A. (2007). Class II treatment efficiency in maxillary premolar extraction and nonextraction protocols. Am. J. Orthod. Dentofac. Orthop..

[120] Pisek P., Manosudprasit M., Wangsrimongkol T., Keinprasit C., Wongpetch R. (2019). Treatment of a severe Class II Division 1 malocclusion combined with surgical miniscrew anchorage. Am. J. Orthod. Dentofac. Orthop..

[121] Giudice A.L., Rustico L., Longo M., Oteri G., Papadopoulos M.A., Nucera R. (2021). Complications reported with the use of orthodontic miniscrews: A systematic review. Korean J. Orthod..

[122] Kuroda S., Tanaka E. (2014). Risks and complications of miniscrew anchorage in clinical orthodontics. Jpn. Dent. Sci. Rev..

[123] Leo M., Cerroni L., Pasquantonio G., Condò S.G., Condò R. (2016). Temporary anchorage devices (TADs) in orthodontics: Review of the factors that influence the clinical success rate of the mini-implants. Clin. Ter..

