# Supplementary material for: A Scoping Review about the Characteristics and Success-Failure Rates of Temporary Anchorage Devices in Orthodontics
Source: Dent J (Basel). 2022 May 6;10(5):78. doi: 10.3390/dj10050078 (PMC9139455; doi:10.3390/dj10050078)
Supplement: Supplementary file 1 [file dentistry-10-00078-s001.zip › dentistry-1611667-supplementary.pdf]

Table S1. List of cited studies and their publication characteristics (n=103)

Title	Journal	Main Author	Year	Country (First Author)	Objective	Study type	Main Results	Limitations
**Patient's perception on mini-screws used for molar distalization**	Revista Odonto Ciência	Blaya MG [11]	2010	Brazil	To evaluate and compare the perceived pain intensity, side effects and discomfort related to the moment of placement, during mechanics and removal of a mini-screw for molar distalization in orthodontic treatment.	Descriptive study	90% of the patients prefer the utilization of mini-screws rather than premolar extraction (orthodontic camouflage), the use of an extra-oral appliance (Kloehn cervical traction) or another non-compliance treatment (distal jet, jasper jumper or pendulum); for the side effects felt after mini-screw placement, aphthous ulcer was the most frequent followed by gingival inflammation. More than 40% of the patients reported no side effects. The greatest discomfort felt during placement was that of infiltration anesthesia followed by the pressure during mini-screw placement. The majority of the patients reported no pain during mini-screw placement or removal, which may be associated with the degree of satisfaction with the treatment and the willingness to recommend the procedure to a friend. Mini-screws were well accepted by the patients.	N/A
**Comparative study between conventional en-masse retraction (sliding mechanics) and en-masse retraction using orthodontic micro implant**	Implant Dentistry	Basha AG [12]	2010	India	To measure and compare the difference between rate of en-masse retraction with mini-implant and molar anchorage.	Prospective study	Significant amount of anchor loss was noticed in the nonimplant group in the maxillary arch of 1.73 mm. Rate of retraction was statistically and clinically insignificant. No differences in the mean rate of retraction time were noted in both groups.	Only female patients were selected in both groups. There was no cast and cephalometric comparison. Rate of retraction was conducted and anchor loss calculated only for maxillary arch. Axial inclinations of anterior teeth were not matched between 2 groups. Patients missing appointments, debonding of brackets could have contributed to overall outcome of results.
**Effect of smoking on the failure rates of orthodontic miniscrews**	Journal of Orofacial Orthopedics	Bayat E [13]	2010	Germany	To investigate the effect of cigarette smoking on the failure rates of orthodontic miniscrews.	Cohort study	Miniscrews in the heavy smokers exhibited a significantly higher failure rate within the first 4 months after insertion (47.4%) than those in the light smokers (5.6%; $p = 0.008$) or the non-smoker groups (4.1%; $p < 0.001$). No significant intergroup differences	N/A

							became apparent during the subsequent observation period. All the miniscrews rated as failures occurred in conjunction with peri-implant inflammation and loosening of the screw.	
**Cone-beam computed tomography evaluation of mini-implants after placement: Is root proximity a major risk factor for failure?**	American Journal of Orthodontics and Dentofacial Orthopedics	Kim SH [14]	2010	South Korea	To determine factors favoring successful mini-implant placement and to evaluate root proximity as a possible risk factor for failure of osseointegration-based mini-implants during orthodontic treatment.	Descriptive study	The vertical angulation of mini-implant placement has a significantly greater variability than the horizontal angulation. One side root proximity in the osseointegration-based mini-implant and sinus perforations with initial stability might not be major risk factors for mini-implant failure. Several roots in proximity to the mini-implant combined with sinus perforation without initial stability was defined as the major risk factor for screw failure. The amount of root contact area of a mini-implant is more important for its stability.	Additional clinical research with 3D CBCT technology is needed to determine the actual stability of mini-implants after root contact.
**Midpalatal miniscrews for orthodontic anchorage: Factors affecting clinical success**	American Journal of Orthodontics and Dentofacial Orthopedics	Kim YH [15]	2010	South Korea	To investigate the success rate of midpalatal miniscrews used for orthodontic anchorage and the factors affecting clinical success.	Descriptive study	There were no significant associations among success rate and sex, total period of treatment with miniscrews, diameter of miniscrews, types of tooth movements, and variables that represent sagittal and vertical skeletal relationships (ANB, FMA, and Sn-GoGn). The operator’s learning curve, patient’s age, area (midpalatal or parapalatal), and splinting significantly influenced the success rates. After adjusting for other variables, only splinting showed a significant effect on the success rate.	N/A
**Survival analysis of orthodontic mini-implants**	American Journal of Orthodontics and Dentofacial Orthopedics	Lee SJ [16]	2010	South Korea	To investigate the survival characteristics and risk factors of orthodontic mini-implants with survival analyses.	Descriptive study	There was no significant difference in the success rates between implantation sides, clinicians, sex, and oral hygiene. Only the age variable had a significant association with success rate. The mean time of permanence for the implant placed was 156.5 weeks, and the median time of permanence far exceeded the mean orthodontic treatment time.	N/A
**Accurate pre-surgical determination for self-drilling miniscrew implant placement using surgical guides and cone-beam**	European Journal of Orthodontics	Miyazawa K [17]	2010	Japan	To investigate the use of cone-beam computed tomography (CBCT) and precise surgical guides, an approach that provides three-dimensional (3D) control for accurate	Prospective study	Surgical guides can indicate implant inclination and facilitate precise location through the use of CBCT. In blind placement, self-drilling miniscrew implants must be carefully monitored because even guide tubes made on casts often need repositioning between the tooth roots. It is believed	N/A

**computed tomography**					placement of self-drilling miniscrew implants at the desired location and angle.		that the template used in this study for pre-surgical diagnosis, together with the surgical guide, provides the safest means of ensuring accurate implant placement. This approach is particularly valuable when a self-drilling miniscrew implant is inserted by an orthodontist not highly experienced in implant techniques.	
**Relationship between vertical skeletal pattern and success rate of orthodontic mini-implants**	American Journal of Orthodontics and Dentofacial Orthopedics	Moon CH [18]	2010	South Korea	To determine which clinical and skeletal factors are related to the success rate of orthodontic mini-implants in the maxillary and mandibular posterior buccal areas.	Retrospective study	In clinical variables, sex, age, soft-tissue management, sagittal skeletal classification, arch-length discrepancy, and side were not related to the success rate of OMIs, but placement position might be. In the skeletal variables, vertical pattern indicators such as Frankfort-mandibular plane angles and upper gonial angles might be important factors for the success rate of OMIs placed in posterior buccal areas.	N/A
**Factors affecting the long-term stability of orthodontic mini-implants**	American Journal of Orthodontics and Dentofacial Orthopedics	Motoyoshi M [19]	2010	Japan	To determine the placement and removal torques of machine-surfaced mini-implants in patients in relation to placement period, age, sex, and cortical bone thickness to identify factors that affect initial and long-term stability of mini-implants.	Prospective study	Placement and removal torques averaged approximately 8 and 4 N cm respectively, when machinesurfaced mini-implants 1.6 mm in diameter and 8 mm long were placed in the buccal posterior alveolar bone. A torque of 4 N cm should have sufficient anchorage capability for machine-surfaced mini-implants. Placement torque was significantly lower among older patients and with thinner cortical bone in the maxilla, whereas removal torque was not significantly related to placement torque, placement period, age, sex, or cortical bone thickness.	N/A
**Noncompliance screw supported maxillary molar distalization in a parallel manner**	Korean Journal or Orthodontics	Nalçaci R [20]	2010	Turkey	To introduce a screw-supported intraoral distalization appliance and investigate its efficiency.	Prospective study	Upper molars were distalized 3.95 mm on average and a Class I molar relationship was achieved without any anchorage loss during a period of 9 months.	N/A
**A clinical evaluation of orthodontic mini-implants as intraoral anchorage for the intrusion of maxillary anterior teeth**	World Journal of Orthodontics	Saxena S [21]	2010	India	To determine the efficacy of mini-implants as intraoral anchorage during en masse intrusion of the six maxillary anterior teeth.	Prospective study	The amount of intrusion achieved with mini-implants as a rigid source of anchorage for en masse intrusion of the maxillary anterior teeth was statistically and clinically significant. The amount of intrusion achieved amounted to 2.9 ± 1.0 mm for the incisors and 3.5 ± 0.9 mm for the canines. The reason for this difference is probably that the point of force application in this study was closer to the canines than the incisors. The inclination	N/A

							of the incisors hardly changed. The mean duration for intrusion was 4.0 ± 1.5 months; the mean rate of canine intrusion was 0.9 mm per month and that of the incisors was 0.7 mm per month.	
**Clinical study of temporary anchorage devices for orthodontic treatment — Stability of Micro/Mini-screws and Mini-plates: Experience with 455 Cases—**	The Bulletin of Tokyo Dental College	Takaki T [22]	2010	Japan	To determine factors that might cause complications in use of temporary anchorage devices (TADs) for orthodontic anchorage.	Retrospective study	Each type of TAD had a high success rate of over about 90% (94% for mini-plates, 93% for micro-screws, 94% for mini-screws and 89% for palatal screws). The highest failure rate occurred in the mid-palatal region with palatal anchorage systems in young patients, which was the same as that with mini-screws placed in the alveolar region of the mandible. Inflammation rate of soft tissue surrounding orthodontic implants was highest with plate-type implants (acute inflammation), followed by palatal implants and mini-screws. Chronic inflammation mostly occurred with placement of micro-screws in the anterior alveolar region of the maxilla. Both plate- and screw-type orthodontic implants yielded excellent clinical results.	N/A
**Miniscrew implant-supported maxillary canine retraction with and without corticotomy-facilitated orthodontics**	American Journal of Orthodontics and Dentofacial Orthopedics	Aboul-Ela S [23]	2011	Egypt	To clinically evaluate miniscrew implant-supported maxillary canine retraction with corticotomy-facilitated orthodontics (CFO).	Descriptive study	1. CFO can be an effective method for patients who desire shortened orthodontic treatment durations.<br>2. The Luebke–Ochsenbein flap design is a feasible and applicable modification to the original corticotomy flap design.<br>3. Miniscrew implants can function as viable alternatives to conventional molar anchorage. They are simple and efficient anchors for canine retraction, especially in moderate to maximum anchorage situations.	2 patients were excluded from the study—1 because of multiple missed appointments and the other because of poor oral hygiene.
**Assessment of mini-implant displacement using cone beam computed tomography**	Clinical Oral Implants Research	Alves Jr M [24]	2011	Brazil	To assess, through cone beam computed tomography (CBCT), the mini-implants' stability and behaviour when submitted to orthodontic force during upper molars' intrusion.	Descriptive study	Buccal, palatal and midpalatal mini-implants showed some displacement (mean value 0.78) when submitted to force, although they are aimed to provide stable skeletal anchorage.	Six mini-implants were lost, three from buccal sites and three from the midpalatal area. These mini-implants were excluded from the study and three new buccal ones were placed in new sites, whereas transpalatal bars replaced those midpalatal mini-implants.

**Effects of mandibular incisor intrusion obtained using a conventional utility arch vs bone anchorage**	The Angle Orthodontist	Aydogdu E [25]	2011	Turkey	To compare the dentofacial effects of mandibular incisor intrusion using mini-implants with those of a conventional incisor intrusion mechanic, the utility arch.	Clinical trial	Incisor intrusion that was achieved using an implant-supported segmented archwire was no different than the movement achieved with a conventional intrusion utility arch. In the implant group, the mean amount of change was 0.4 mm/mo for the incisor tip and 0.3 mm/mo for the center of resistance, and in the utility arch group, the mean amount of change was 0.25 mm/mo for the incisor tip and 0.2 mm/mo for the center of resistance. The mandibular incisors showed an average protrusion of 7° in the implant group and 8° in the utility arch group.	N/A
**Orthopedic correction of growing hyperdivergent, retrognathic patients with miniscrew implants**	Journal of Oral and Maxillofacial Surgery	Buschang PH [26]	2011	United States	To evaluate the skeletal and dental effects of intruding segments of teeth in a controlled fashion using miniscrew implants.	Prospective study	The chin was advanced by a mean of 2.4 mm, the sella-nasion-basion (SNB) angle increased by 2.1°, the mandibular plane angle decreased by 3.9°, and facial convexity decreased by approximately 3.2°. The treatment approach was not painful or uncomfortable; the majority of the patients indicated that they were very likely to recommend the treatment to others.	N/A
**Comparison of movement of the upper dentition according to anchorage method: Orthodontic mini-implant versus conventional anchorage reinforcement in Class I malocclusion**	International Scholarly Research Network	Lee AY [27]	2011	South Korea	To compare the amounts of anchorage loss in the upper first molar and of retraction of the upper central incisor in cases with Class I malocclusion between orthodontic mini-implants and conventional anchorage reinforcements.	Retrospective study	Although the orthodontic mini-implants (OMI) could not reduce the treatment duration, they provided better maximumposterior anchorage and greater retraction of the upper anterior teeth than conventional anchorage reinforcements (CAR) in spite of hyperdivergent pattern. Therefore, the null hypothesis was rejected. In addition, OMI led to an intrusion of the upper central incisor and first molar, whereas CAR resulted in extrusion of these teeth.	N/A
**Displacement pattern of the maxillary arch depending on miniscrew position in sliding mechanics**	American Journal of Orthodontics and Dentofacial Orthopedics	Lee KJ [28]	2011	South Korea	To evaluate the anteroposterior and vertical displacement patterns of the maxillary teeth in sliding mechanics depending on the position of interradicular miniscrews after the extraction of premolars.	Retrospective study	1. Both groups had sufficient anchorage reinforcement for maximum anterior retraction amounts of 7.20 mm in group A and 7.32 mm in group B. The ANB angle was significantly decreased in both groups, with no group difference.<br>2. In group A, the vertical position of the incisal edge did not change significantly during the retraction period. In group B, significantly greater intrusion (1.59 mm) was found compared with group A.<br>3. Neither group displayed significant	4 miniscrews failed during retraction (2 in group A, 2 in group B). In the failure cases, retraction was transitionally discontinued for 2 to 3 months, and the miniscrews were replaced in the same interradicular area, with some clearance from the original site. None of the replaced miniscrews

							changes in skeletal vertical dimensions. According to the results, simultaneous intrusion and retraction can be effectively obtained by using miniscrews between the premolars in extraction patients, without any intervention of the intrusive mechanics.	failed; hence, the failures were considered to have little influence on the results.
**Expectations, acceptance and preferences of patients in treatment with orthodontic mini-implants. Part II: Implant removal**	Journal of Orofacial Orthopedics	Lehnen S [29]	2011	Germany	To compare mechanical and manual techniques to remove orthodontic mini-implants. We also investigated whether the treated patients preferred local anesthetics. The study's focus is on the patients' perceptions and the resulting preferences.	Descriptive study	No significant differences between the two groups with respect to the intensity of their symptoms. However, the noise associated with the handpiece was found to be unpleasant and tended to lead to more symptoms than when no handpiece was used. Pain perceived during mini-implant removal was relatively slight and not much affected by the use of local anesthetics. The most severe symptoms were associated with the injection itself. The non-injected side experienced significantly less discomfort and was thus the preferred side in both groups.	N/A
**Predictors of initial stability of orthodontic miniscrew implants**	European Journal of Orthodontics	Lim HJ [30]	2011	South Korea	To elucidate potential confounding factors affecting initial stability of miniscrews inserted to enhance orthodontic anchorage.	Retrospective study	The screws inserted by more experienced clinicians (more than 20 miniscrews) were found to have approximately a 3.6-fold higher success rate of initial stability compared with those inserted by less experienced clinicians after adjusting for the insertion site (aOR = 3.63, P = 0.015). The results of the present study suggest that the initial stability depends on insertion site and clinician experience.	N/A
**Factors influencing the stability of miniscrews. A retrospective study on 300 miniscrews**	European Journal of Orthodontics	Manni A [31]	2011	Italy	To investigate, over a period of approximately 3 years, the reactions to orthodontic loading of type V titanium screws with an untreated surface.	Retrospective study	The success rate is better in male patients. 1.3 mm wide 11 mm length miniscrew has a better success rate. Most favourable insertion in the attached gingiva followed by insertion in the mucogingival line. Loading not exceeding 150–250 g should be immediately applied to the screw.	N/A
**Treatment effects of microimplant-aided sliding mechanics on distal retraction of posterior teeth**	American Journal of Orthodontics and Dentofacial Orthopedics	Oh YH [32]	2011	South Korea	To quantify the treatment effects of microimplant-aided mechanics on group distal retraction of the posterior teeth.	Retrospective study	The upper and lower lips were repositioned distally. The Frankfort horizontal to mandibular plane angle was decreased in the adult group. Themaxillary posterior teeth were distalized by 1.4 to 2.0mmwith approximately 3.5 of distal tipping, and the mandibular posterior teeth were also distalized by 1.6 to 2.5 mm	N/A

							with approximately 6.6 to 8.3 of distal tipping. The maxillary posterior teeth showed intrusion by 1 mm. There were increases in arch widths at the premolars and molars. The overall success of microimplants was 89.7%. The overall success rate of the microimplants was 89.7%. The success rates were 98.1% for a well-experienced clinician and 70.8% for postgraduate students. Many factors affect the success of microimplants. The success seems to be influenced by the operator‘s skill.	
**Success rate of microimplants in a University orthodontic clinic**	International Scholarly Research Network	Sharma P [33]	2011	India	To find factors related to the clinical success of micro-implants in Asian patients.	Retrospective study	The clinical variables of microimplant factors (type), patient factors (sex, skeletal and dental relationships, overbite, jaw involved, side involved and site involved), and treatment factors (type of insertion, time of loading, purpose of microimplant insertion, mode of loading, type of anchorage used, direction of forces applied) did not show any statistical difference in success rates. Mandibular angle, vertical position of implant placement, oral hygiene status, and inflammation showed significant difference in success rates.	N/A
**Placement and removal torque values of orthodontic miniscrew implants**	American Journal of Orthodontics and Dentofacial Orthopedics	Suzuki EY [34]	2011	Thailand	To analyze the placement and removal torque values of orthodontic miniscrew implants in several sites in the maxilla and mandible.	Descriptive study	The maximum insertion torque (MIT) values were significantly higher for the self-drilling miniscrews (mean, 14.5 Ncm) than for the pre-drilling miniscrews (mean, 9.2 Ncm) in all implant sites. For both pre-drilling and self-drilling miniscrews, the highest MIT values were observed at the midpalatal suture site followed by the dentoalveolar bones of the mandible and maxilla, respectively. In contrast, maximum removal torque (MRT) values were significantly higher for the pre-drilling miniscrews (mean, 22.6 Ncm) than for the self-drilling miniscrews (mean, 17.6 Ncm). Accordingly, the mean torque ratio of predrilling miniscrews was twice that of the self-drilling miniscrews.	Although the torque values could be easily assessed clinically during both placement and removal procedures, it was not possible to quantify the amount of bone-screw contact, and therefore, it was not possible to define whether partial or total osseointegration had occurred surrounding these miniscrew implants. Moreover, the torque assessment method does not allow monitoring the stability of miniscrew implants throughout the application of orthodontic loading.

**The effect of drill-free and drilling methods on the stability of mini-implants under early orthodontic loading in adolescent patients**	European Journal of Orthodontics	Türköz Ç [35]	2011	Turkey	To evaluate the early stability of mini-implants using drill-free and drilling methods both before and after orthodontic force application.	Retrospective study	Mini-implants inserted using the drill-free method provide the highest success rate before orthodontic force application and also maintain their stability during a period of 1 month after early force loading. Smaller drill diameters can contribute to clinical stability of mini-implants in the short-term, however, evaluations are needed to clarify long-term stability.	N/A
**Alveolar bone density change around miniscrews: A prospective clinical study**	American Journal of Orthodontics and Dentofacial Orthopedics	Al Maaitah EF [36]	2012	Jordan	To determine in a prospective way whether interproximal alveolar bone density will change after insertion of miniscrews upon loading and to observe the change in the gingival tissues around the miniscrews during orthodontic treatment.	Clinical trial	Male patients have a higher alveolar bone density than female patients. Maxillary alveolar bone density increased significantly around miniscrews after 3 months of insertion. Miniscrews were not found to have any detrimental effects on the adjacent gingival-tissue health. Fixed orthodontic appliance treatment and not the miniscrews increased the width of keratinized gingiva significantly 1 month after treatment. Fixed orthodontic appliance treatment did not affect alveolar bone density significantly in the maxilla. Careful insertion of miniscrews has no harmful effect on the vitality of the adjacent teeth.	N/A
**Dentofacial effects of two facemask therapies for maxillary protraction - Miniscrew implants versus rapid maxillary expanders**	The Angle Orthodontist	Ge YS [37]	2012	China	To evaluate the effects of maxillary protraction using miniscrew implants with facemask (MSI/FM). Specifically, we compared the active treatment effects of skeletal, dentoalveolar, and soft tissue structures in the MSI/FM group with changes in a matched control group treated by using rapid maxillary expanders in conjunction with facemask (RME/FM).	Clinical trial	Miniscrews can be used as a reliable means of rigid anchorage for maxillary protraction. The zygomatic buttress of the maxilla is a substantial region where skeletal anchorage can be placed. Compared with the RME/FM protocol, the MSI/FM therapy produces a similar maxillary advancement and mandibular restraining in Class III patients with maxillary deficiency using a smaller magnitude of protraction force. The MSI/FM protocol improves skeletal relationships and soft tissue profile and eliminates the undesired proclination of the maxillary incisors and reduces the mesialization of the maxillary dentition, which are present in the RME/FM therapy.	During treatment, four subjects of the initial MSI/FM group were excluded because of the mobility of miniscrews, and one subject of each group was excluded because of poor cooperation.
**A comparitive clinical study between self tapping and drill free screws as a source of rigid**	Journal of Oral and Maxillofacial Surgery	Gupta N [38]	2012	India	To evaluate and compare the stability and clinical response of soft tissue around the self tapping and drill free screws when used	Clinical trial	It can be concluded that both self-tapping and the drill-free screws are effective anchorage units. However, the latter have an edge over the conventional self-tapping screws because of decrease in operative time,	N/A

**orthodontic anchorage**					as anchorage unit for en-masse retraction of maxillary anterior teeth.		little bone debris, less thermal damage, lower morbidity and minimal patient discomfort as predrilling is not required, thus they can be used as viable alternative to self-tapping screws. However, self-tapping screws are still recommended for areas with high bone density and thick cortical bone where drill-free screws are not useful.	
**Prognostic parameters contributing to palatal implant failures: A long-term survival analysis of 239 patients**	Clinical Oral Implants Research	Jung BA [39]	2012	Germany	To evaluate the demographic (age and gender), radiological (vertical bone height along the prospective implant axis) and therapeutic (design of the supraconstruction, direction of loading, type of anchorage, magnitude of orthodontic forces) parameters that might influence the survival of palatal implants.	Retrospective study	Palatal implants proved highly successful (survival rate 495%) in a large cohort of patients. The treatment concept appears to be robust with respect to the variability of parameters such as age, gender, type of suprastructure and magnitude or direction of orthodontic forces. However, palatal implants are associated with a vulnerable period in the healing phase. The surgeon's experience markedly influenced the success of palatal implants; the learning curve for palatal implants appears to be implant-specific.	N/A
**Comparison between Herbst appliances with or without miniscrew anchorage**	Dental Research Journal	Manni A [40]	2012	Italy	To analyze dental and skeletal effects of an acrylic Herbst - miniscrews combined device in comparison to acrylic cast splints Herbst appliance, in the correction of Class II malocclusion.	Retrospective study	The miniscrew Herbst system allows correction of Class II malocclusion, with a slight lower incisor proclination during treatment.	N/A
**Root proximity and cortical bone thickness effects on the success rate of orthodontic micro-implants using cone beam computed tomography**	The Angle Orthodontist	Min KI [41]	2012	South Korea	To evaluate factors (root proximity and cortical bone thickness) affecting the success rate of orthodontic micro-implants (OMIs) using cone-beam computed tomography (CBCT) images.	Descriptive study	The success rate increased as the distance between the root surface and OMI increased, showing a highly significant statistical correlation ($P > 0.05$). As the cortical bone thickness increased, the success rate increased, showing a slight, nonsignificant correlation ($P > 0.05$). Thus, the success rate of OMIs was affected more significantly by root proximity than cortical bone thickness.	N/A
**Bone density and miniscrew stability in orthodontic patients**	Australian Orthodontic Journal	Samrit V [42]	2012	India	To evaluate bone density in buccal inter-radicular bone between second premolars and first permanent molars and its association with the clinical stability of miniscrews used for en-	Descriptive study	Bone density values were not related to miniscrew stability when indirectly loading the miniscrews with a continuous force of 2N after the first week following placement. An association between miniscrew success and jaw bone density could not be established.	N/A

					masse retraction of anterior teeth in extraction cases.			
**Treatment effects of intrusion arches and mini-implant systems in deepbite patients**	American Journal of Orthodontics and Dentofacial Orthopedics	Senisik NE [43]	2012	Turkey	To compare the 2 maxillary intrusion systems involving mini-implants and Connecticut intrusion arches used as intraoral intrusion systems.	Clinical trial	The maxillary incisor intrusion rates of the Connecticut intrusion arch and the mini-implant systems were similar. Loss of sagittal and vertical anchorage during intrusion in the Connecticut intrusion arch group, but not in the implant group.	N/A
**Clinical factors correlated with the success rate of miniscrews in orthodontic treatment**	International Journal of Oral Science	Topouzelis N [44]	2012	Greece	To assess the success rate of miniscrews and to correlate indicators that may be affecting their success, namely, the indicators that may have an impact on the stability of miniscrews during the time period required for orthodontic treatment.	Retrospective study	The success rate of miniscrews in this study was 90.2%. The success rate per miniscrew decreased significantly as the number of miniscrews used per patient increased. Retromandibular triangle and palatal placement of miniscrews resulted in lower success rates as compared to buccal placement. Miniscrew placement in attached gingiva showed higher success rates than placement in movable mucosa. Furthermore, the variables of miniscrew length, miniscrew diameter, surgical placement procedure (flapped or flapless) and orthodontic force applied on the miniscrew presented significant correlation with success rates.	N/A
**Mini-implants vs fixed functional appliances for treatment of young adult Class II female patients A prospective clinical trial**	The Angle Orthodontist	Upadhyay M [45]	2012	United States	To examine the dentoskeletal and soft tissue treatment effects of maxillary anterior tooth retraction with mini-implant anchorage in Class II division I patients undergoing extraction of only the maxillary first premolars in comparison to similar patients undergoing treatment with a nonextraction approach using a fixed functional appliance (FFA).	Clinical trial	The two treatment protocols provided adequate dental compensation for the Class II malocclusion, but did not affect the skeletal discrepancy. There were significant differences in the dental and soft tissue treatment effects between the groups. In particular, the lower incisors showed significant flaring in the fixed functional appliance group. The treatment time was significantly less with FFAs.	Two patients did not agree to participate and were, therefore, not included.
**Mini-implants in the palatal slope. A retrospective analysis of implant survival and tissue reaction**	Head & Face Medicine	Ziebura T [46]	2012	Germany	To identify insertion procedure and force application-related complications in Jet Screw (JS) type mini-implants when inserted in the palatal slope.	Retrospective study	The JS mini-implant is reliable for sagittal and vertical movements or anchorage purposes. Laterally directed forces might be unfavorable. The selection o implant length, as well as the insertion procedure, should account for the possibility of gingival overgrowth.	N/A

**A prospective comparative study between differential moments and miniscrews in anchorage control**	European Journal of Orthodontics	Davoody AR [47]	2012	United States	To compare and analyse the amount of anchorage loss during space closure by anterior retraction following first premolar extraction.	Clinical trial	Both treatment methods, differential moments and miniscrews, are effective ways of controlling anchorage in cases requiring premolar extraction with anterior teeth retraction. There is statistically and clinically significant anchorage loss when differential moments are used compared with TADs. Also, the type of treatment is dependent on the requirement of each individual case based on its diagnosis and treatment plan. Moreover, there is greater anterior torque control when differential moments are used compared with TADs. Additionally, there is no difference in the amount of lip retraction when TADs are used compared with differential moments. Both anchorage modalities show statistically significant retraction of the lips during treatment.	Miniscrews that failed were replaced at the next visit. Patients that presented with failed miniscrews twice were excluded from the study.
**Influence of miniscrew dental root proximity on its degree of late stability**	International Journal of Oral & Maxillofacial Surgery	Janson G [48]	2012	Brazil	To compare the stability and success rate of selfdrilling miniscrews inserted into the interradicular septum with critical and non-critical dimensions, and to evaluate the influence of miniscrew dental root proximity on the degree of stability of these anchorage devices.	Descriptive study	The septum width was not a critical factor for mini-implant stability, but an extreme degree of root proximity, indicating PDL invasion could increase mini-implant failure. Mini-implant success rate and degree of mobility were not significantly affected by the presence of movable mucosa even in association with a measurable amount of bacterial plaque. Patient sensitivity was frequently associated with some degree of mini-implant mobility, which progressed to mini-implant loss in a short time after insertion.	N/A
**Distalization pattern of the maxillary arch depending on the number of orthodontic miniscrews**	The Angle Orthodontist	Bechtold TE [49]	2013	South Korea	To investigate how single or dual interradicular miniscrews with respective linear force vectors would affect the amount and pattern of distalization of the maxillary arch in the Class II patients.	Clinical trial	Significant distalization in the molars and incisors was shown in both groups. Significantly greater distalization and intrusion of the first molar and intrusive displacement of the incisor, together with significant reduction of the mandibular plane, were noted in group B, in contrast to the rotation of the occlusal plane in group A.	N/A
**Placement angle effects on the success rate of orthodontic microimplants and other factors with cone-beam**	American Journal of Orthodontics and Dentofacial Orthopedics	Jung YR [50]	2013	South Korea	To evaluate the effect of placement angles on the success rate of orthodontic microimplants and other factors with cone-beam computed tomography images.	Descriptive study	The success rates were higher on the left side than on the right side, in adults than in younger patients, and in female subjects than in male subjects, although these trends were not statistically significant. The success rate of orthodontic microimplants is not affected by placement angles and is	N/A

**computed tomography**							more significantly affected by root proximity than by cortical bone thickness. Cortical bone thickness is affected by placement angles, but root proximity is not affected by placement angles.	
**Maxillary protraction using a hybrid hyrax-facemask combination**	Progress in Orthodontics	Nienkemper M [51]	2013	Germany	To evaluate the treatment effects produced by the hybrid hyrax-face mask combination in growing class III patients.	Descriptive study	The hybrid hyrax–facemask combination seems to be effective for orthopaedic treatment in growing class III patients. Significant sagittal improvement of the maxilla and inhibition of the mandible can be achieved. Unwanted maxillary dental movements can be avoided due to stable skeletal anchorage. The surgical invasiveness is comparatively low.	N/A
**Root proximity and inclination of orthodontic mini-implants after placement: Cone-beam computed tomography evaluation**	American Journal of Orthodontics and Dentofacial Orthopedics	Shinohara A [52]	2013	Japan	To investigate root proximity and variability of the placement inclination of a mini-implant according to placement position.	Descriptive study	Of 147 implants, approximately 20% were in contact with a root. The root contact was related to the failure of the mini-implants.	N/A
**Evaluation of optimal length and insertion torque for miniscrews**	American Journal of Orthodontics and Dentofacial Orthopedics	Suzuki M [53]	2013	Japan	To test the theory that short miniscrews will decrease the possibility of damaging the root, but the failure rate will increase.	Descriptive study	The optimum lengths of miniscrews of a diameter of 1.3 mm are 5 mm in the maxilla and 6 mm in the mandible. The cutoff point for minimum bone necessary for miniscrew stabilization is approximately 3.8 mm. Root proximity rather than bone density is the major factor in miniscrew failure. The ideal insertion torque was 5N to 10N.	N/A
**Orthodontic miniscrew failure rate and root proximity, insertion angle, bone contact length, and bone density**	Orthodontics & Craniofacial Research	Watanabe H [54]	2013	Japan	To test the hypothesis that there is no significant correlation between miniscrew failure rate and root proximity, insertion angle, bone contact length, and bone density.	Descriptive study	This investigation has revealed that combining a simple stent with CBCT is useful for determining the location and angle of miniscrews during their placement. A higher failure rate occurred for the miniscrews placed in the mandible than for those placed in the maxilla. Our major finding was that root proximity is the factor that most affected miniscrew failure, especially for miniscrews placed in the mandible. Bone density was not a major determinant of miniscrew failure, and neither bone contact length nor miniscrew angle had a major effect on miniscrew failure. In addition, correction of the X-ray attenuation coefficient is necessary for measuring bone density using CBCT. We suggest that there are limitations on the use of	N/A

							periapical dental x-rays, and we recommend CBCT for evaluating the root proximity of miniscrews.	
**Zygomatic mini-implant for Class II correction in growing patients**	Journal of Orofacial Orthopedics	El-Dawlatly M [55]	2014	Egypt	To evaluate the possible skeletal and dental effects of zygomatic mini-implants for the correction of Class II malocclusions in growing female subjects.	Cohort study	Significant retrusion of point A, anti-clockwise rotation of the maxillary plane, and a mean molar distalization of 2.92 ± 0.69 mm with no extrusion, no tipping or buccal rolling. There was significant upper incisor intrusion (1.89 ± 0.84 mm) with no changes in incisor inclination. No change in the mandibular plane angle was detected.	N/A
**Factors affecting the clinical success of orthodontic anchorage: Experience with 266 temporary anchorage devices**	Journal of Dental Sciences	Lai TT [56]	2014	Taiwan	To evaluate the failure rates and to analyze potential factors associated with the stability of TADs used for orthodontic treatment.	Retrospective study	We investigated five categories and found that all implant-related factors (diameter and length), all patient-related factors (sex, age, and type of malocclusion), and one location-related factor (side) did not result in statistically significant differences in the success rates of TADs. We also found a reduced success rate in the following categories: location-related factors (jaw (mandible), site (lingual), bone quality (Q4), and the type of soft tissue around TADs (mucosa)), orthodontic-related factors (timing of force application equal to 2 weeks), and implant-maintenance factors (local inflammation (mild moderate and moderate severe)). We also suggested that in order to improve the success rates, local inflammation should be monitored and controlled, force application should be 4 weeks after insertion, and the location for TAD placement should be good quality bone with keratinized mucosa.	N/A
**Assessment of damping capacity as an index of root proximity in self-drilling orthodontic mini-implants**	Clinical Oral Investigations	Motoyoshi M [57]	2014	Japan	To investigate orthodontic mini-implant root proximity, placement torque, and damping capacity and to determine whether placement torque and damping capacity (Periotest value (PTV)) are useful indices for the estimation of mini-implant root proximity.	Descriptive study	Placement torque did not differ significantly according to root proximity. Placement torque could not be used to estimate root proximity. Success rates for maxillary mini-implants were high in the self-drilling and self-tapping groups in this study.	N/A
**Effectiveness of 3 methods of anchorage reinforcement for**	American Journal of Orthodontics	Sandler J [58]	2014	United Kingdom	To test the hypothesis that there is no difference in the effects of TADs, headgear, and	Clinical trial	Conclude the following: (1) there was no difference in the effectiveness of TADs, Nance button palatal arches, and headgear for reinforcing anchorage	N/A

**maximum anchorage in adolescents: A 3-arm multicenter randomized clinical trial**	and Dentofacial Orthopedics				Nance button palatal arches when used to reinforce orthodontic anchorage with respect to (1) the amount of molar tooth movement, (2) the duration of treatment, (3) the number of treatment visits, (4) the total treatment time, (5) dento-occlusal changes (peer assessment rating [PAR] index), and (6) the patients' perceptions of the treatment.		during orthodontic treatment; and (2) the information from this study can be used to help orthodontists and patients determine their preferences for the method of anchorage reinforcement.	
**Root proximity and stability of orthodontic anchor screws**	Journal of Oral Science	Shigeeda T [59]	2014	Japan	To investigate a causal relationship between the stability of orthodontic anchor screws and the degree of their proximity to the root using mobility test device and cone-beam computed tomography.	Descriptive study	The failure rate of screws with contact and without contact significantly differed in the mandible. Even in the absence of root contact, mandibular screws had greater mobility than maxillar screws. The lower stability of mandibular screws with root contact might be related to their greater mobility.	N/A
**Comparative study of the primary stability of self-drilling and self tapping orthodontic miniscrews**	American Journal of Orthodontics and Dentofacial Orthopedics	Son S [60]	2014	Japan	To identify the influences on miniscrew stability of the self-tapping and self-drilling placement techniques.	Clinical trial	1. We do not recommend the self-tapping method over the self-drilling method in maxillary alveolar bone because both placement techniques had high success rates.<br>2. The self-drilling miniscrews showed greater mobility than did the self-tapping miniscrews, although this difference did not influence the success rate of the self-drilling method.<br>3. Special attention to root proximity is recommended because miniscrews with root contact had significantly greater mobility when placed with the self-drilling method compared with the self-tapping method.<br>4. With self-tapping miniscrews, root contact can be overlooked because it did not affect the high mobility in the self-tapping group.	N/A
**A comparison of tapered and cylindrical miniscrew stability**	European Journal of Orthodontics	Yoo SH [61]	2014	South Korea	To evaluate the clinical efficiency of tapered and cylindrical miniscrews by estimating their success rate and long-term stability, while also evaluating the	Prospective study	The long-term stability and success rates of tapered and cylindrical miniscrews were similar and there was no significant difference. The distal area of the first molar had significantly lower success rates than other sites. Stability and success rate of miniscrews can be affected by various	N/A

					relationship between initial and longterm stability.		factors such as insertion torque, individual anatomical variation, and insertion site.	
**Effectiveness of mini implant in three-dimensional control during retraction - A clinical study**	Journal of Clinical and Diagnostic Research	Victor D [62]	2014	India	To determine and compare, the torque of incisors, tip of molars and vertical control during the orthodontic treatment, using MBT appliance system with and without mini screw implants.	Clinical trial	The implant provides excellent three-dimensional control of the anterior and posterior segment during retraction when compared to the conventional mode of anchorage. The following advantages are cited, 1. The torque control between the Groups is comparable. 2. The tip control of molar is better in the implant Group. 3. True intrusion is possible with the implant Group. 4. Vertical control of the molar is better in the implant Group.	N/A
**Anchorage loss due to Herbst mechanics—preventable through miniscrews?**	European Journal of Orthodontics	Bremen J [63]	2014	Germany	To assess whether mandibular anchorage loss during treatment with Herbst/multibracket (MB) appliances can be prevented, using inter-radicular MI anchorage.	Descriptive study	MI anchorage as used in this study did reduce proclination of the lower labial segment to a small extent, but could not prevent it reliably. The differences in anchorage preservation between the study and control groups, although statistically significant, were small and were unlikely to be of clinical relevance. The relatively small anchorage benefits of MI anchorage, the large interindividual variation as well as the high loss rate do not appear to justify their additional expense. Extrapolating from the data of this study, we cannot recommend routine use of MI in conjunction with Herbst appliance treatment to prevent proclination and/or protrusion of the lower incisors.	N/A
**A new method to evaluate the positional stability of a self-drilling miniscrew**	Orthodontics & Craniofacial Research	Chen G [3]	2015	China	To evaluate the positional stability of miniscrews during orthodontic treatment change in cone-beam computed tomography.	Descriptive study	Both the unloaded and loaded miniscrews used in this study showed positional stability during en-masse retraction in adults.	N/A
**Effects of tooth root contact on the stability of orthodontic anchor screws in the maxilla: Comparison between self-drilling and self-tapping methods**	American Journal of Orthodontics and Dentofacial Orthopedics	Iwai H [64]	2015	Japan	To create evidence for clinicians regarding selection of placement methods for orthodontic anchor screws.	Clinical trial	1. The success rate of the screws showed no significant difference between the self-drilling and self-tapping methods in the maxilla. 2. Self-drilling screws were placed more horizontally in terms of vertical screw placement angle. Thus, the screws tended to be placed at a site proximal to the tooth root in the self-drilling method more than in the self-tapping method, but the incidence of	N/A

							tooth root contact showed no significant difference between the methods.<br>3. Self-drilling screws that contacted the tooth root showed a significantly higher failure rate because their stability was affected more by tooth root contact than were the self-tapping screws.	
**Analysis of time to failure of orthodontic mini-implants after insertion or loading**	Journal of the Korean Association of Oral and Maxillofacial Surgeons	Jeong JW [65]	2015	South Korea	To evaluate patterns of failure time after insertion, failure rate according to loading time after insertion, and the patterns of failure after loading.	Prospective study	Approximately 75% of mini-implant failures occurred within 16 weeks of insertion. When the loading time after insertion was less than 12 weeks, the failure rate of the mini-implant was high. The failure rate according to loading time after insertion was highest when the mini-implants were loaded during the first week after insertion. Immediate loading could cause failure of a mini-implant.	N/A
**Three-dimensional analysis of the distal movement of maxillary first molars in patients fitted with mini-implant-aided transpalatal arches**	Korean Journal or Orthodontics	Miresmaeilia A [66]	2015	Iran	To measure the three-dimensional (3D) movement of first molar after using this newly designed distalizing appliance.	Clinical trial	The MIA-TPA is an appliance that can achieve absolute anchorage, and successfully drive maxillary first molars distally. This movement is concomitant with expansion. Due to the apical direction of distal force, extrusion can be prevented and molar movement is relatively slow.	N/A
**Maxillary sinus perforation by orthodontic anchor screws**	Journal of Oral Science	Motoyoshi M [67]	2015	Japan	To investigate the frequency of miniscrew perforation of the maxillary sinus and the effects of sinus perforation on screw stability. In addition, we discuss the relationships between sinus perforation, mucosal thickening when placing miniscrews, miniscrew stability, and the frequency of onset of maxillary sinusitis after screw placement.	Descriptive study	Approximately 10% of miniscrews perforated the maxillary sinus, but maxillary sinus perforations ≤1.5 mm in depth are unlikely to affect screw stability. Small, uncomplicated perforations of the maxillary sinus by miniscrews may heal spontaneously. To avoid maxillary sinus perforation, the thickness of the sinus floor should be >6.0 mm o the screw length should be <6 mm.	N/A
**Effect of the length of orthodontic mini-screw implants on their long-term stability: A prospective study**	The Angle Orthodontist	Sarul M [68]	2015	Poland	To analyze the influence of one factor only—the length of TISAD/TAD—on the long-term stability of TISAD/TAD in the mandible and in a homogenous group of	Prospective study	Eight-millimeter orthodontic mini-screw implants inserted in the mandibles of 20- to 29-year-old women, loaded 2 weeks after insertion with a continuous force ranging from 100 to 150 g, are significantly more stable than the 6-mm implants.	N/A

					patients to minimize the fortuity of the results.			
**Failure rates of mini-implants placed in the infrazygomatic region**	Progress in Orthodontics	Uribe F [5]	2015	Colombia - USA	To evaluate failure rates of mini-implants placed in the IZ crest of the maxilla and investigate the factors affecting this unfavorable outcome.	Cohort study	Failure rates were higher among those aged ≥18 years, males, with medical conditions, use of non-Lomas implants, implants with length of 6 to 8 mm (compared to 9 mm), implants with 1.5/1.8 mm diameter (compared to 2 or 2.3 mm diameter), use of force greater than 150 g, with poor oral hygiene, when placed by inexperienced operators, and left-sided implants. Failure rates were lower when pilot holes were used. Patient, mini implant, orthodontic, surgical, and mini-implant maintenance factors were not predictive of failure rates.	N/A
**A study of success rate of miniscrew implants as temporary anchorage devices in Singapore**	International Journal of Dentistry	Yi Lin S [69]	2015	Singapore	To find out the success rate of miniscrew implants in NDCS pertaining to our local population, and whether they are a reliable form of TAD. Secondary objectives of this research will include finding out if patient-related factors, location-related factors, and miniscrew implant-related factors have any impact on success rates.	Retrospective study	The overall success rate is 83.3% after 12 months. Patient-related factors like vertical skeletal malocclusion were found to influence success: average mandibular plane angle patients have a higher chance of success compared to high mandibular angle patients probably due to the less dense cortical bone of the latter. Miniscrew implant location-related factors have no significant effect on success but careful site selection must still be done to avoid encroaching on vital structures and to optimize orthodontic mechanics. Of the miniscrew implant-related factors, only length of miniscrew implant was significantly correlated with success. Thus, as long as surrounding anatomy permits, a longer miniscrew implant for better mechanical retention is recommended for higher success rate.	Due to the retrospective nature of this study, datum was sometimes lacking and not every variable mentioned in the literature was investigated and confounding factors may be present.
**Comparison of short-term effects between face mask and skeletal anchorage therapy with intermaxillary elastics in patients with maxillary retrognathia**	European Journal of Orthodontics	Aglarci C [70]	2016	Turkey	To compare the short-term dental and skeletal effects of a face mask (FM) with those of skeletal anchorage (SA) therapy with intermaxillary elastics in prepubertal patients with skeletal Class III malocclusion.	Prospective study	Oral hygiene and cortical bone density are the most important factors affecting the stability of mini-implants. Patients treated with mini-implants and mini-plates exhibited skeletal improvements, with little effect on mandibular position.	N/A
**Comparison of anterior and posterior mini-implant assisted**	The Angle Orthodontist	Aras I [71]	2016	Turkey	To compare, by means of CBCT, the amount of root resorption and treatment efficacy	Clinical trial	The four maxillary incisors can be effectively intruded on sectional archwires with forces of 40 g per side from anteriorly or posteriorly located	31 patients were included in the final assessment due to the

**maxillary incisor intrusion: Root resorption and treatment efficiency**					resulting from incisor intrusion supported by anterior vs posterior mini-implants.		mini-implants. The rates of both intrusion and root resorption were higher using the anteriorly placed, mini-implant-supported incisor intrusion method compared with intrusion rates resulting from the posteriorly placed mini-implants. In patients demonstrating upright incisors, intrusion anchored from posterior mini-implants yielded more labial flaring and less root resorption than that anchored anteriorly. Since both incisor intrusion and distalization are possible with mechanics anchoring from posterior mini-implants, usage of mini-implants in this manner presents an alternative to anterior mini-implants in deep-bite cases with premolar extraction. Further studies need to be conducted to observe the pros and cons of this approach.	loss of stability in one anterior mini-implant.
**Three-dimensional analysis of tooth movements after palatal miniscrew-supported molar distalization**	American Journal of Orthodontics and Dentofacial Orthopedics	Duran GS [72]	2016	Turkey	To evaluate the dentoalveolar effects of a palatal miniscrew-supported molar distalization appliance using a 3-dimensional reverse engineering method.	Clinical trial	Through support from the anterior palatal region, the maxillary first molars were distalized without anchorage loss. Furthermore, movement was observed in all 3 planes of space with reduction from the posterior to the anterior in the maxillary arch.	N/A
**Three-dimensional effects of the mini-implant–anchored Forsus Fatigue Resistant Device: A randomized controlled trial**	The Angle Orthodontist	Elkordy SA [73]	2016	Egypt	To detect the 3D dental and skeletal changes associated with the use of indirect mini-implant anchorage with Forsus Fatigue Resistant Device (FFRD) and to test the efficiency of this type of anchorage in obtaining skeletal rather than dentoalveolar effects.	Clinical trial	FFRD was successful in treatment of Class II division 1 malocclusion through dentoalveolar changes and minimal significant skeletal changes. The use of mini-implants with FFRD could not produce significant additional sagittal skeletal effects. The incorporation of mini-implants with FFRD decreased the mandibular dentoalveolar side effects and increased the distalizing effects of the appliance on the maxillary arch.	N/A
**Comparison of anchorage pattern under two types of orthodontic mini-implant loading during retraction in type a anchorage cases**	Journal of Clinical and Diagnostic Research	Khan BI [74]	2016	India	To directly determine and compare the reciprocal displacement of orthodontic mini-implant under two types of loading protocols during orthodontic retraction.	Clinical trial	This study demonstrated that Orthodontic mini-implants loading after a waiting period of two weeks has a mechanical advantage over the immediate loading implants. The delayed loading is beneficial as compared to the immediate loading in terms of space closure for Type A anchorage cases. The head and tail on the immediate loading have varied	This clinical study was based on the measurement done on the OPG. There are inherent errors associated with the OPG in measuring linear displacements in horizontal direction. However, this was

							displacements where as on the delayed loading the displacement is tandem. The amount of extraction space closure is rapid for delayed loading as compared to the immediate loading in the first three months of retraction after loading. Further studies are warranted to directly asses the reciprocal effects of the different types of loading by utilizing modern state of art such as Cone Beam Computer Tomography (CBCT) imaging techniques.	overcome by calibration of magnification factor directly on the models of the same patients on whom OPG was taken. The angulation of implant insertion was not precisely mentioned. This may also contribute to the anchorage loss. The exact tissue reaction at the peri-mini-implant contact surface could not be determined by the present study.
**Bone density effects on the success rate of orthodontic microimplants evaluated with cone-beam computed tomography**	American Journal of Orthodontics and Dentofacial Orthopedics	Lee MY [75]	2016	South Korea	To evaluate the effect of cortical, cancellous, and total bone densities on the success rate of orthodontic microimplants using CBCT images.	Descriptive study	The success rates of OMIs increased as cancellous and total bone densities increased. Cortical bone density did not have a significant effect on the success of OMIs, but cancellous and total bone densities were significantly related to their success.	N/A
**Are assessments of damping capacity and placement torque useful in estimating root proximity of orthodontic anchor screws?**	American Journal of Orthodontics and Dentofacial Orthopedics	Motoyoshi M [76]	2016	Japan	To verify the detectability of root proximity of the screws by placement torque and damping capacity. For this purpose, we investigated the relationship among placement torque, damping capacity, and screw-root proximity.	Descriptive study	1. Root contact was found in 18% of self-drilling orthodontic anchor screws in the maxilla, and the success rate of the screws was about 96%. The placement torque was about 8 N/cm, and the average PTV was about 4.<br>2. The placement torque of screws with root contact was greater than that of screws with no root contact<br>3. The PTV of screws with root contact was significantly greater than that of screws with no root contact, and it was suggested that damping capacity is related to root contact.	N/A
**Comparison of the treatment effects of different rapid maxillary expansion devices on the maxilla and the mandible. Part 1: Evaluation of dentoalveolar changes**	American Journal of Orthodontics and Dentofacial Orthopedics	Canan S [77]	2017	Turkey	To compare the dentoalveolar treatment effects of 3 rapid maxillary expansion (RME) appliances, supported by different tissues, on the maxilla and the mandible.	Descriptive study	All 3 expanders led to the expansion of maxillary dentoalveolar structures with mild relapse. However, the amount of expansion of the bone-borne expander on the right side was statistically lower. Spontaneous interdental expansion was observed in the mandibular dentitions in all groups.	N/A
**Comparative evaluation of anchorage reinforcement between orthodontic implant**	Medical Journal Armed Forces India	Chopra SS [78]	2017	India	To evaluate the efficacy of orthodontic implant (OI) as anchorage reinforcement method when compared with conventional intraoral	Clinical trial	Implants as anchorage for en masse retraction can be incorporated into orthodontic practices with complete success. The use of OIs for anchorage is a viable alternative to conventional molar anchorage.	N/A

**and conventional anchorage in orthodontic management of bimaxillary dentoalveolar protrusion**					methods for anchorage reinforcement.			
**Treatment outcomes of Class II malocclusion cases treated with miniscrew anchored Forsus Fatigue Resistant Device: A randomized controlled trial**	The Angle Orthodontist	Eissa O [79]	2017	Egypt	To evaluate the skeletal, dental, and soft tissue effects of the Forsus Fatigue Resistant Device (FRD) used with miniscrew anchorage and compare them with those of the conventional Forsus FRD.	Clinical trial	Class II correction was mainly dentoalveolar in both treatment groups. Use of miniscrews with Forsus did not enhance mandibular forward growth nor prevent labial tipping of the mandibular incisors.	The small sample size together with patient attrition could have affected the accuracy of the results. The treatment duration may be not enough for mandibular growth to take place and may be considered as a drawback of the FRD.
**Efficiency of piezosurgery technique in miniscrew supported enmasse retraction: a single-centre, randomized controlled trial**	European Journal of Orthodontics	Tunçer NI [80]	2017	Turkey	To investigate the effects of piezo-surgery on retraction rates and biological response, by means of receptor activator of nuclear factor κβ ligand (RANKL) amount and concentration, which provide information about the osteclastogenesis activity, and GCF volume in miniscrew supported en-masse retraction cases. In addition to investigating the effects of piezosurgery on dental, skeletal and soft tissue changes on lateral cephalograms, on canine and molar rotations, and intermolar and intercanine widths on dental casts, besides the miniscrew success rates.	Clinical trial	1. No evidence was found to support the claim that piezosurgery technique is an efficient way of accelerating en-masse retraction.<br>2. Piezosurgery can alter the tissue reaction but no significant difference was present between the groups.<br>3. Changes in the nature of incisor and molar movement, cephalometric and dental cast variables were similar in two groups.<br>4. Miniscrew supported en-masse retraction is a feasible way of controlling the overbite and preserving anchorage during retraction.<br>5. Molars can be distalized in case of anchorage need during miniscrew supported en-masse retraction.<br>6. 1.5–1.4 mm diameter and 7 mm long AbsoAnchor miniscrews can be successfully used for en-masse retraction with 250g of force per side.	N/A
**Insertion torque and Periotest values are important factors predicting outcome after orthodontic**	American Journal of Orthodontics and Dentofacial Orthopedics	Watanabe T [81]	2017	Japan	To evaluate the stability of miniscrews after placement to identify factors influencing outcome in orthodontic	Descriptive study	The cortical bone thickness and the screw insertion torque are most likely factors influencing the causes of dislodgement of orthodontic anchor screws. Since Periotest values at screw placement	N/A

**miniscrew placement**					treatment using miniscrews.		were significantly lower in the success group than in the failure group, insertion torque and Periotest values may serve as important indexes in predicting the prognosis of screw placement when CBCT results are not available.	
**Assessment of immediate loading with mini-implant anchorage in critical anchorage cases between stainless steel versus titanium miniscrew implants: A controlled clinical trial**	Biomedical & Pharmacology Journal	Ashith MV [82]	2018	India	To compare the success rate between the mini-screw implant systems of two different materials viz. Titanium and Stainless steel under immediate loading for various applications in orthodontic treatment	Clinical trial	The result of this study showed that the success rate of titanium implants was more than stainless steel implants. Some of the probable reasons for failure of stainless steel implants might be peri-implantitis and less biocompatibility when compared to titanium implants. From the present study, it can be concluded that titanium implants can be successfully used as a temporary anchorage device.	N/A
**A cost-effectiveness analysis of anchorage reinforcement with miniscrews and molar blocks in adolescents: A randomized controlled trial**	European Journal of Orthodontics	Ganzer N [83]	2018	Sweden	To answer the following question: Will the use of miniscrews as anchorage reinforcement in adolescents reduce treatment costs compared with molar blocks?	Clinical trial	Compared with molar blocks, miniscrews provide better anchorage reinforcement but at a higher price. In cases with moderate need for anchorage reinforcement, the use of anchorage reinforcement with miniscrews is not cost-effective. Consequently, our results show that miniscrews cannot be used to make a standard treatment more cost-effective. Instead, miniscrews can be recommended for treatments where anchorage loss cannot be accepted.	One limitation of this trial is that both groups followed the same study protocol with identical appointment intervals.
**Anchorage reinforcement with miniscrews and molar blocks in adolescents: A randomized controlled trial**	American Journal of Orthodontics and Dentofacial Orthopedics	Ganzer N [84]	2018	Sweden	To evaluate anchorage capacity in its three dimensions at different timepoints: during leveling and alignment anchorage loss with and without molar blocks were evaluated (T1-T2); when molar blocks and buccal miniscrews were used during space closure for en masse retraction (T2- T3).	Clinical trial	Miniscrews as direct anchorage provided increased anchorage capacity with no statistically significant mesial movement during space closure. Thus, miniscrews can be recommended for anchorage reinforcement. Treatment with miniscrews resulted in movement of the maxillary first molars, characterized by distal rotation and buccal crown torque.<br>Molar blocks did not increase the anchorage capacity and thus cannot be recommended as anchorage reinforcement. The molar blocks caused movement of the maxillary first molars, characterized by mesial rotation and mesial tipping.	Our sample included 65% girls and 35% boys. One could argue that an even sex distribution would have eliminated the risk of bias caused by sex differences.
**A CBCT evaluation of molar uprighting by conventional versus microimplant-**	Dental Press Journal of Orthodontics	Martires S [85]	2018	India	To compare the effects of the conventional uprighting spring and of the mini-implant assisted molar	Clinical trial	1. Significant amount of molar uprighting can be attained by both conventional helical uprighting spring (CA group) and mini-implant assisted molar uprighting (MIA group)	N/A

**assisted methods: An in-vivo study**					uprighting spring, using the 3D CBCT scans.		methods, and is not affected by the type of anchorage used. 2. Mini-implant assisted molar uprighting (MIA group) was more effective in preventing the buccal movement of anchorage teeth and changes in the buccolingual inclination of the second molar, when compared to the conventional helical uprighting spring (CA group). 3. Mini-implant assisted molar uprighting (MIA) was more effective in preventing extrusion of the second molar in the vertical plane as compared to the conventional helical uprighting spring (CA group). 4. Molar uprighting in the conventional anchorage group (CA) occurred primarily by distal crown tipping whereas, in the mini-implant anchorage group (MIA), it occurred primarily by mesial root movement.	
**Effects of low-intensity laser therapy on the stability of orthodontic mini-implants: A randomised controlled clinical trial**	Journal of Orthodontics	Abohabib AH [86]	2018	Egypt	To investigate the effect of low-intensity laser light stimulation in promoting mini-implant stability during canine retraction with fixed appliances.	Clinical trial	(i) The overall success rate of mini-implants placed in this study was 78%, with both groups having the same success rate; (ii) The use of a low-intensity laser had a significant effect on resonance frequency values from week 3 to 10, but this did not affect clinical failure of mini-implants; (iii) Although some differences were noted in resonance frequency values there was no evidence of a clinical benefit in terms of implant stability in using low-intensity laser light following mini-implant placement.	N/A
**Titanium alloy vs stainless steel miniscrews: An in vivo split-mouth study**	European Review for Medical and Pharmacological Sciences	Bollero P [87]	2018	Italy	To compare TiA and SS miniscrews by analyzing: 1) the insertion and removal torque; 2) the morphological, structural, and compositional alterations in used orthodontic miniscrews derived from Scanning Electron Microscopic (SEM).	Clinical trial	TiA and SS miniscrews showed similar removal torque values. SEM photomicrographs of TiA TADs demonstrated blood cells covering most of the surface. SEM photomicrographs of SS TADs showed the precipitation of an amorphous layer with low cellular component. No evidence of osteo-integration was detected.	N/A
**Influence of antibiotic prophylaxis on the stability of orthodontic**	American Journal of Orthodontics and Dentofacial Orthopedics	Łyczek J [88]	2018	Poland	To investigate whether administration of antibiotic prophylaxis before the microimplantation	Clinical trial	The results of this pilot trial do not support administration of a single dose antibiotic prophylaxis before orthodontic microimplant placement, since no positive influence on the	The major limitation of this pilot trial was the small sample size because of reasons discussed above:

**microimplants: A pilot randomized controlled trial**					procedure improves the stability of the microimplants, reduces the soft tissue inflammation rate, and alleviates the pain after microimplant insertion. On the other hand, to evaluate the intensity of the general immunologic response to the tissue trauma from microimplantation and, in particular, to the inflammation of the tissues surrounding the microimplant, we included measurements of systemic inflammatory biomarker levels.		analyzed variables was observed in our sample. However, because of the small sample size, the results should be interpreted as preliminary until validated by a definitive, most possibly, multicenter trial including mandibular locations of the microimplants. What is more, we concluded that PCT and CRP measurements do not provide valuable information about the condition of the tissues surrounding the microscrew and are not an efficient tool for screening microimplant-related inflammations in the maxilla. Eventually, in our opinion, a full course of antibiotic treatment with microimplant application should be avoided, because the improvement of microscrew survival does not balance the profound negative effects of antibiotic use: i.e., adverse reactions and antibiotic resistance.	reluctance of the eligible subjects to have quadruple blood testing and their refusal to participate.
**Success rates and factors associated with failure of temporary anchorage devices: A prospective clinical trial**	Journal of Investigative and Clinical Dentistry	Aly SA [89]	2018	Egypt	To investigate success rates and all associated factors affecting TAD failure in different biomechanical needs over time.	Clinical trial	In conclusion, TADs have a good success rate and are beneficial to be used in orthodontic treatment planning. Patient age is a significant factor that should be considered during planning for TADs as a part of orthodontic treatment. Good oral hygiene has been proven to be an important factor in the success of TADs. Finally, the immediate loading of TADs is a safe technique, with a greater success rate than delayed loading, and can withstand up to 250 g with a good success rate.	N/A
**Stability of secondarily inserted orthodontic miniscrews after failure of the primary insertion for maxillary anchorage: Maxillary buccal area vs midpalatal suture area**	American Journal of Orthodontics and Dentofacial Orthopedics	Uesugi S [90]	2018	Japan	To investigate the success rates of primary and secondary insertions of orthodontic miniscrews used for maxillary anchorage, compare the stability of miniscrews inserted into the MB and the MP, and consider the risk factors associated with their instability.	Descriptive study	Although the success rate of the secondary insertion was significantly lower than that of the primary insertion into the MB, miniscrews inserted into the MP were stable in both primary and secondary insertions. The screw length was significantly associated with the stability of miniscrews inserted into the MB.	Unfortunately, this was not a randomized controlled trial. The sizes of the miniscrews were chosen with consideration of the distance from dental roots and bone tissue structures based on 3-dimensional computed tomography to avoid root injury and to minimize damage to the surrounding tissues. Therefore, the relationships among the

								length, diameter, and stability of orthodontic screws are not clear.
**Bone-anchored maxillary protraction to correct a class III skeletal relationship: A multicenter retrospective analysis of 218 patients**	Journal of Cranio-Maxillo-Facial Surgery	Hevele JV [91]	2018	Belgium	To evaluate the impact of class III correction by elastic traction on four miniplates and the failure rate of bone-anchored miniplates in nonsyndromic patients.	Retrospective study	When postoperative antibiotics were used, and the neck of the bone anchor was placed in the attached gingiva, failure rates were less. Miniplates placed in the maxilla failed six times as often as mandibular miniplates, and self-drilling screws had significantly fewer failures than self-tapping screws for fixing the miniplate.	N/A
**Evaluation of the success and complication rates of self-drilling orthodontic mini-implants**	Nigerian Journal of Clinical Practice	Gurdán Z [92]	2018	Hungary	To calculate the success and complication rates of orthodontic mini-implants.	Retrospective study	Inflammatory complications frequently develop even with careful insertion as a result of the patient’s poor oral hygiene. Our study findings showed that application of mini-screws of 1.6 mm × 8 mm inserted in the buccal fold is often associated with loosening of the implant upon immediate load. In contrast, mini-screws of palatal localization usually provide excellent skeletal anchorage.	N/A
**Influence of orthodontic mini-implant penetration of the maxillary sinus in the infrazygomatic crest region**	American Journal of Orthodontics and Dentofacial Orthopedics	Jia X [93]	2018	China	To investigate the incidence of penetration of mini-implants into the sinus and the relationship between penetration depth and sinus tissue.	Retrospective study	The incidence of penetration of infrazygomatic crest miniimplants into the sinus may be high. Penetration through double cortical bone plates with limitation of the penetration depth within 1 mm is recommended for infrazygomatic crest mini-implant anchorage.	N/A
**Insertion torque values and success rates for paramedian insertion of orthodontic mini-implants**	Journal of Orofacial Orthopedics	Di Leonardo B [94]	2018	Italy	To analyze the insertion torque values for self-drilling OMIs in the paramedian region, (2) to reveal possible correlations between insertion torque and vertical skeletal morphology, and (3) to test the hypothesis that an insertion torque >10 Ncm will increase OMI failure.	Retrospective study	No significant differences were found between insertion torque values with respect to the right and left sides, Jarabak’s ratio, facial axis, and Frankfort to mandibular plane angle. There were no significant differences in the OMIs insertion torques with regard to the different appliances. No association was found between insertion torque and vertical skeletal morphology.	N/A
**Success rates of a skeletal anchorage system in orthodontics: A retrospective analysis**	The Angle Orthodontist	Lam R [95]	2018	Australia	To evaluate the premise that skeletal anchorage with SAS miniplates is highly successful and predictable for a range of complex orthodontic movements.	Cross-sectional study	40% of cases experienced mild complications. The most common complication was soft tissue inflammation, which was amenable to focused oral hygiene and antiseptic rinses. Infection occurred in approximately 15% of patients where	As a retrospective cross-sectional study, there were limitations in the data. The observation time and time required to achieve the desired tooth movements varied with each case. Because of

							there was a statistically significant correlation with poor oral hygiene.	the small number of failures, there was insufficient statistical power to determine if any factor was significantly associated with the six plates that failed.
**Failure rates of miniscrews inserted in the maxillary tuberosity**	Dental Press Journal of Orthodontics	Azeem M [96]	2019	Pakistan	To investigate the failure rates of miniscrews inserted in the MT region, and to evaluate the associated factors.	Retrospective study	A 26.3% failure rate of mini-implants inserted in the MT regio was observed. Mini-implants were more successful when inserted in the MT region by experienced operators.	N/A
**Comparing the clinical success rate of self-drilling and self-tapping mini-screws in the retraction of maxillary anterior teeth**	Pesquisa Brasileira em Odontopediatria e Clínica Integrada	Sabzijati M [97]	2019	Iran	To examine this issue given the controversial information available in these two methods, which could be judged clinically in each case. In this study, the success rate of the mini-screws used in self-tapping and self-drilling methods in class II patients was evaluated.	Clinical trial	There is no difference between the self-drilling and self-tapping methods in the maxilla and the selection of each method is up to the dentist. However, it is better to advise patients to fully observe their health to control surrounding soft tissues for the possible inflammation. In the case of inflammation, it is not necessary to bring the screws out in the absence of mobility. In addition, the pain caused by this process is low despite the numbness. According to various studies, it is recommended to spend 2 to 4 weeks between applying force and implantation.	N/A
**Maxillary protraction with rapid maxillary expansion and facemask versus skeletal anchorage with mini-implants in class III patients: a non-randomized clinical trial**	Progress in Orthodontics	de Souza RA [98]	2019	Brazil	To test whether conventional orthodontic mini-implants inserted in the maxilla and mandible, associated with intermaxillary elastics would serve as anchorage for protracting the maxilla in class III patients with deficiency of the middle third of the face, and compare the results with those of patients treated with rapid maxillary expansion and facemask.	Non-randomized clinical trial	Conventional orthodontic mini-implants associated with intermaxillary elastics may be a treatment option for class III patients with maxillary retrusion. Most of the mini-implants remained stable during treatment. The mini-implant protocol reduced the undesirable effects of the conventional technique, within a shorter treatment time.	N/A
**Does audiovisual information affect anxiety and perceived pain levels in miniscrew**	Progress in Orthodontics	Calik Koseler B [99]	2019	Turkey	To evaluate the effects of providing verbal or audiovisual information on patients' anxiety	Clinical trial	According to the results of this study, verbal and audiovisual information had similar effects on pain perception. However, the audiovisual method caused more anxiety. The reasons for	N/A

**application? A within-person randomized controlled trial**					levels before miniscrew application.		these results should be investigated to better determine how verbal and audiovisual information affect patients and how they should be presented in order to provide the most comfortable experience possible.	
**Effect of photobiomodulation on the stability and displacement of orthodontic mini-implants submitted to immediate and delayed loading: A clinical study**	Lasers in Medical Science	Marañón-Vásquez GA [100]	2019	Brazil	To evaluate the effect of PBM on these outcomes. The influence of the loading protocol, as modifying factor of the PBM effect, was also assessed.	Non-randomized clinical trial	Delayed loading potentiated the effect of photobiomodulation therapy. The mini-implants that received these two interventions together presented the lowest loss of stability. Neither photobiomodulation therapy nor the loading protocol influenced the displacement of devices.	N/A
**Evaluation of the miniplate-anchored Forsus Fatigue Resistant Device in skeletal Class II growing subjects: A randomized controlled trial**	The Angle Orthodontist	Elkordy SA [101]	2019	Egypt	To compare the skeletal and dental effects of FFRD alone or in conjunction with miniplates in the treatment of skeletal Class II malocclusion as compared with untreated Class II controls.	Clinical trial	The addition of miniplates to the FFRD (FMP group) enhanced the skeletal outcome of Class II malocclusion treatment in the short term. Miniplate-anchored FFRD (FMP) resulted in a significant lengthening of the mandible that was coupled with clockwise mandibular rotation, reducing the apparent sagittal correction. In contrast to the conventional FFRD, miniplate-anchored FFRD (FMP) showed retroclination of the mandibular incisors and no anchorage loss.	The additional cost is also an important disadvantage, rendering a cost-benefit analysis mandatory.
**A prospective, split-mouth, clinical study of orthodontic titanium miniscrews with machined and acid-etched surfaces**	The Angle Orthodontist	Park HJ [102]	2019	South Korea	To determine whether the success rate and primary stability of surface-treated miniscrews differed significantly from those of nontreated miniscrews. Specifically, this study investigated whether surface treatment of miniscrews may be one of the key factors that contribute to the success of miniscrews in clinical situations.	Clinical trial	The success rate of acid-etched surface miniscrews was 91.8% and that of machined surface miniscrews was 85.8% in the current study, but this difference was not statistically significant. There was no significant difference in primary stability according to surface treatment and jaw. Patients with an open bite or those who require distalization of the total dentition are predicted to have a high possibility of miniscrew failure.	N/A
**Mini-implant supported canine retraction with micro-osteoperforation: A split-mouth randomized clinical trial**	The Angle Orthodontist	Sivarajan S [103]	2019	Malaysia	To investigate MOP using mini-implant supported canine retraction with fixed appliances. This splitmouth randomized trial focused on canine retraction within the	Clinical trial	MOP was associated with statistically significantly increased overall canine retraction of 1.1 mm over a 16-week period of observation. There were only small differences in tooth movement when intervals of 4-, 8-, and 12-week MOP were used. Moderate pain was associated with MOP at 4-	Direct clinical measurement of canine retraction may have been less accurate than measurements from dental study casts or using three-dimensional superimposition.

					maxilla and mandible following the extraction of first premolar teeth, and the effects of multiple MOP carried out at specific time points during a 16-week period of observation. In addition, feedback was also collected from participants relating to their experience of MOP during treatment.		week intervals while only mild pain was perceived for intervals of 8 and 12 weeks. The increased canine retraction achieved using MOP over a 16-week period is unlikely to be clinically significant.	
**Distance to alveolar crestal bone: a critical factor in the success of orthodontic miniimplants**	Progress in Orthodontics	Haddad R [104]	2019	Lebanon	To evaluate the relationship between MI success rate and its proximity to AC, as well as the association between success and other factors including gender, age, jaw, side and site of placement, and MI type.	Retrospective study	The distance to alveolar crest was strongly associated with long-term stability. More apical placement of the MI from the crest would be compatible with a denser and thicker bucco-lingual/palatal bone level. Root proximity was not associated with the failure of MIs as suggested by previous studies.	The inability to control for various factors known to affect MI stability such as insertion torque, patient oral hygiene, local gingival inflammation, and smoking.
**Failure rates of mini-implants inserted in the retromolar area**	International Orthodontics	Azeem M [105]	2019	Pakistan	To evaluate the failure rates of mini-implants inserted in the RM area and to evaluate the factors affecting their stability.	Cohort study	The right side had significantly higher failure than the left side. Mini-implants with inflammation showed significantly more failure.	The main limitation of this study is its retrospective nature. limited sample, lack of blinding, and the use of variable implants/conditions throughout the cases.
**Sagittal skeletal correction using symphyseal miniplate anchorage systems**	Journal of Orofacial Orthopedics	Çubuk S [106]	2019	Turkey	To evaluate success rates and complications related to symphyseal miniplate anchorage systems used for treatment of Class 2 and Class 3 deformities.	Retrospective study	Symphyseal miniplates are successful anchorage units in both Class 2 and Class 3 patients. Infection, miniplate mobility, and mucosal hypertrophy may be associated with the orthodontic attachments used, orthodontic forces applied, or miniplate designs.	N/A
**Risk factors for failure of orthodontic mini-screws placed in the median palate**	Journal of Oral Science	Ichinohe M [107]	2019	Japan	To investigate the stability of mini-screws placed in the median palate.	Descriptive study	Mini-screws were stable when palatal cortical bone was thick, screw–suture distance was ≥1.5mm, and insertion depth was >4.5 mm (>50% of the total mini-screw length). These results indicate that sufficient CBT, deep insertion, and sufficient screw-suture distance are required for primary stability of mini-screws.	N/A
**Low-level laser therapy with a 635 nm diode laser affects orthodontic**	Journal of Clinical Medicine	Flieger R [108]	2020	Poland	To estimate clinically the influence of 635 nm diode laser on the stability of orthodontic	Clinical trial	Irradiation of peri-implant soft tissue using a 635-nm diode laser enhances secondary mini-implant stability after three days, one month, and two	N/A

**mini-implants stability: A randomized clinical split mouth trial**					mini-implants placed in a maxilla. Furthermore, mini-implants' failure rate (mini-implant loss) and a pain level after the treatment were evaluated.		months. The diode laser application has no significant effect on pain level after orthodontic appliance placement measured in the NRS-11.	
**Effects of micro-osteoperforations on intraoral miniscrew anchored maxillary molar distalization: A randomized clinical trial**	Journal of Orofacial Orthopedics	Gulduren K [109]	2020	Northern Cyprus	To evaluate the effect of micro-osteoperforations on the rate of maxillary molar distalization on randomly selected human subjects. The duration of the study was determined to be 12 weeks since it was thought that there would be sufficient tooth movement to determine the effects of MOPs during this period.	Clinical trial	The present analyses revealed a 1.17-fold increase in the rate of tooth movement in the MOP group compared with the contralateral side. However, no significant differences between the MOP and the independent control groups were revealed. MOPs showed an accelerating effect on the tooth movement but this effect seems to be lower than expected. The present analyses did not reveal any periodontal side effects, discomfort, eating difficulty, or speech problems caused by MOPs. Pain level was marginally increased by MOPs on the first day of application which was found to be statistically significant. No difference in pain scores was revealed at any other time points.	The observation period was rather short. Only a 3 month period of the overall orthodontic treatment was evaluated. The activity of inflammatory markers was not investigated. Although two miniscrews were coupled to prevent tipping, they may not be a perfect reference because they may be slightly displaced in bone due to the reciprocal forces (500g).
**RFA measurements of survival midpalatal orthodontic mini-implants in comparison to initial healing period**	Progress in Orthodontics	Nienkemper M [110]	2020	Germany	To clinically investigate the long-term stability of mini-implants inserted in the midsagittal suture of the anterior palate.	Descriptive study	The stability of midpalatal mini-implants does not change in the long term after the initial healing period. Also, 2 × 9 mm mini-implants seem appropriate for orthodontic anchorage as the stability of 11-mm implants was not higher. In the anterior palate, shorter implants (9mm vs 11 mm) with an equal diameter can be regarded as less invasive and therefore should be preferred over longer implants.	N/A
**Recognizing the peak bone mass (age 30) as a cutof point to achieve the success of orthodontic implants**	Odontology	Tseng YC [111]	2020	Taiwan	To investigate the success rate of orthodontic implants and their related factors including characteristics of patients (age≤30 years and age>30 years), locations of placement, dimensions of implants, modes of orthodontic force, etc.	Retrospective study	Orthodontic implant success rate showed no significant correlation with sex, malocclusion, facial pattern, implantation position, implant materials and sizes, loading time, or the methods of force application. Age 30 is a cutoff point to achieve the success of orthodontic implants. The success rates of older patients (age>30 years) were significantly lower than young patients (age≤30 years), especially in females.	N/A

Table S2. List of cited studies and their sample characteristics (n=103)

Title	Sample Origin	Age	Sex	Quantity	Intervention Site
**Patient's perception on mini-screws used for molar distalization**	Private practice	Mean age 30 years (range 21-39 years)	F: 19<br>M: 11	30 patients<br>30 MS	Maxilla
**Comparative study between conventional en-masse retraction (sliding mechanics) and en-masse retraction using orthodontic micro implant**	JSS Dental College and Hospital, Mysore, Karnataka, India	Mean age 16.00 ± 1.41 years (nonimplant group) and 17.35 ± 3.5 years (implant group)	F: 14	14 patients<br>28 MCI	Maxilla
**Effect of smoking on the failure rates of orthodontic miniscrews**	Private practice	Mean age 36.9 years (range 13–64.5 years)	F: 49<br>M: 39	88 patients<br>110 MS	Maxilla and mandible
**Cone-beam computed tomography evaluation of mini-implants after placement: Is root proximity a major risk factor for failure?**	Department of Orthodontics, Uijeongbu St Mary's Hospital, in Uijeongbu, Korea	Mean age 26 years	F:18<br>M: 7	25 patients<br>50 MI	Maxilla
**Midpalatal miniscrews for orthodontic anchorage: Factors affecting clinical success**	Department of Orthodontics, Samsung Medical Center, Seoul, Korea	Mean age 23.4 ± 8.0 years (range 8.1-56.2 years)	F: 101<br>M: 27	128 patients<br>210 MS	Maxilla
**Survival analysis of orthodontic mini-implants**	N/A	Mean age 27 years (range 12-51 years)	N/A	141 patients<br>260 MI	Maxilla
**Accurate pre-surgical determination for self-drilling miniscrew implant placement using surgical guides and cone-beam computed tomography**	Aichi-Gakuin University, Aichi, Nisshin, Japan	Mean age 23.8 years (range 10.7-45.5 years)	F: 13<br>M: 5	18 patients<br>44 MS	Maxilla and mandible
**Relationship between vertical skeletal pattern and success rate of orthodontic mini-implants**	Department of Orthodontics at Gachon Dental Hospital, Inchon, Korea.	Range 10-55 years	F: 196<br>M: 110	306 patients<br>778 MI	Maxilla and mandible
**Factors affecting the long-term stability of orthodontic mini-implants**	Nihon University School of Dentistry, Tokyo, Japan	Mean age 26.1 ± 8.4 years (range 13.9-63.5 years)	F: 42<br>M: 10	52 patients<br>134 MI	Maxilla and mandible
**Noncompliance screw supported maxillary molar distalization in a parallel manner**	Cumhuriyet University, Turkey.	Mean age 14.9 years (range 13-19 years)	F: 11<br>M: 10	21 patients<br>42 MS	Maxilla
**A clinical evaluation of orthodontic mini-implants as intraoral anchorage for the intrusion of maxillary anterior teeth**	Department of Orthodontics, KLE University, Belgaum, India.	Range 14-24 years	N/A	10 patients<br>20 MI	Maxilla
**Clinical study of temporary anchorage devices for orthodontic treatment — Stability of Micro/Mini-screws and Mini-plates: Experience with 455 Cases—**	Tokyo Dental College Chiba Hospital	Mean age 25.7 ± 9.8 years (range 8-68 years)	F: 358<br>M: 97	455 patients<br>904 TADs	Maxilla and mandible
**Miniscrew implant-supported maxillary canine retraction with**	Dental School at Cairo University, Cairo, Egypt.	Mean age 19 years	F: 8<br>M: 5	13 patients<br>26 MS	Maxilla

**and without corticotomy-facilitated orthodontics**					
**Assessment of mini-implant displacement using cone beam computed tomography**	Department of Orthodontics of Rio de Janeiro Federal University, Brazil.	F: Mean age 29 years 7 months<br>M: Mean age 31 years 4 months	F: 10<br>M: 5	15 patients<br>41 MI	Maxilla
**Effects of mandibular incisor intrusion obtained using a conventional utility arch vs bone anchorage**	Department of Orthodontics, Faculty of Dentistry, Başkent University, Ankara, Turkey	N/A	N/A	13 patients<br>26 MI	Mandible
**Orthopedic correction of growing hyperdivergent, retrognathic patients with miniscrew implants**	Orthodontic Clinic of Baylor College of Dentistry, Dallas, Texas	Mean age 13.2 ± 1.1 years	F: 8<br>M: 1	9 patients<br>18 MS	Maxilla and mandible
**Comparison of movement of the upper dentition according to anchorage method: Orthodontic mini-implant versus conventional anchorage reinforcement in Class I malocclusion**	The Institute of Oral Health Science, Samsung Medical Center, Sungkyunkwan University, School of Medicine, Seoul, South Korea	Mean age 23.32 years (range 18-35 years)	F: 40	20 patients<br>40 MI	Maxilla
**Displacement pattern of the maxillary arch depending on miniscrew position in sliding mechanics**	Yonsei University Dental Hospital or Samsung Medical Center, Seoul, South Korea	Mean age 22.17 ± 5.54 years	F: 36	36 patients<br>72 MS	Maxilla
**Expectations, acceptance and preferences of patients in treatment with orthodontic mini-implants. Part II: Implant removal**	N/A	Mean age 15 years	F: 14<br>M: 11	25 patients	Maxilla
**Predictors of initial stability of orthodontic miniscrew implants**	Graduate Orthodontic Clinic of Chonnam National University Hospital, Gwangju, South Korea	Mean age 23.0 ± 8.7 years	F: 117<br>M: 51	168 patients<br>407 MS	Maxilla and mandible
**Factors influencing the stability of miniscrews. A retrospective study on 300 miniscrews**	Private practice	Mean age 23.2 years (F: Mean age 25.9 ± 11.6 years)<br>(M: Mean age 19.6 ± 10.1)	F: 80<br>M: 52	132 patients<br>300 MS	Maxilla and mandible
**Treatment effects of microimplant-aided sliding mechanics on distal retraction of posterior teeth**	Department of Kyungpook National University Hospital, Daegu, South Korea	Mean age 22.16 ± 5.17 years	F: 14<br>M: 9	23 patients<br>70 MCI and 12 MCS	Maxilla and mandible
**Success rate of microimplants in a University orthodontic clinic**	Department of Orthodontics and Dentofacial Orthopaedics, Manipal College of Dental Sciences, Manipal University, Manipal, India	Mean age 22.45 years (range 13-50 years)	F: 48<br>M: 25	73 patients<br>139 MCI	Maxilla and mandible
**Placement and removal torque values of orthodontic miniscrew implants**	Department of Orthodontics, Faculty of Dentistry, Chiang Mai University, Chiang Mai, Thailand.	Mean age 25.6 ± 6.7 years	F: 55<br>M: 40	95 patients<br>280 MS	Maxilla and mandible
**The effect of drill-free and drilling methods on the stability of mini-implants under early orthodontic loading in adolescent patients**	Institute of Health Sciences, Gazi University, Ankara, Turkey.	Mean age 15.7 ± 4.2 years	F: 38<br>M: 24	62 patients<br>112 MI	Maxilla

**Alveolar bone density change around miniscrews: A prospective clinical study**	Dental Teaching Clinics, Jordan University of Science and Technology, Irbid, Jordan.	Mean age 17 years 8 months (range 14-24 years).	F: 15<br>M: 7	22 patients<br>44 MS	Maxilla
**Dentofacial effects of two facemask therapies for maxillary protraction - Miniscrew implants versus rapid maxillary expanders**	ShenZhen Children's Hospital, Shenzhen, Guangdong Province, China.	Mean age 10 years 5 months	F: 14<br>M: 11	25 patients<br>50 MS	Maxilla
**A comparitive clinical study between self tapping and drill free screws as a source of rigid orthodontic anchorage**	Department of Orthodontics and Dentofacial Orthopedics, KLE Vishwanath Katti Institute of Dental Sciences, Belgaum, Karnataka, India.	Range 13–21 years	F: 15<br>M: 5	20 patients<br>40 MS	Maxilla
**Prognostic parameters contributing to palatal implant failures: A long-term survival analysis of 239 patients**	Private practice	Mean age 20.6 years (range 10-65 years)	F: 158<br>M: 81	239 patients<br>239 MS	Maxilla
**Comparison between Herbst appliances with or without miniscrew anchorage**	Private practice	Mean age 11.8 ± 1.7 years	F: 23<br>M: 27	50 patients<br>100 MS	Mandible
**Root proximity and cortical bone thickness effects on the success rate of orthodontic micro-implants using cone beam computed tomography**	Department of Orthodontics, Wonkwang University Daejeon Hospital, Sin-dong, Iksan, South Korea	Mean age 19.36 ± 5.66 years	F: 61<br>M: 33	94 patients<br>172 MCI	Maxilla
**Bone density and miniscrew stability in orthodontic patients**	N/A	Mean age 18.9 ± 4.12 years	F: 8<br>M: 2	10 patients<br>38 MS	Maxilla and mandible
**Treatment effects of intrusion arches and mini-implant systems in deepbite patients**	Department of Orthodontics, School of Dentistry, Suleyman Demirel University, Isparta, Turkey	N/A	F: 9<br>M: 6	15 patients<br>30 MI	Maxilla
**Clinical factors correlated with the success rate of miniscrews in orthodontic treatment**	Private practice	Mean age 27.2 ± 7.3 years	F: 21<br>M: 13	34 patients<br>82 MS	Maxilla and mandible
**Mini-implants vs fixed functional appliances for treatment of young adult Class II female patients A prospective clinical trial**	University's Institute of Dental Sciences, Belgaum, India, and University of Connecticut Health Center.	Mean age 17.38 ± 2.85 years	F: 14	14 patients<br>28 MI	Maxilla
**Mini-implants in the palatal slope. A retrospective analysis of implant survival and tissue reaction**	Department of Orthodontics, University Hospital Münster, Germany.	Mean age 15.1 years	F: 22<br>M: 19	41 patients<br>66 MI	Maxilla
**A prospective comparative study between differential moments and miniscrews in anchorage control**	Orthodontic Clinic, University of Connecticut, United States.	Mean age 17.4 ± 8.85 years	F: 6<br>M: 7	13 patients<br>13 MS	Maxilla
**Influence of miniscrew dental root proximity on its degree of late stability**	Department of Orthodontics, Bauru Dental School, University of São Paulo, Brazil.	Mean age 16.99 ± 5.08 years	F: 12<br>M: 9	21 patients<br>40 MS	Maxilla
**Distalization pattern of the maxillary arch depending on the**	Orthodontic Department at Yonsei University Dental Hospital, Seoul, South Korea.	Mean age 23.5 ± 6.92 years	F: 22<br>M: 3	25 patients<br>76 MS	Maxilla

**number of orthodontic miniscrews**					
**Placement angle effects on the success rate of orthodontic microimplants and other factors with cone-beam computed tomography**	Department of Orthodontics, Wonkwang University Daejeon Hospital, Sin-dong, Iksan, South Korea	Mean age 19.24 years ± 6.6 years	F: 97<br>M: 33	130 patients<br>228 MCI	Maxilla
**Maxillary protraction using a hybrid hyrax-facemask combination**	University of Düsseldorf, Germany	Mean age 9.5 ± 1.3 years	F: 6<br>M: 10	16 patients<br>32 MS	Maxilla
**Root proximity and inclination of orthodontic mini-implants after placement: Cone-beam computed tomography evaluation**	Nihon University School of Dentistry, Tokyo, Japan	Mean age 21.8 ± 5.7 years (range 13-34 years)	F: 35<br>M: 15	50 patients<br>147 MI	Maxilla and mandible
**Evaluation of optimal length and insertion torque for miniscrews**	Tohoku University Hospital, Sendai, Japan	Mean age 20.9 years (range 13.1-32.4 years)	F: 75<br>M: 30	105 patients<br>186 MS	Maxilla and mandible
**Orthodontic miniscrew failure rate and root proximity, insertion angle, bone contact length, and bone density**	Tohoku University Hospital, Sendai, Japan	Mean age 21 years (range 13.1–52.4 years)	F: 77<br>M: 30	107 patients<br>190 MS	Maxilla and mandible
**Zygomatic mini-implant for Class II correction in growing patients**	Cairo University, Egypt	Range 10–12 years	F: 10	10 patients<br>20 MI	Maxilla
**Factors affecting the clinical success of orthodontic anchorage: Experience with 266 temporary anchorage devices**	Department of Orthodontics, Taipei Mackay Memorial Hospital, Taiwan	Mean age 20.2 ± 9.4 years (range 12-52 years)	F: 75<br>M: 54	129 patients<br>266 TADs	Maxilla and mandible
**Assessment of damping capacity as an index of root proximity in self-drilling orthodontic mini-implants**	Nihon University School of Dentistry, Tokyo, Japan	Mean age 22.3 ± 7.9 years (self-drilling group)<br>Mean age 23.6 ± 8.1 years (self-tapping group)	F: 28 M: 13 (self-drilling group)<br>F: 27 M: 11 (self-tapping group)	41 patients, 70 MI (self-drilling group)<br>38 patients, 73 MI (self-tapping group)	Maxilla
**Effectiveness of 3 methods of anchorage reinforcement for maximum anchorage in adolescents: A 3-arm multicenter randomized clinical trial**	Chesterfield Royal Hospital, Chesterfield, UK<br>Royal Derby Hospital, Derby, UK	Mean age 14.15 ± 1.25 years	F: 16<br>M: 11	27 patients	Maxilla and mandible
**Root proximity and stability of orthodontic anchor screws**	Nihon University School of Dentistry, Tokyo, Japan	Mean age 24.4 ± 8.5 years	F: 43<br>M: 15	58 patients<br>165 MS	Maxilla and mandible
**Comparative study of the primary stability of self-drilling and self tapping orthodontic miniscrews**	Nihon University School of Dentistry, Tokyo, Japan	Mean age 23.2 ± 7.7 years (self-tapping group)<br>Mean age 22.3 ± 7.4 years (self-drilling group)	F: 25 M: 10 (self-tapping group)<br>F: 24 M: 11 (self-drilling group)	70 patients<br>140 MS	Maxilla
**A comparison of tapered and cylindrical miniscrew stability**	Orthodontic Clinic, University Dental Hospital, Seoul, South Korea	Mean age 25.3 ± 8 years	F: 89<br>M: 43	132 patients<br>105 tapered MS<br>122 cylindrical MS	Maxilla and mandible
**Effectiveness of mini implant in three-dimensional control**	Department of Orthodontics, Meenakshi Ammal Dental College	Range 14-25 years	N/A	10 patients	Maxilla

**during retraction - A clinical study**	and Hospital, Meenakshi University, Tamil Nadu, India				
**Anchorage loss due to Herbst mechanics—preventable through miniscrews?**	University of Homburg/Saar, Germany	Mean age 12 ± 1.6 years	F: 3<br>M: 9	12 patients<br>24 MI	Mandible
**A new method to evaluate the positional stability of a self-drilling miniscrew**	Department of Orthodontics, Peking University School and Hospital of Stomatology, Beijing, China	Mean age 24 years (range 21-41 years)	F: 14<br>M: 6	20 patients<br>120 MS	Maxilla
**Effects of tooth root contact on the stability of orthodontic anchor screws in the maxilla: Comparison between self-drilling and self-tapping methods**	Nihon University School of Dentistry, Tokyo, Japan	Mean age 23.2 years ± 8 years	F: 54<br>M: 26	80 patients<br>142 MS	Maxilla
**Analysis of time to failure of orthodontic mini-implants after insertion or loading**	Dental Clinic of Seoul National University Bundang Hospital, Seongnam, Korea	Mean age 20.08 ± 7.52 years	F and M (not specified quantity)	134 patients<br>331 MI	Maxilla and mandible
**Three-dimensional analysis of the distal movement of maxillary first molars in patients fitted with mini-implant-aided transpalatal arches**	Hamadan University of Medical Sciences, Hamadan, Iran	Mean age 19.8 ± 6.3 years (range 12-36 years)	F: 22<br>M: 4	26 patients<br>52 MI	Maxilla
**Maxillary sinus perforation by orthodontic anchor screws**	Nihon University School of Dentistry, Tokyo, Japan	Mean age 23.3 ± 8.9 years	F: 28<br>M: 17	45 patients<br>82 MS	Maxilla
**Effect of the length of orthodontic mini-screw implants on their long-term stability: A prospective study**	Wroclaw Medical University, Wrocław, Poland	Range 20–29 years	F: 27<br>M: 0	27 patients<br>54 MS	Mandible
**Failure rates of mini-implants placed in the infrazygomatic region**	Orthodontic Clinic, University of Connecticut, United States.	Mean age 22.2 ± 11 years	F: 42<br>M: 13	30 patients<br>55 MI	Maxilla
**A study of success rate of miniscrew implants as temporary anchorage devices in Singapore**	National Dental Centre of Singapore, Singapore	> 20 and <20 (not specified quantity)	F and M (not specified quantity)	136 patients<br>285 MS	Maxilla and mandible
**Comparison of short-term effects between face mask and skeletal anchorage therapy with intermaxillary elastics in patients with maxillary retrognathia**	N/A	Mean age 11.75 ± 1.23 years	F: 13<br>M: 12	25 patients<br>50 MP<br>50 MI	Maxilla and mandible
**Comparison of anterior and posterior mini-implant assisted maxillary incisor intrusion: Root resorption and treatment efficiency**	School of Medicine, Ege University, Bornova, Turkey	Mean age 19.31 years ± 3.84	F: 20<br>M: 12	32 patients<br>64 MI	Maxilla
**Three-dimensional analysis of tooth movements after palatal miniscrew-supported molar distalization**	Department of Orthodontics of Gülhane Military Medical Academy, Ankara, Turkey	Mean age 13.6 years (range 12.3-15.3 years)	F: 9<br>M: 12	21 patients<br>42 MS	Maxilla

**Three-dimensional effects of the mini-implant–anchored Forsus Fatigue Resistant Device: A randomized controlled trial**	Faculty of Dentistry, Cairo University, Egypt	Mean age 13.07 ± 1.41 years	F: 15<br>M: 0	15 patients<br>30 MI	Mandible
**Comparison of anchorage pattern under two types of orthodontic mini-implant loading during retraction in type a anchorage cases**	Narayana Dental College, Nellore, Andhra Pradesh, India	Range 18-25 years	F: 12<br>M: 9	21 patients<br>42 MS	Maxilla
**Bone density effects on the success rate of orthodontic microimplants evaluated with cone-beam computed tomography**	Department of Orthodontics of Wonkwang University Dental Hospital, Daejeon, Korea	Mean age 19.2 years	F: 53<br>M: 18	71 patients<br>127 MI	Maxilla
**Are assessments of damping capacity and placement torque useful in estimating root proximity of orthodontic anchor screws?**	Nihon University School of Dentistry, Tokyo, Japan	Mean age 21.3 ± 6.9 years	F: 79<br>M: 31	110 patients<br>202 MS	Maxilla
**Comparison of the treatment effects of different rapid maxillary expansion devices on the maxilla and the mandible. Part 1: Evaluation of dentoalveolar changes**	Department of Orthodontics, Faculty of Dentistry, of S€uleyman Demirel Universit, Isparta, Turkey	Bone-borne group mean age 12.92 ± 1.07<br>Hybrid-group mean age 13.41 ± 0.88	F: 17<br>M: 14	31 patients<br>94 MI	Maxilla
**Comparative evaluation of anchorage reinforcement between orthodontic implant and conventional anchorage in orthodontic management of bimaxillary dentoalveolar protrusion**	Army Dental Centre, New Delhi, India	Mean age 15.12 ± 1.42 years	F: 13<br>M: 12	25 patients<br>100 MS	Maxilla and mandible
**Treatment outcomes of Class II malocclusion cases treated with miniscrew anchored Forsus Fatigue Resistant Device: A randomized controlled trial**	Faculty of Dentistry, Tanta University, Egypt	Mean age 12.52 years ± 1.12 years	F: 10<br>M: 5	15 patients<br>30 MS	Mandible
**Efficiency of piezosurgery technique in miniscrew supported enmasse retraction: a single-centre, randomized controlled trial**	Department of Orthodontics, Faculty of Dentistry, Başkent University, Ankara, Turkey	Range 14.3 to 25.6 years	F: 26<br>M: 4	30 patients<br>60 MS	N/A
**Insertion torque and Periotest values are important factors predicting outcome after orthodontic miniscrew placement**	N/A	Mean age 25.4 ± 10.5 years	F: 60<br>M: 0	60 patients<br>120 MS	Maxilla
**Assessment of immediate loading with mini-implant anchorage in critical anchorage cases between stainless steel versus titanium miniscrew**	N/A	Range 15-25 years	N/A	10 patients<br>20 MI	Maxilla and mandible

**implants: A controlled clinical trial**					
**A cost-effectiveness analysis of anchorage reinforcement with miniscrews and molar blocks in adolescents: A randomized controlled trial**	N/A	Range 11–19 years	F: 24<br>M: 11	35 patients<br>72 MS	Maxilla
**Anchorage reinforcement with miniscrews and molar blocks in adolescents: A randomized controlled trial**	Public Dental Service Orthodontic Clinic in Gävle, Region Gävleborg, Sweden	Mean age 16.3 ± 1.7 years	F: 21<br>M: 12	33 patients<br>66 MS	Maxilla
**A CBCT evaluation of molar uprighting by conventional versus microimplant-assisted methods: An in-vivo study**	Department of Orthodontics and Dentofacial Orthopedics, Goa Dental College and Hospital, India	Mean age 25.1 years	F: 7<br>M: 3	10 patients<br>10 MI	Mandible
**Effects of low-intensity laser therapy on the stability of orthodontic mini-implants: A randomised controlled clinical trial**	Department of Orthodontics, Faculty of Dentistry, Cairo University, Egypt	Mean age 20.9 ±3.4 years	N/A	14 patients<br>28 MI	Maxilla
**Titanium alloy vs stainless steel miniscrews: An in vivo split-mouth study**	Department of Orthodontics at the University of Rome, "Tor Vergata", Rome, Italy	Mean age 16.2 ± 4.6 years	F: 9<br>M: 6	15 patients<br>30 MS	Maxilla
**Influence of antibiotic prophylaxis on the stability of orthodontic microimplants: A pilot randomized controlled trial**	Department of Dentofacial Orthopedics and Orthodontics, Wroclaw Medical University, Poland	F: Mean age 20.2 ± 5.6 years<br>M: Mean age 21.2 ± 6.8 years	F: 29<br>M: 9	38 patients<br>76 MCI	Maxilla
**Success rates and factors associated with failure of temporary anchorage devices: A prospective clinical trial**	Department of Orthodontics, Future University, Cairo, Egypt	Mean age 21.41 years	F: 58<br>M: 24	82 patients<br>180 MS	Maxilla and mandible
**Stability of secondarily inserted orthodontic miniscrews after failure of the primary insertion for maxillary anchorage: Maxillary buccal area vs midpalatal suture area**	Orthodontic Department of Tokyo Medical, Dental University, Tokyo, Japan	Mean age 27.9 ± 8.4 years	F: 176<br>M: 62	238 patients<br>471 MS	Maxilla
**Bone-anchored maxillary protraction to correct a class III skeletal relationship: A multicenter retrospective analysis of 218 patients**	Multiple centers	Mean age 11.4 years (range 9-14 years)	F: 106<br>M: 112	218 patients<br>872 MP	Maxilla and mandible
**Evaluation of the success and complication rates of self-drilling orthodontic mini-implants**	Department of Paediatric and Adolescent Dentistry and Department of Oral and Maxillofacial Surgery, University of Pécs, Pécs, Hungary	N/A	F: 44<br>M: 15	47 patients<br>59 MS	Maxilla and mandible
**Influence of orthodontic mini-implant penetration of the maxillary sinus in the infrazygomatic crest region**	Department of Orthodontics of Beijing Friendship Hospital, Beijing, China	Mean age 28 ± 6 years	F: 22<br>M: 10	32 patients<br>60 MI	Maxilla

**Insertion torque values and success rates for paramedian insertion of orthodontic mini-implants**	University of Trieste, Italy	F: Mean age 17.31 years<br>M: Mean age 15.65 years	F: 26<br>M: 14	40 patients<br>100 MI	Maxilla
**Success rates of a skeletal anchorage system in orthodontics: A retrospective analysis**	Private practice	Mean age 29.4 ± 12.02 years	F: 95<br>M: 68	163 patients<br>421 MP	Maxilla and mandible
**Failure rates of miniscrews inserted in the maxillary tuberosity**	Faisalabad Medical University, Faisalabad, Pakistan	Mean age 20.1 ± 8.9 years	F: 23<br>M: 17	40 patients<br>60 MS	Maxilla
**Comparing the clinical success rate of self-drilling and self-tapping mini-screws in the retraction of maxillary anterior teeth**	Department of Orthodontics, Dental School, Shahed University of Medical Sciences, Tehran, Iran	Mean age 25.7 ± 4.4 years (range 17-36 years)	F: 49<br>M: 8	57 patients<br>114 MS	Maxilla
**Maxillary protraction with rapid maxillary expansion and facemask versus skeletal anchorage with mini-implants in class III patients: a non-randomized clinical trial**	N/A	Mean age 10 ± 1.8 years	F: 8<br>M: 4	12 patients<br>48 MI	Maxilla and mandible
**Does audiovisual information affect anxiety and perceived pain levels in miniscrew application? A within-person randomized controlled trial**	Bezmialem University, Istanbul, Turkey	Mean age 18.18 ± 5.39 years	F: 58<br>M: 30	88 patients<br>88 MS	N/A
**Effect of photobiomodulation on the stability and displacement of orthodontic mini-implants submitted to immediate and delayed loading: A clinical study**	School of Dentistry of Ribeirão Preto, University of São Paulo, Brazil	N/A	N/A	48 MI	Maxilla and mandible
**Evaluation of the miniplate-anchored Forsus Fatigue Resistant Device in skeletal Class II growing subjects: A randomized controlled trial**	Orthodontic Clinic, Faculty of Dentistry, Cairo University, Egypt	Mean age 12.1 ± 0.9 years	N/A	16 patients<br>32 MP<br>96 MS	Mandible
**A prospective, split-mouth, clinical study of orthodontic titanium miniscrews with machined and acid-etched surfaces**	Department of Orthodontics, Yonsei University Dental Hospital or Samsung Medical Center, Seoul, South Korea	Mean age 22.16 ± 5.38 years	F: 27<br>M: 13	40 patients<br>98 MS	Maxilla and mandible
**Mini-implant supported canine retraction with micro-osteoperforation: A split-mouth randomized clinical trial**	Department of Orthodontics at the University of Malaya	Mean age 22.2 ± 3.72 years	F: 23<br>M: 7	30 patients	Maxilla and mandible
**Distance to alveolar crestal bone: a critical factor in the success of orthodontic miniimplants**	N/A	Mean age 23.45 years (range 13–51.4 years)	F: 129<br>M: 131	260 patients<br>293 MI	Maxilla and mandible
**Failure rates of mini-implants inserted in the retromolar area**	Department of Orthodontics, Dental Section, Punjab Medical College,	Mean age 18.6 ± 5.2 years	F: 52<br>M. 55	102 patients<br>110 MI	Mandible

	Faisalabad, Medical University, Faisalabad, Pakistan				
**Sagittal skeletal correction using symphyseal miniplate anchorage systems**	Department of Orthodontics, Faculty of Dentistry, Başkent University, Ankara, Turkey	Mean age<br>12.1 ± 1.3 years (Forsus Fatigue Resistant Devices group)<br>11.3 ± 1.5 years (Intermaxillary elastics group)	F: 4 - M: 8 (Forsus Fatigue Resistant Devices group)<br>F: 7 - M: 10 (Intermaxillary elastics group)	29 patients<br>58 MP<br>174 MS	Mandible
**Risk factors for failure of orthodontic mini-screws placed in the median palate**	Nihon University School of Dentistry, Tokyo, Japan	Mean age 23.4 ± 5.6 years (range 15-34.5 years)	F: 18<br>M: 7	25 patients<br>50 MS	Maxilla
**Low-level laser therapy with a 635 nm diode laser affects orthodontic mini-implants stability: A randomized clinical split mouth trial**	Wroclaw Medical University, Wrocław, Poland	Mean age 32.5 ± 6.1 years	F: 13<br>M: 7	20 patients<br>40 MI	Maxilla
**Effects of micro-osteoperforations on intraoral miniscrew anchored maxillary molar distalization: A randomized clinical trial**	Near East University, Faculty of Dentistry, Department of Orthodontics, Nicosia, Northern Cyprus	Range 16.5-23.8 years	F: 7<br>M: 11	18 patients<br>36 MS	Maxilla
**RFA measurements of survival midpalatal orthodontic mini-implants in comparison to initial healing period**	Department of Orthodontics, Heinrich-Heine-University of Düsseldorf, Düsseldorf, Germany	Mean ages:<br>15.61 ± 6.96 years initial group 11mm MI<br>16.77 ± 7.75 years long-term group 11mm MI<br>15.54 ± 7.31 years initial group 9mm MI<br>16.21 ± 3.89 years long-term group 9mm MI	F: 36<br>M: 42	78 patients<br>78 MI	Maxilla
**Recognizing the peak bone mass (age 30) as a cutof point to achieve the success of orthodontic implants**	Department of Orthodontics, Kaohsiung Medical University, Kaohsiung, Taiwan	Mean age 26.8 ± 7.93 years	F: 211<br>M: 59	270 patients<br>426 MI	Maxilla and mandible

Table S3. List of cited studies and TADs characteristics (n=103)

Title	Success Rate	Failure Rate	Brand	Diameter	Length	System Type	Surgery Technique
**Patient's perception on mini-screws used for molar distalization**	N/A	N/A	Sin Implant Systems, São Paulo, Brasil	1.2mm	10mm	Self-tapped MS	Closed
**Comparative study between conventional en-masse retraction (sliding mechanics) and en-masse retraction using orthodontic micro implant**	71.4%	N/A	SK Surgical, Pune, India	1.3mm	8mm	Self-drilled MI	Closed
**Effect of smoking on the failure rates of orthodontic miniscrews**	N/A	18.2%	LOMAS type (Mondeal, Tuttlingen, Germany)	2mm	7 mm (n = 29), 9 mm (n = 50), 11 mm (n = 31)	Pre-drilled MS	Closed
**Cone-beam computed tomography evaluation of mini-implants after placement: Is root proximity a major risk factor for failure?**	N/A	N/A	SLA mini-implants C-implant, Cimplant Company, Seoul, Korea	1.8mm	8.5mm	Self-drilled MI	Closed
**Midpalatal miniscrews for orthodontic anchorage: Factors affecting clinical success**	90.80%	N/A	KLS-Martin, Jacksonville, Fla y Orthoplant, Biomaterials Korea, Seoul, Korea	1.5mm and 2mm	8mm and 9mm	Self-drilled MS	Closed
**Survival analysis of orthodontic mini-implants**	>90%	<10%	C-implant, Dentium, Seoul, Korea	1.8mm	8.5mm	N/A	N/A
**Accurate pre-surgical determination for self-drilling miniscrew implant placement using surgical guides and cone-beam computed tomography**	90.9%	N/A	Jeil Medical Corporation, Seoul, Korea	1.6mm	8mm	Self-drilled MS	Closed
**Relationship between vertical skeletal pattern and success rate of orthodontic mini-implants**	79%	N/A	Dual-Top Anchor System, Jeil Medical, Seoul, Korea	1.6mm	8mm	Self-drilled MI	Closed Opened
**Factors affecting the long-term stability of orthodontic mini-implants**	N/A	<9.5%	ISA orthodontic implants, Biodent, Tokyo, Japan	1.6mm	8mm	Self-drilled MI	Closed
**Noncompliance screw supported maxillary molar distalization in a parallel manner**	100%	N/A	M-5146, 11, Medartis AG, Basel, Switzerland	2mm	11mm	Self-drilled MS	Closed
**A clinical evaluation of orthodontic mini-implants as intraoral anchorage for the intrusion of maxillary anterior teeth**	100%	N/A	SK Surgical, Pune, India	1.3mm	8mm	Self-drilled MI	Closed
**Clinical study of temporary anchorage devices for orthodontic treatment —Stability of Micro/Mini-screws and Mini-plates: Experience with 455 Cases—**	>90%	Microscrews 7% Miniscrews 6% Palatal implants 11% Mini-plates 6%	Titanium self-drilling mini-screws (Dual Top Autoscrew®, Jeil Medical Corp., Korea and OSAS®, DEWIMED CO. Ltd. Germany), pre-drilling	Pre-drilling micro-screws: 1.2mm Titanium alloy self-drilling mini screws: >1.4mm Pre-drilling palatal screws tapered: 1.9–2.2mm	N/A	Seld-drilled MS Pre-drilled MCI	Closed Opened

			micro-implan system (K1 system®, Dentsply-Sankin, Japan) and palatal screws (PIAS®, Tokyo Dental College, Japan).				
**Miniscrew implant-supported maxillary canine retraction with and without corticotomy-facilitated orthodontics**	93%	N/A	AbsoAnchor, Dentos, Daegu, Korea	1.3mm	8mm	Self-tapped MS	Closed
**Assessment of mini-implant displacement using cone beam computed tomography**	N/A	N/A	INP, São Paulo, Brazil	Buccal and palatal mini-implants 1.4mm<br>Midpalatal mini-implants 2mm	Buccal and palatal mini-implants 8mm<br>Midpalatal mini-implants 6mm	Pre-drilled and self-drilled MI	Closed
**Effects of mandibular incisor intrusion obtained using a conventional utility arch vs bone anchorage**	92.4%	N/A	AbsoAnchor, Dentos, Daegu, Korea	1.2mm	6mm	Self-drilled MI	Closed
**Orthopedic correction of growing hyperdivergent, retrognathic patients with miniscrew implants**	96.3%	N/A	IMTEC MSIs (IMTEC Corporation, Ardmore, OK)	1.8mm	8mm	Self-drilled MS	Closed
**Comparison of movement of the upper dentition according to anchorage method: Orthodontic mini-implant versus conventional anchorage reinforcement in Class I malocclusion**	N/A	N/A	Dual-Top Anchor System, Jeil Medical, Seoul, Korea	1.6mm	8mm	Self-drilled MI	Closed
**Displacement pattern of the maxillary arch depending on miniscrew position in sliding mechanics**	94.4%	N/A	Orlus, Ortholution Inc., Seoul, Korea<br>Orthoplant 2507T, BioMaterials Korea Inc., Seoul, Korea	1.8 mm - 2.5mm	7 mm	Self-tapped MS	Closed
**Expectations, acceptance and preferences of patients in treatment with orthodontic mini-implants. Part II: Implant removal**	N/A	16.7%	Tomas®-pins, Dentaurum, Ispringen, Germany	1.6mm	8mm	Self-drilled MI	Closed
**Predictors of initial stability of orthodontic miniscrew implants**	93.1%	N/A	Orlus, Ortholution Inc., Seoul, Korea	1.6 and 1.8mm	6,7,8 and 10mm	Self-drilled MS	Closed
**Factors influencing the stability of miniscrews. A retrospective study on 300 miniscrews**	81%	19%	MAS® system; Micerium, Avegno, Italy	1.3mm and 1.5mm	9mm and 11mm	Self-tapped MS	Closed
**Treatment effects of microimplant-aided sliding mechanics on distal retraction of posterior teeth**	89.7%	N/A	Absoanchor, Dentos, Daegu, Korea<br>Osteomed, Dallas, Texas	N/A	N/A	N/A	N/A
**Success rate of microimplants in a University orthodontic clinic**	87.8%	N/A	Indian Implants, S.K. Surgicals, Pune and Absoanchors, Dentos, Daugu, South Korea	1.3mm	8mm	N/A	Closed

**Placement and removal torque values of orthodontic miniscrew implants**	Pre-drilling 94.2% Self-drilling 92.5%	N/A	Sistema Nacional de Implantes, Sao Paulo, Brazil and ACR Mini-Implant, BioMaterials Korea, Guro-gu, Seoul, Korea.	1.5mm	6 and 8mm	Pre-drilled and self-drilled MS	Closed
**The effect of drill-free and drilling methods on the stability of mini-implants under early orthodontic loading in adolescent patients**	77.7%	N/A	AbsoAnchor, Dentos, Daegu, Korea	1.4mm	7mm	Pre-drilled and Self-drilled MI	Closed
**Alveolar bone density change around miniscrews: A prospective clinical study**	88.6%	11.4%	AbsoAnchor, Dentos, Daegu, Korea	1.3mm	8mm	Self-drilled MS	Closed
**Dentofacial effects of two facemask therapies for maxillary protraction - Miniscrew implants versus rapid maxillary expanders**	87.5%	N/A	ShenGang, ZhangHua, Taiwan	2mm	14mm	Self-drilled MS	Opened
**A comparitive clinical study between self tapping and drill free screws as a source of rigid orthodontic anchorage**	77.5%	N/A	Denticon, Mumbai.	1.4mm	8mm	Self-drilled and Pre-drilled MS	Closed
**Prognostic parameters contributing to palatal implant failures: A long-term survival analysis of 239 patients**	95.4%	N/A	Straumann, Basel, Switzerland	3.3, 4 and 4.1mm	4, 4.2 and 6mm	Self-drilled MS	Closed
**Comparison between Herbst appliances with or without miniscrew anchorage**	100%	N/A	MAS® system; Micerium, Avegno, Italy	1.5 or 1.3mm	11mm	N/A	Closed
**Root proximity and cortical bone thickness effects on the success rate of orthodontic micro-implants using cone beam computed tomography**	90.7%	N/A	AbsoAnchor, Dentos, Daegu, Korea	1.2–1.3mm	8mm	Self-drilled MCI	Closed
**Bone density and miniscrew stability in orthodontic patients**	Maxilla 100% Mandible 77.8%	N/A	AbsoAnchor, Dentos, Daegu, Korea	N/A	N/A	N/A	Closed
**Treatment effects of intrusion arches and mini-implant systems in deepbite patients**	90%	N/A	AbsoAnchor, Dentos, Daegu, Korea	1.3mm	5mm	Self-drilled MI	Closed
**Clinical factors correlated with the success rate of miniscrews in orthodontic treatment**	90.2%	N/A	Dual-Top Anchor System, Jeil Medical, Seoul, Korea	1.2 or 1.4mm	8 or 10mm	Self-drilled MS	Closed
**Mini-implants vs fixed functional appliances for treatment of young adult Class II female patients A prospective clinical trial**	N/A	N/A	N/A	1.3mm	8mm	N/A	Closed

**Mini-implants in the palatal slope. A retrospective analysis of implant survival and tissue reaction**	N/A	6%	Promedia Medizintechnik GmbH, Siegen, Germany	2mm	8mm	N/A	Closed
**A prospective comparative study between differential moments and miniscrews in anchorage control**	84%	N/A	N/A	1.8 or 2mm	8 or 9mm	N/A	N/A
**Influence of miniscrew dental root proximity on its degree of late stability**	90%	10%	AbsoAnchor, Dentos, Daegu, Korea	1.5mm	7mm	Self-drilled MS	Closed
**Distalization pattern of the maxillary arch depending on the number of orthodontic miniscrews**	86.6%	N/A	Orlus, Ortholution Inc., Seoul, Korea	1.8mm	7mm	N/A	Closed
**Placement angle effects on the success rate of orthodontic microimplants and other factors with cone-beam computed tomography**	87.7%	N/A	AbsoAnchor, Dentos, Daegu, Korea	1.2mm and 1.3mm	8mm	Self-drilled MCI	Closed
**Maxillary protraction using a hybrid hyrax-facemask combination**	100%	N/A	PSM Medical Solutions, Tuttlingen, Germany	2mm	9mm	Self-drilled MS	Closed
**Root proximity and inclination of orthodontic mini-implants after placement: Cone-beam computed tomography evaluation**	General 95.6% Maxilla 95.6% Mandible 93.7%	N/A	ISA orthodontic implants, Biodent, Tokyo, Japan	1.6mm	8mm	Self-drilled MI	Closed
**Evaluation of optimal length and insertion torque for miniscrews**	Maxilla 93.4% Mandible 70.3%	N/A	AbsoAnchor, Dentos, Daegu, Korea	1.3mm	5, 6 and 7mm	Self-tapped MS	Closed
**Orthodontic miniscrew failure rate and root proximity, insertion angle, bone contact length, and bone density**	N/A	N/A	AbsoAnchor, Dentos, Daegu, Korea	1.4 mm	5, 6 and 8mm	Pre-drilled MS	Closed
**Zygomatic mini-implant for Class II correction in growing patients**	95%	5%	OsteoCare™ Implant System, London, United Kingdom	1.8mm	9mm	Self-drilled MI	Opened
**Factors affecting the clinical success of orthodontic anchorage: Experience with 266 temporary anchorage devices**	97%	N/A	AbsoAnchor, Dentos, Daegu, Korea	1.6 and 2mm	8 and 10mm	Pre-drilled TADs	Closed
**Assessment of damping capacity as an index of root proximity in self-drilling orthodontic mini-implants**	Self-drilling 95.7% Self-tapping 94.5%	N/A	ISA orthodontic implants, Biodent, Tokyo, Japan	1.6mm	8mm	Self-drilled MI Self-tapped MI	Closed
**Effectiveness of 3 methods of anchorage reinforcement for maximum anchorage in**	N/A	N/A	American Orthodontics	1.6mm	8mm	Self-drilled TADs	Closed

**adolescents: A 3-arm multicenter randomized clinical trial**							
**Root proximity and stability of orthodontic anchor screws**	95%	N/A	ISA orthodontic implants, Biodent, Tokyo, Japan	1.6mm	8mm	Self-drilled MS	Closed
**Comparative study of the primary stability of self-drilling and self tapping orthodontic miniscrews**	96%	N/A	ISA orthodontic implants, Biodent, Tokyo, Japan	1.6mm	8mm	Self-drilled MS<br>Self-tapped MS	Closed
**A comparison of tapered and cylindrical miniscrew stability**	Tapered 82.9%<br>Cylindrical 80.3%	N/A	Biomaterials Korea, Seoul, Korea	1.5mm	7mm	Self-drilled MS	Closed
**Effectiveness of mini implant in three-dimensional control during retraction - A clinical study**	N/A	N/A	AbsoAnchor, Dentos, Daegu, Korea	1.3mm	8mm	N/A	N/A
**Anchorage loss due to Herbst mechanics—preventable through miniscrews?**	N/A	30%	Ortho Easy®, Forestadent, Germany	1.8mm	8mm	N/A	N/A
**A new method to evaluate the positional stability of a self-drilling miniscrew**	N/A	Loaded MS 5%<br>Unloaded MS 6.25%	Ci Bei Corporation, Zhejiang, China	1.6mm	11mm	Self-drilled MS	N/A
**Effects of tooth root contact on the stability of orthodontic anchor screws in the maxilla: Comparison between self-drilling and self-tapping methods**	Self-drilling 91.5%<br>Self-tapping 94.4%	N/A	ISA orthodontic implants, Biodent, Tokyo, Japan	1.6mm	8mm	Self-drilled MS<br>Self-tapped MS	Closed
**Analysis of time to failure of orthodontic mini-implants after insertion or loading**	N/A	17%	Miangan, Biomaterials Korea, Seoul, Korea	1.2mm	7mm	Self-drilled MI	Closed
**Three-dimensional analysis of the distal movement of maxillary first molars in patients fitted with mini-implant-aided transpalatal arches**	N/A	N/A	N/A	1.4, 1.6 or 2.0mm	8 to 10mm	Self-tapped MI	Closed
**Maxillary sinus perforation by orthodontic anchor screws**	N/A	6.1%	ISA orthodontic implants, Biodent, Tokyo, Japan	1.6mm	8mm	Self-drilled MS	Closed
**Effect of the length of orthodontic mini-screw implants on their long-term stability: A prospective study**	74%	N/A	Ortho Easy®, Forestadent, Germany	N/A	6 or 8mm	Pre-drilled MS	Closed
**Failure rates of mini-implants placed in the infrazygomatic region**	N/A	21.8%	Lomas (Mondeal, Tuttligen, Germany), Imtec (Unitek 3M, Monrovia, California), Aarhus (Medicon, Tuttligen, Germany),	1.5 or 1.8 mm<br>2 or 2.3mm	6 to 8mm or 9mm	Self-tapped MI	Closed

			Dual Top (RMO, Denver, Colorado)				
**A study of success rate of miniscrew implants as temporary anchorage devices in Singapore**	T1 94.7%<br>T2 83.3%	N/A	Vec-torTAS and AbsoAnchor	1.3, 1.4, 2mm	6, 7, 8, 10, 12mm	N/A	N/A
**Comparison of short-term effects between face mask and skeletal anchorage therapy with intermaxillary elastics in patients with maxillary retrognathia**	MP 100%<br>MI 88%	N/A	MP: Trimed®, Titanium Self Tapping Screw, Ankara, Turkey<br>MI: Absoanchor, Dentos Inc. Taegu City, Korea	MI: 1.6mm	MI: 10mm	Self-drilled MI	Opened
**Comparison of anterior and posterior mini-implant assisted maxillary incisor intrusion: Root resorption and treatment efficiency**	98.43%	N/A	Anchor Plus, Los Angeles, California, USA	1.4 and 1.6mm	6 and 7mm	Self-drilled MI	Closed
**Three-dimensional analysis of tooth movements after palatal miniscrew-supported molar distalization**	100%	N/A	Ortho Easy®, Forestadent, Germany	1.7mm	8mm	Self-drilled MS	Closed
**Three-dimensional effects of the mini-implant–anchored Forsus Fatigue Resistant Device: A randomized controlled trial**	N/A	N/A	3M Unitek	1.6mm	10mm	N/A	Closed
**Comparison of anchorage pattern under two types of orthodontic mini-implant loading during retraction in type a anchorage cases**	N/A	N/A	Tomas®-pins, Dentaurum, Ispringen, Germany	1.2mm	8mm	Self-drilled MS	N/A
**Bone density effects on the success rate of orthodontic microimplants evaluated with cone-beam computed tomography**	85%	N/A	AbsoAnchor, Dentos, Daegu, Korea	1.2-1.3mm	8mm	Self-drilled MI	Closed
**Are assessments of damping capacity and placement torque useful in estimating root proximity of orthodontic anchor screws?**	95.5%	N/A	Biodent, Tokyo, Japan	1.6mm	8mm	Self-drilled MS	N/A
**Comparison of the treatment effects of different rapid maxillary expansion devices on the maxilla and the mandible. Part 1: Evaluation of dentoalveolar changes**	97.87%	N/A	Yesanchor, Seoul, Korea	1.8mm	9mm	Pre-drilled MI	Closed
**Comparative evaluation of anchorage reinforcement between orthodontic implant and conventional anchorage in orthodontic management of bimaxillary dentoalveolar protrusion**	90.9%	N/A	N/A	N/A	N/A	Self-drilled MS	N/A

**Treatment outcomes of Class II malocclusion cases treated with miniscrew anchored Forsus Fatigue Resistant Device: A randomized controlled trial**	100%	N/A	MCT Tech, South Korea	1.6mm	10mm	N/A	Closed
**Efficiency of piezosurgery technique in miniscrew supported enmasse retraction: a single-centre, randomized controlled trial**	88.3%	N/A	AbsoAnchor, Dentos, Daegu, Korea	1.5–1.4mm	7mm	N/A	Closed
**Insertion torque and Periotest values are important factors predicting outcome after orthodontic miniscrew placement**	N/A	14.2%	Dual-Top Anchor System, Jeil Medical, Seoul, Korea	1.4mm	6mm	Self-drilled MS	Closed
**Assessment of immediate loading with mini-implant anchorage in critical anchorage cases between stainless steel versus titanium miniscrew implants: A controlled clinical trial**	90%	N/A	Stainless steel miniscrew implant (SK Surgical) and Titanium mini Screw implant (Dentos)	1.3mm	8mm	Self-drilled MI	Closed
**A cost-effectiveness analysis of anchorage reinforcement with miniscrews and molar blocks in adolescents: A randomized controlled trial**	96%	N/A	Health Development Company, Sarcedo, Italy	1.5mm	8-10mm	N/A	Closed
**Anchorage reinforcement with miniscrews and molar blocks in adolescents: A randomized controlled trial**	96%	N/A	Health Development Company, Sarcedo, Italy	1.5mm	8-10mm	Self-drilled MS	Closed
**A CBCT evaluation of molar uprighting by conventional versus microimplant-assisted methods: An in-vivo study**	N/A	N/A	S.K. Surgicals, Pune/India	1.5mm	8mm	Self-drilled MI	Closed
**Effects of low-intensity laser therapy on the stability of orthodontic mini-implants: A randomised controlled clinical trial**	78.5%	N/A	AbsoAnchor, Dentos, Daegu, Korea	1.5mm	8mm	Self-drilled MI	N/A
**Titanium alloy vs stainless steel miniscrews: An in vivo split-mouth study**	100%	N/A	TiA miniscrew (Spider Screw, Sarcedo, Vicenza, Italy) and a SS miniscrew (Leone, Florence, Italy)	1.5mm	8mm	Self-drilled MS<br>Self-tapped MS	Closed
**Influence of antibiotic prophylaxis on the stability of orthodontic microimplants: A pilot randomized controlled trial**	Intervention group 97.2%<br>Control group 95%	N/A	AbsoAnchor, Dentos, Daegu, Korea	1.2-1.3 mm	8 mm	Pre-drilled MCI	Closed
**Success rates and factors associated with failure of temporary anchorage devices: A prospective clinical trial**	82.2%	N/A	3M ESPE, Neuss, Germany<br>Bone screw, Jeil	1.5, 1.6, and 1.8mm	6, 8, and 10mm	Self-drilled TADs	Closed

			Medical, Seoul, Korea<br>Morelli, Sorocaba, Brazil				
**Stability of secondarily inserted orthodontic miniscrews after failure of the primary insertion for maxillary anchorage: Maxillary buccal area vs midpalatal suture area**	Buccal area 79.1%<br>Midpalatal suture 84.5%	Buccal area 20.9%<br>Midpalatal suture 15.5%	Dual-Top Anchor System, Jeil Medical, Seoul, Korea	1.4, 1.6 or 2mm	6 or 8mm	Self-drilled MS	Closed
**Bone-anchored maxillary protraction to correct a class III skeletal relationship: A multicenter retrospective analysis of 218 patients**	93.6%	N/A	Titalink, Belgium	2mm	5 or 7mm	Self-tapped or Self-drilled MS	Opened
**Evaluation of the success and complication rates of self-drilling orthodontic mini-implants**	89.8%	N/A	Jeil Medical Corporation, Seoul, Korea	1.6mm	8mm	Self-drilled MS	Closed
**Influence of orthodontic mini-implant penetration of the maxillary sinus in the infrazygomatic crest region**	96.7%	N/A	A1, Penghua, Taiwan	2mm	12 to 17mm	Self-drilled MS	Closed
**Insertion torque values and success rates for paramedian insertion of orthodontic mini-implants**	98.8%	N/A	Ortho Easy®, Forestadent, Germany	1.7mm	8mm	Self-drilled MI	Closed
**Success rates of a skeletal anchorage system in orthodontics: A retrospective analysis**	98.6%	N/A	Super Mini Anchor Platet (Dentsply Sankin, Tokyo, Japan).<br>I plate and Osteomed Screws (Osteomed, 3885 Arapaho Rd, Addison, TX, USA)	2mm	5mm	N/A	Opened
**Failure rates of miniscrews inserted in the maxillary tuberosity**	N/A	26.3%	N/A	1.3mm, 1.5mm	8mm, 10mm	Self-tapped MS	N/A
**Comparing the clinical success rate of self-drilling and self-tapping mini-screws in the retraction of maxillary anterior teeth**	93%	7%	Jeil Medical Corporation, Seoul, Korea	1.4mm	8mm	Self-drilled MS<br>Self-tapped MS	Closed
**Maxillary protraction with rapid maxillary expansion and facemask versus skeletal anchorage with mini-implants in class III patients: a non-randomized clinical trial**	N/A	16.7%	N/A	1.5mm	8 or 10mm	Self-drilled MI	Closed
**Does audiovisual information affect anxiety and perceived pain levels in miniscrew application? A within-person randomized controlled trial**	N/A	N/A	Aarhus System Miniscrews, American Orthodontics Washington, USA	1.5mm	8mm	Self-drilled MS	N/A
**Effect of photobiomodulation on the stability and displacement of orthodontic mini-implants**	N/A	N/A	Conexão, Arujá, SP, Brazil	1.5mm	8mm	Self-tapped MI	N/A

**submitted to immediate and delayed loading: A clinical study**							
**Evaluation of the miniplate-anchored Forsus Fatigue Resistant Device in skeletal Class II growing subjects: A randomized controlled trial**	N/A	13.3%	Stryker, Leibinger, GmbH & Co, Freiburg, Germany	2mm	8-10mm	N/A	Opened
**A prospective, split-mouth, clinical study of orthodontic titanium miniscrews with machined and acid-etched surfaces**	88.8%	N/A	Machined surface, OSSH1606; Osstem Implant, Busan, Korea. OSSH1606HE; Osstem Implant, Busan, Korea	1.6mm	6mm	Self-drilled MS	Closed
**Mini-implant supported canine retraction with micro-osteoperforation: A split-mouth randomized clinical trial**	N/A	N/A	Orlus, Ortholution Inc., Seoul, Korea	1.6mm	N/A	N/A	Closed
**Distance to alveolar crestal bone: a critical factor in the success of orthodontic miniimplants**	88.1%	11.9%	AbsoAnchor, Dentos, Daegu, Korea	1.4 or 1.8mm	8mm	Self-drilled MI	Closed
**Failure rates of mini-implants inserted in the retromolar area**	76.2%	23.2%	AbsoAnchor, Dentos, Daegu, Korea	1.3 or 2mm	8 or 10mm	Pre-drilled MI	Closed
**Sagittal skeletal correction using symphyseal miniplate anchorage systems**	87.9%	N/A	MPI-3000, Tasarım Medikal, Istanbul, Turkey. 55.MAN.003, Trimed, Ankara, Turkey	2mm	5 or 7mm	N/A	Opened
**Risk factors for failure of orthodontic mini-screws placed in the median palate**	75%	N/A	BIODENT, Tokyo, Japan	2mm	9mm	Pre-drilled MS	Closed
**Low-level laser therapy with a 635 nm diode laser affects orthodontic mini-implants stability: A randomized clinical split mouth trial**	100%	N/A	RMO, West Colfax Ave., Denver, CO, USA	1.4mm	10mm	Self-drilled MI	Closed
**Effects of micro-osteoperforations on intraoral miniscrew anchored maxillary molar distalization: A randomized clinical trial**	N/A	N/A	Benefit, PSM, Tuttlingen, Germany	2mm	9 or 11mm	N/A	Closed
**RFA measurements of survival midpalatal orthodontic mini-implants in comparison to initial healing period**	N/A	N/A	Benefit, PSM, Tuttlingen, Germany	2mm	9 or 11mm	Pre-drilled MI	Closed
**Recognizing the peak bone mass (age 30) as a cutof point to achieve the success of orthodontic implants**	89.2%	N/A	N/A	1.2, 1.5 or 2mm	8, 9, 10, 11 or 12mm	Pre-drilled and Self-drilled MI	Closed

Supplementary Table S4. List of cited studies and biomechanical characteristics of treatments (n=103)

Title	Placement Site	Loading Protocol	Force	Time of Treatment or Follow-up	Orthodontic Movements Type
**Patient's perception on mini-screws used for molar distalization**	Interradicular (between second premolar and first molar)	Delayed	300 g	6 to 9 months	Molar distalization
**Comparative study between conventional en-masse retraction (sliding mechanics) and en-masse retraction using orthodontic micro implant**	Interradicular (between second premolar and first molar)	Immediately	2 N	182 days	En-masse retraction
**Effect of smoking on the failure rates of orthodontic miniscrews**	Buccal aspect of the maxillary alveolar process and the buccal side of the mandible	Immediately	200 cN	The mean follow-up period was 9 months (range: 1–16 months)	Gap closure, intrusion, uprighting and distalization.
**Cone-beam computed tomography evaluation of mini-implants after placement: Is root proximity a major risk factor for failure?**	Interradicular (between second premolar and first molar)	N/A	N/A	N/A	En-masse retraction
**Midpalatal miniscrews for orthodontic anchorage: Factors affecting clinical success**	Midpalatal sutures in adult patients; parapalatal area in adolescents	Immediately	500 to 800 g	< 6 months to >18 months	Distalization, mesialization, intrusion, or retraction of anterior teeth, either singly or in combination.
**Survival analysis of orthodontic mini-implants**	Interradicular (between second premolar and first molar)	N/A	N/A	88 weeks average orthodontic treatment time.	En-masse retraction
**Accurate pre-surgical determination for self-drilling miniscrew implant placement using surgical guides and cone-beam computed tomography**	Interradicular	Delayed	150-250 g	20.4 months (minimum 7 months, maximum 45 months).	En-masse retraction
**Relationship between vertical skeletal pattern and success rate of orthodontic mini-implants**	Interradicular (between the maxillary first and second premolars; between the maxillary second premolar and first molar; between the maxillary first and second molars; between the mandibular first and second premolars; between the mandibular second premolar and first molar; between the mandibular first and second molars)	Delayed	150-200 g	Mean period of 12.21 ± 7.88 months	N/A
**Factors affecting the long-term stability of orthodontic mini-implants**	Interradicular	Immediately	2N	Less of 12 months	N/A
**Noncompliance screw supported maxillary molar distalization in a parallel manner**	Right and left of the incisive canal away from the midpalatal suture	Immediately	300 g	Average of 9.61 ± 2.1 months (ranging between 6 to 12 months)	Molar distalization
**A clinical evaluation of orthodontic mini-implants as intraoral anchorage for the**	Interradicular (between lateral incisors and canines)	Immediately	45 cN	4.0 ± 1.5 months	Incisor intrusion

**intrusion of maxillary anterior teeth**					
**Clinical study of temporary anchorage devices for orthodontic treatment —Stability of Micro/Mini-screws and Mini-plates: Experience with 455 Cases—**	Anterior and posterior alveolar region in maxilla and mandible; posterior alveolar palate region; median and paramedia suture; maxillary zygomatic buttres; nasomaxillary buttres; external oblique ridge; retro-molar region	Delayed	N/A	N/A	N/A
**Miniscrew implant-supported maxillary canine retraction with and without corticotomy-facilitated orthodontics**	Interradicular (between second premolar and first molar)	N/A	150 g	4 months	Canine retraction
**Assessment of mini-implant displacement using cone beam computed tomography**	Buccal and palatal interradicular areas	Delayed	200 g	5 months	Molar intrusion
**Effects of mandibular incisor intrusion obtained using a conventional utility arch vs bone anchorage**	Interradicular (between lateral incisors and canines)	Delayed	69-80 g	5 months	Incisor intrusion
**Orthopedic correction of growing hyperdivergent, retrognathic patients with miniscrew implants**	Maxilla: Parasagittal region of the palate mesial to the first molars Mandible: Interradicular between second premolar and first molar	Immediately	150 g	1.9 years	Molar intrusion
**Comparison of movement of the upper dentition according to anchorage method: Orthodontic mini-implant versus conventional anchorage reinforcement in Class I malocclusion**	Interradicular (between second premolar and first molar)	Delayed	N/A	25 months	En-masse retraction
**Displacement pattern of the maxillary arch depending on miniscrew position in sliding mechanics**	Interradicular (between second premolar and first molar, or between the first and second premolars)	Immediately	150 g	N/A	En-masse retraction
**Expectations, acceptance and preferences of patients in treatment with orthodontic mini-implants. Part II: Implant removal**	Interradicular (between second premolar and first molar)	N/A	N/A	N/A	En-masse retraction
**Predictors of initial stability of orthodontic miniscrew implants**	Maxillary buccal (mostly between the second premolar and first molar), palatal slope (between the maxillary second premolar and first molar or between the first and second molar), midpalatal (the midline of the palate corresponding to the area between the maxillary second premolar and first molar), mandibular buccal molar (between the second premolar and first molar or between the first and second molar), mandibular buccal canine (distal to canine), or other	Delayed	N/A	N/A	N/A

	(between the mandibular incisors or mandibular retromolar area)				
**Factors influencing the stability of miniscrews. A retrospective study on 300 miniscrews**	Buccal side and only a few on the palatal side, maxillary and mandibular arch mesial to the second premolars and distal to second premolars	Immediately	150 g or 250 g	N/A	N/A
**Treatment effects of microimplant-aided sliding mechanics on distal retraction of posterior teeth**	Maxillary buccal alveolar bone between the second premolars and the first molars; in the palatal slope between the first and second molars. In the mandible, distobuccally to the second molars; between first and second molars; between second premolar and the first molar	N/A	200 g	Mean treatment time was 20 ± 4.9 months (range, 13-30 months)	Distalization of the maxillary and mandibular dental arch
**Success rate of microimplants in a University orthodontic clinic**	Multiple sites in maxilla and mandible	Immediately and delayed	N/A	N/A	En-masse retraction of the 6 anterior teeth in arches, distalization of the molar teeth, protraction of the molar teeth, intrusion of the maxillary and mandibular incisors and molars, and en-masse distalization of the dental arches as per the requirement of the case.
**Placement and removal torque values of orthodontic miniscrew implants**	Dentoalveolar bone of the maxilla and the mandible, and midpalatal suture area	N/A	50 g	44 ± 11 weeks	En-masse retraction
**The effect of drill-free and drilling methods on the stability of mini-implants under early orthodontic loading in adolescent patients**	Interradicular (between second premolar and first molar)	Delayed	200 g	N/A	Molar distalization
**Alveolar bone density change around miniscrews: A prospective clinical study**	Interradicular (between second premolar and first molar)	Delayed	200 g	N/A	Distalization of the maxillary first molars.
**Dentofacial effects of two facemask therapies for maxillary protraction - Miniscrew implants versus rapid maxillary expanders**	Infrazygomatic area	Delayed	200-250 g	11 months	Maxillary protraction
**A comparitive clinical study between self tapping and drill free screws as a source of rigid orthodontic anchorage**	Interradicular (between second premolar and first molar)	Immediately	150-200 g	N/A	En-masse retraction
**Prognostic parameters contributing to palatal implant failures: A long-term survival analysis of 239 patients**	Mid-sagittal plane of the median region of the anterior palate	Delayed	1-6 N	Mean follow-up 33 months	Anchorage purposes
**Comparison between Herbst appliances with or without miniscrew anchorage**	Mandibular bone at the level of marginal or attached gingiva or mucogingival junction, between the lower first molar and second premolar	Delayed	100 g	N/A	Class II correction with Herbst appliance

**Root proximity and cortical bone thickness effects on the success rate of orthodontic micro-implants using cone beam computed tomography**	Maxillary buccal alveolar bone	Immediately	50-200 g	1 year	N/A
**Bone density and miniscrew stability in orthodontic patients**	Interradicular (between second premolar and first molar)	Delayed	2 N	N/A	En-masse retraction
**Treatment effects of intrusion arches and mini-implant systems in deepbite patients**	Interradicular (between lateral incisors and canines)	Immediately	90 g (minimum,35 g; maximum, 50 g)	7 months	Incisor intrusion
**Clinical factors correlated with the success rate of miniscrews in orthodontic treatment**	Interradicular at the buccal side of maxilla and mandible, palatal, retromolar area	Delayed	0.98–1.96 N	14 months	Intrusion, protraction, uprighting, retraction.
**Mini-implants vs fixed functional appliances for treatment of young adult Class II female patients A prospective clinical trial**	Interradicular	Immediately	150 g	N/A	En-masse retraction
**Mini-implants in the palatal slope. A retrospective analysis of implant survival and tissue reaction**	Palatal slope	N/A	N/A	N/A	Mesialization, indirect anchorage in extraction cases, vertical and transversal movements
**A prospective comparative study between differential moments and miniscrews in anchorage control**	Interradicular (between second premolar and first molar)	N/A	150 g	N/A	En-masse retraction
**Influence of miniscrew dental root proximity on its degree of late stability**	Interradicular (between second premolar and first molar)	Immediately	100-250 g	N/A	En-masse retraction
**Distalization pattern of the maxillary arch depending on the number of orthodontic miniscrews**	Group A: Between the maxillary second premolar and first molar. Group B: additional miniscrews were placed between the maxillary first and second premolars	N/A	Group A: 200 g. Group B: 400g	N/A	Distalization of the maxillary dental arch
**Placement angle effects on the success rate of orthodontic microimplants and other factors with cone-beam computed tomography**	Maxillary buccal alveolar bone	Immediately	50-200 g	1 year	En-masse retraction
**Maxillary protraction using a hybrid hyrax-facemask combination**	Palate	Delayed	N/A	5.8 ± 1.7 months	Maxillary protraction
**Root proximity and inclination of orthodontic mini-implants after placement: Cone-beam computed tomography evaluation**	Interradicular (between second premolar and first molar)	Immediately	2 N	N/A	N/A
**Evaluation of optimal length and insertion torque for miniscrews**	Interradicular (between second premolar and first molar)	Immediately	50-100 g	N/A	En-masse retraction
**Orthodontic miniscrew failure rate and root proximity, insertion angle, bone contact length, and bone density**	Interradicular (between second premolar and first molar)	Immediately and delayed	N/A	N/A	To correct crowing, protrusion or open-bite
**Zygomatic mini-implant for Class II correction in growing patients**	Infrazygomatic area	Delayed	300 g	6 months	Molar distalization

**Factors affecting the clinical success of orthodontic anchorage: Experience with 266 temporary anchorage devices**	N/A	Delayed	100-200 g	N/A	N/A
**Assessment of damping capacity as an index of root proximity in self-drilling orthodontic mini-implants**	Interradicular (between second premolar and first molar)	Immediately	2 N	N/A	Anchorage purposes
**Effectiveness of 3 methods of anchorage reinforcement for maximum anchorage in adolescents: A 3-arm multicenter randomized clinical trial**	Interradicular	N/A	90-100 g	Mean time 26.83 months (range 8.5-45.16 months)	En-masse retraction
**Root proximity and stability of orthodontic anchor screws**	Interradicular (between second premolar and first molar)	Immediately	2 N	Not specified	Anchorage purposes
**Comparative study of the primary stability of self-drilling and self tapping orthodontic miniscrews**	Interradicular (between second premolar and first molar)	Immediately	2 N	N/A	En-masse retraction
**A comparison of tapered and cylindrical miniscrew stability**	Multiple sites in maxilla and mandible	Immediately	200-250 g	15.3 months	N/A
**Effectiveness of mini implant in three-dimensional control during retraction - A clinical study**	Maximum thickness of infrazygomatic crest, and between the roots of second premolar and first molar in the upper arch	Delayed	150 g	N/A	En-masse retraction
**Anchorage loss due to Herbst mechanics—preventable through miniscrews?**	Interradicular (between second premolar and first molar)	N/A	N/A	4.6 ± 0.4 months	To prevent proinclination of incisors
**A new method to evaluate the positional stability of a self-drilling miniscrew**	Interradicular (between second premolar and first molar) Additional unloaded MS were inserted between the lateral incisor and canine, and between the first and second molars	N/A	N/A	11.8 months (en-masse retraction)	En-masse retraction
**Effects of tooth root contact on the stability of orthodontic anchor screws in the maxilla: Comparison between self-drilling and self-tapping methods**	Interradicular (between second premolar and first molar)	N/A	N/A	N/A	En-masse retraction
**Analysis of time to failure of orthodontic mini-implants after insertion or loading**	Interradicular (between second premolar and first molar)	N/A	N/A	52 weeks	N/A
**Three-dimensional analysis of the distal movement of maxillary first molars in patients fitted with mini-implant-aided transpalatal arches**	Interradicular (between second premolar and first molar)	Immediately	150-200 g	6.8 ± 2.8 months (distalization)	Molar distalization
**Maxillary sinus perforation by orthodontic anchor screws**	Interradicular (between second premolar and first molar)	Immediately	2 N	N/A	En-masse retraction
**Effect of the length of orthodontic mini-screw implants on their long-term stability: A prospective study**	Interradicular (between second premolar and first molar)	Delayed	100-150 g	N/A	En-masse retraction
**Failure rates of mini-implants placed in the infrazygomatic region**	Infrazygomatic area	N/A	≥ 150 g	13.67 ± 6.79 months	Incisor retraction, distalization, and intrusion

**A study of success rate of miniscrew implants as temporary anchorage devices in Singapore**	Anterior region, posterior region, retromolar, palate	Delayed	N/A	N/A	N/A
**Comparison of short-term effects between face mask and skeletal anchorage therapy with intermaxillary elastics in patients with maxillary retrognathia**	MP between mandibular lateral incisors and canines; MI between maxillary second premolars and first molars	Immediately	75 g<br>200 g after 3 weeks	Mean time 0.76 ± 0.09 in SA group	Maxillary protraction
**Comparison of anterior and posterior mini-implant assisted maxillary incisor intrusion: Root resorption and treatment efficiency**	Interradicular (between maxillary laterals and canines, and between the second premolars and first molars)	N/A	40 g	N/A	Incisor intrusion
**Three-dimensional analysis of tooth movements after palatal miniscrew-supported molar distalization**	Anterior palatal region	N/A	N/A	Mean time 5.30 ± 1.46 months (distalization)	Molar distalization
**Three-dimensional effects of the mini-implant–anchored Forsus Fatigue Resistant Device: A randomized controlled trial**	Interradicular (between mandibular canines and first premolars)	N/A	N/A	Mean time 5.34 ± 1.29 months (correction of class II)	Class II correction with Forsus appliance
**Comparison of anchorage pattern under two types of orthodontic mini-implant loading during retraction in type a anchorage cases**	Interradicular (between second premolar and first molar)	Immediately and delayed	150 g	2-3 months	En-masse retraction
**Bone density effects on the success rate of orthodontic microimplants evaluated with cone-beam computed tomography**	Interradicular (between second premolar and first molar or the first molar and second molar)	N/A	50-200 g	N/A	N/A
**Are assessments of damping capacity and placement torque useful in estimating root proximity of orthodontic anchor screws?**	Interradicular (between second premolar and first molar)	N/A	N/A	N/A	En-masse retraction
**Comparison of the treatment effects of different rapid maxillary expansion devices on the maxilla and the mandible. Part 1: Evaluation of dentoalveolar changes**	Palate	N/A	N/A	Bone-borne group mean time 6.64 ± 0.50 months<br>Hybrid-group mean time 6.80 ± 0.45 months	Maxillary expansion
**Comparative evaluation of anchorage reinforcement between orthodontic implant and conventional anchorage in orthodontic management of bimaxillary dentoalveolar protrusion**	Interradicular (between second premolar and first molar)	Delayed	N/A	21.16 ± 1.62 months	En-masse retraction
**Treatment outcomes of Class II malocclusion cases treated with miniscrew anchored Forsus Fatigue Resistant Device: A randomized controlled trial**	Interradicular (between mandibular canines and first premolars)	N/A	N/A	6.42 months	Class II correction with Forsus appliance

**Efficiency of piezosurgery technique in miniscrew supported enmasse retraction: a single-centre, randomized controlled trial**	Interradicular (between second premolar and first molar)	Immediately	250 g	9.33 ± 4.10 months	En-masse retraction
**Insertion torque and Periotest values are important factors predicting outcome after orthodontic miniscrew placement**	Interradicular, buccal and lingual (between second premolar and first molar)	Delayed	N/A	12 months	En-masse retraction
**Assessment of immediate loading with mini-implant anchorage in critical anchorage cases between stainless steel versus titanium miniscrew implants: A controlled clinical trial**	Interradicular (between second premolar and first molar)	Immediately	150 g	N/A	En-masse retraction
**A cost-effectiveness analysis of anchorage reinforcement with miniscrews and molar blocks in adolescents: A randomized controlled trial**	Interradicular (between second premolar and first molar)	Immediately	150 g	28.4 months treatment duration	En-masse retraction
**Anchorage reinforcement with miniscrews and molar blocks in adolescents: A randomized controlled trial**	Interradicular (between second premolar and first molar)	Immediately	150 g	N/A	En-masse retraction
**A CBCT evaluation of molar uprighting by conventional versus microimplant-assisted methods: An in-vivo study**	Interradicular (between first and second premolars)	Immediately	50 g	4 months follow-up	Molar uprighting
**Effects of low-intensity laser therapy on the stability of orthodontic mini-implants: A randomised controlled clinical trial**	Interradicular (between second premolar and first molar)	Immediately	150 g	10 weeks follow-up	Canine retraction
**Titanium alloy vs stainless steel miniscrews: An in vivo split-mouth study**	Interradicular (between second premolar and first molar)	Immediately	90-100 g	160.8 ± 23 days MS function	Canine retraction
**Influence of antibiotic prophylaxis on the stability of orthodontic microimplants: A pilot randomized controlled trial**	Interradicular (between second premolar and first molar)	Immediately	200 g	N/A	Distalization of the maxillary dental arch
**Success rates and factors associated with failure of temporary anchorage devices: A prospective clinical trial**	Multiple sites in maxilla and mandible	Immediately and delayed	50, 100, 150, 200, and 250 g	N/A	Anchorage purposes
**Stability of secondarily inserted orthodontic miniscrews after failure of the primary insertion for maxillary anchorage: Maxillary buccal area vs midpalatal suture area**	Molar buccal area and the midpalatal suture	N/A	N/A	N/A	Anchorage purposes
**Bone-anchored maxillary protraction to correct a class III skeletal relationship: A**	Zygomatic buttresses and between the second incisors and canines in the mandible	Delayed	100 g	Mean follow-up 19.6 ± 13.4 months	Class III correction

**multicenter retrospective analysis of 218 patients**					
**Evaluation of the success and complication rates of self-drilling orthodontic mini-implants**	Palate, buccal fold, ascending ramus	Immediately	150 g	Mean time 8.1 months (loading time)	Extrusion of impacted tooth, intrusion, distalization, uprighting of submerged tooth.
**Influence of orthodontic mini-implant penetration of the maxillary sinus in the infrazygomatic crest region**	Infrazygomatic area	Delayed	400-500 g	N/A	N/A
**Insertion torque values and success rates for paramedian insertion of orthodontic mini-implants**	Anterior palatal region	Delayed	2 or 2.35 N	N/A	Distalization, mesialization, rapid palatal expansion
**Success rates of a skeletal anchorage system in orthodontics: A retrospective analysis**	N/A	N/A	N/A	N/A	Intrusion, extrusion, distalization, mesialization.
**Failure rates of miniscrews inserted in the maxillary tuberosity**	Maxillar tuberosity	Immediately	100-150 g	N/A	Molar distalization
**Comparing the clinical success rate of self-drilling and self-tapping mini-screws in the retraction of maxillary anterior teeth**	Interradicular (between second premolar and first molar)	Delayed	N/A	9 weeks	En-masse retraction
**Maxillary protraction with rapid maxillary expansion and facemask versus skeletal anchorage with mini-implants in class III patients: a non-randomized clinical trial**	Interradicular (mesial to 16-26 and mesial to 33-43)	Immediately	100 g	N/A	Maxillary protraction
**Does audiovisual information affect anxiety and perceived pain levels in miniscrew application? A within-person randomized controlled trial**	Interradicular	N/A	N/A	N/A	N/A
**Effect of photobiomodulation on the stability and displacement of orthodontic mini-implants submitted to immediate and delayed loading: A clinical study**	Attached gingiva (not specified the site)	Immediately and delayed	N/A	3 months	N/A
**Evaluation of the miniplate-anchored Forsus Fatigue Resistant Device in skeletal Class II growing subjects: A randomized controlled trial**	Mandibular symphysis	Delayed	N/A	7.26 ± 1.74 months follow up period	Class II correction with Forsus appliance
**A prospective, split-mouth, clinical study of orthodontic titanium miniscrews with machined and acid-etched surfaces**	Interradicular	Delayed	100-200 g	N/A	En-masse retraction
**Mini-implant supported canine retraction with micro-osteoperforation: A split-mouth randomized clinical trial**	Interradicular (between second premolar and first molar)	N/A	N/A	N/A	En-masse retraction

**Distance to alveolar crestal bone: a critical factor in the success of orthodontic miniimplants**	Interradicular (between canines and first premolars, first and second premolars, second premolars and permanent first molars, first and second permanent molars)	Immediately and delayed	N/A	N/A	N/A
**Failure rates of mini-implants inserted in the retromolar area**	Retromolar area	Immediately	100-150 g	12.11 ± 7.77 months MI loading	Molar uprighting
**Sagittal skeletal correction using symphyseal miniplate anchorage systems**	Mandibular symphysis	Delayed	N/A	Mean time for MP 12.7 ± 1.7 months (Forsus Fatigue Resistant Devices group) 7.6 ± 2.3 months (Intermaxillary elastics group)	Class II and Class III correction
**Risk factors for failure of orthodontic mini-screws placed in the median palate**	Anterior palatal region	Immediately	2-4 N	N/A	N/A
**Low-level laser therapy with a 635 nm diode laser affects orthodontic mini-implants stability: A randomized clinical split mouth trial**	Interradicular (between second premolar and first molar)	N/A	N/A	2 months observation period	En-masse retraction
**Effects of micro-osteoperforations on intraoral miniscrew anchored maxillary molar distalization: A randomized clinical trial**	Anterior palatal region	Delayed	N/A	12 weeks	Molar distalization
**RFA measurements of survival midpalatal orthodontic mini-implants in comparison to initial healing period**	Anterior palatal region	N/A	N/A	Mean time treatment 3.17 ± 0.96 years 11mm MI group Mean time treatment 2.84 ± 1.25 years 9mm MI group	Sagittal molar movement
**Recognizing the peak bone mass (age 30) as a cutof point to achieve the success of orthodontic implants**	Interradicular, infrazygomatic crest, maxillary palate, mandibular buccal shelf	Immediately and delayed	N/A	N/A	Incisor intrusion and retraction
